# Expression Fluctuations of Genes Involved in Carbohydrate Metabolism Affected by Alterations of Ethylene Biosynthesis Associated with Ripening in Banana Fruit

**DOI:** 10.3390/plants9091120

**Published:** 2020-08-30

**Authors:** Yan Xia, Chien-Hsiang Chiu, Yi-Yin Do, Pung-Ling Huang

**Affiliations:** 1 Department of Horticulture and Landscape Architecture, National Taiwan University, Taipei 10617, Taiwan; xyfafu@163.com (Y.X.); r01628127@ntu.edu.tw (C.-H.C.); 2 Graduate Institute of Biotechnology, Chinese Culture University, Taipei 11114, Taiwan

**Keywords:** carbohydrate metabolism, transcriptome, fruit ripening, RNA interference, 1-aminocyclopropane-1-carboxylic acid (ACC) oxidase

## Abstract

The banana is a typical climacteric fruit that undergoes ethylene dependent ripening. During fruit ripening, ethylene production triggers a developmental cascade that results in a series of physiological and biochemical changes. The fruit transcriptomes of untransformated wild-type (WT) and RNAi transgenic banana plants for *Mh-ACO1* and *Mh-ACO2* have been previously sequenced and analyzed, and most of the differentially expressed genes were enriched in ‘carbon fixation in photosynthetic organism’, ‘cysteine and methionine metabolism’, ‘citrate cycle (tricarboxylic acid cycle, TCA cycle)’, and ‘starch and sucrose metabolism’ based on Kyoto Encyclopedia of Genes and Genomes (KEGG) annotation. In this research, we investigated the expression fluctuations of genes involved in carbohydrate metabolism affected by alterations of ethylene biosynthesis associated with ripening in banana fruits. Expression profiles of sucrose synthase, sucrose phosphate synthase, neutral invertase, and acidic invertase/β-fructofuranosidase, as analyzed by Avadis and Trinity, showed that both analyses were complementary and consistent. The overall gene expression tendency was confirmed by the implementation of quantitative real-time reverse transcription-polymerase chain reaction (RT-PCR) with mRNAs of banana fruits in *Mh-ACO1* and *Mh-ACO2* RNAi transgenic plants. These results indicated that altered expression of genes associated with ethylene biosynthesis strongly influenced the expression levels of genes related to starch and sucrose metabolism, as well as the glycolysis pathway in ripening banana fruits.

## 1. Introduction

The bananas (*Musa* spp.) is an important staple food source in subtropical and tropical regions and is the largest herbaceous evergreen monocot belonging to the genus *Musa*. Banana is cultivated as a cash crop and is a major annual crop in Southeast Asia. The sweetness of a ripe banana is contributed by soluble sugar accumulation that is biosynthesized from the starch reserve during the respiratory climacteric rise. The average starch content of banana fruits in the pre-climacteric period is 70–80%. However, the starch is almost completely converted into soluble sugars during ripening, which results in less than 1% starch content at the end of the climacteric period [1,2,3]. Sucrose, glucose, and fructose are main soluble sugars detected in ripening banana [1,4]. Initially, the predominant sugar during banana ripening was sucrose, reaching more than one-tenth of the fresh weight and then total soluble sugars content increased, which indicated that the rate of conversion was high [5]. During the development of a banana, starch and amylopectin accumulate gradually and starch’s synthase (SS; EC2.4.1.21) activity increases until the late stage, remaining constant during starch breakdown (climacteric). However, it almost vanishes during storage and is accompanied by amylopectin and starch granule degradation [6]. Only β-amylase (EC3.3.1.2) activity increased before onset of the respiration peak and along with starch decrease. During banana fruit ripening, starch and sucrose metabolism and glycolysis pathways are influenced by ethylene production [7]. Adenosine diphosphate (ADP)-glucose pyrophosphorylase (AGPase; EC2.7.7.27) is the first rate-limiting enzyme in starch biosynthesis and the AGPase family has been found to be highly expressed in developing fruits. Compared with the high expression of *MaAPS1* at all phases of fruit development and ripening, the activity of MaAPL-3 and -2a increased just during the fruit development stage and early-stage ripening, and the *MaAGPase* expression patterns varied greatly among genotypes [8].

Genes encoding major enzymes involved in starch biosynthesis and degradation have been investigated in several plants such as *Arabidopsis*, potato, rice, lotus, and grass species [9,10]. Sucrose phosphate synthase (SPS; EC2.4.1.14) is supposed to be the key enzyme that regulates the sucrose biosynthesis. Gene expression levels of SPS and sucrose synthase (SUS; EC2.4.1.13) were strongly stimulated by ethylene accumulation [11,12]. The expression level of SPS increased distinctly but showed a very low activity at the onset of the climacteric stage and then steadily increased during the post climacteric stages. Moreover, little content of Fructose 2,6-bisphosphate (Fru(1,6)P2; EC3.1.3.46) was found in unripe fruit but increased markedly with an increased respiration rate [6]. The expression of uridine diphosphoryl (UDP)-glucose pyrophosphorylase (UGPase, EC2.7.7.9) in banana pulp was higher than that in peel and consistent with starch degradation and sucrose synthesis in pulp. Expression of the UGPase gene was induced by exogenous ethylene and sugars, especially sucrose and fructose [13].

In our recent study, transgenic banana plants using RNA interference (RNAi) technology against two ACC oxidase genes—*Mh-ACO1* and *Mh-ACO2*—were individually generated for better understanding on the physiological functions of these two ACC oxidase genes. The ethylene production of banana fruits reduced in RNAi transgenic banana lines compared with the untransformed plants showed gene silencing effects of hairpin-type siRNA expression [14]. Moreover, *Mh-ACO1, Mh-ACO2*, and *Mh-ACS1* genes related to ethylene biosynthesis were down-regulated in ripening fruits of *Mh-ACO1* and *Mh-ACO2* RNAi transgenic bananas [14]. Furthermore, mRNA accumulations of genes involved in ethylene signaling were strongly influenced in RNAi transgenic bananas [14]. Therefore, in this study, we focused on expression patterns and levels of genes involved in ethylene production and carbohydrate metabolism in *Mh-ACO1* and *Mh-ACO2* RNAi transgenic banana plants in the search for a possible link between these two pathways in ripening banana fruits, although both pathways are distinct in terms of biochemical function. We found out that altered expression of genes associated with ethylene biosynthesis strongly influenced the expression levels of genes related to starch and sucrose metabolism in ripening banana fruits.

## 2. Results

### 2.1. Transcriptomic Analysis of As1, As2, and WT in Targeted Silencing of Two Paralogous ACC Oxidase Genes in Banana

**Figure 1:**
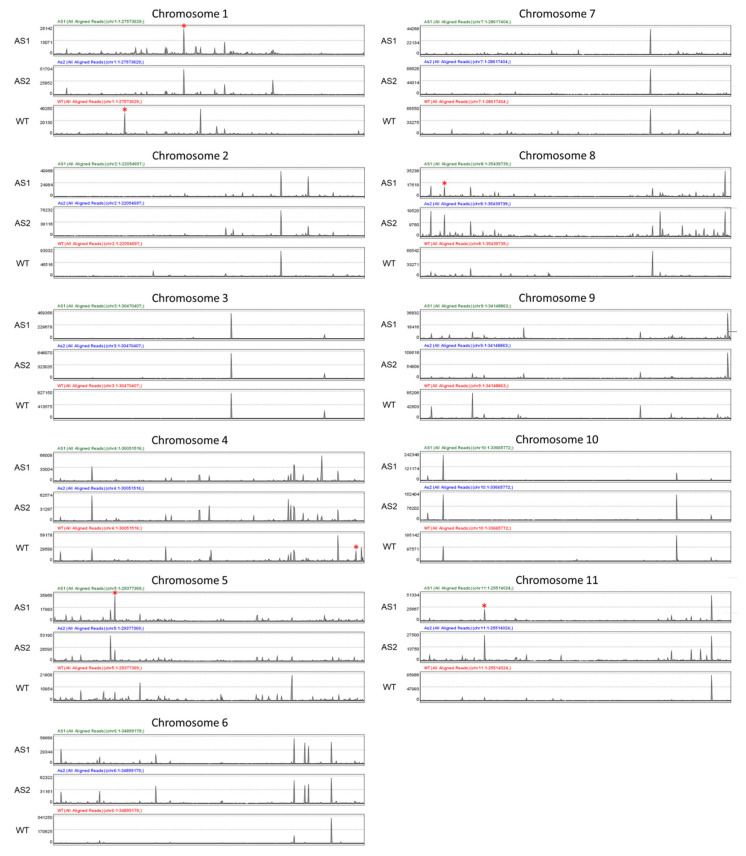
Expression profiles of untransformed wild-type (WT) and two RNAi transgenic banana plants transcriptome RNA-Seq data aligned onto 11 chromosomes of banana by a computationally-optimized Burrows Wheeler transform (called COBWeb) in the Strand NGS software package. The *Y*-axis indicates the relative expression level as represented by the total number of uniquely mapped sequenced reads (or entities) covered alongside each respective chromosome on an *X*-axis scale. Genes involved in carbohydrate metabolism are highlighted by the red star. WT, As1 and As2 represent untransformed control, *Mh-ACO1,* and *Mh-ACO2* RNAi transgenic bananas, respectively.

Global transcriptome of ripening banana fruits from untransformed wild-type (WT) and two RNAi transgenic banana plants targeting either *Mh-ACO1* (As1) or *Mh-ACO2* (As2) had previously been sequenced [14]. For research in this report, the RNA-Seq data were aligned onto 11 chromosomes of banana using the Strand NGS software package in order to visualize the spatial relationships between sequencing enrichment and specific chromosome regions. As shown in Figure 1, these data revealed a similar mapping pattern among the chromosomes of all three samples of WT, As1, and As2, with exactly the same patterns of chromosomes 3 and 7. However, in chromosomes 1 and 5 of samples As1 and As2, apparently distinctive mapping peaks were found compared with the control sample. A detailed gene description for entities with high coverage and significant differential expression in Figure 1 is listed in Table S1. Among these entities, particular genes involved in carbohydrate metabolism were as follows: Granule-bound starch synthase (GSMUA_Achr1G08190_001), putative invertase inhibitor PMEI (GSMUA_Achr1G15410_001), alpha-glucan phosphorylase L isozyme (GSMUA_Achr4G32600_001), putative inactive beta-amylase 9 BAM9 (GSMUA_Achr5G08040_001), alpha-amylase AMY1.1 (GSMUA_Achr8G04140_001), and beta-glucosidase 31 (GSMUA_Achr11G06730_001).

### 2.2. Expression Profiles of Selected Relevant Genes in Starch and Sucrose Metabolism

Based on KEGG (Kyoto Encyclopedia of Genes and Genomes) enrichment analysis in starch and sucrose metabolism, there were 23 gene families in As1 versus WT control detected as differentially expressed, including 9 up-regulated, 8 down-regulated, and 6 families with gene members having either up- or down-regulation of gene expression, while 24 gene families were detected with differential expression in As2-vs-WT, containing 9 up-regulated, 13 down-regulated gene families, and 2 gene families with both up- and down-regulated gene members. In total, there were 25 gene family groups with 441 genes in terms of the number of unigenes subjected to this analysis. Genes in these gene family groups are listed in Table S2.

**Figure 2:**
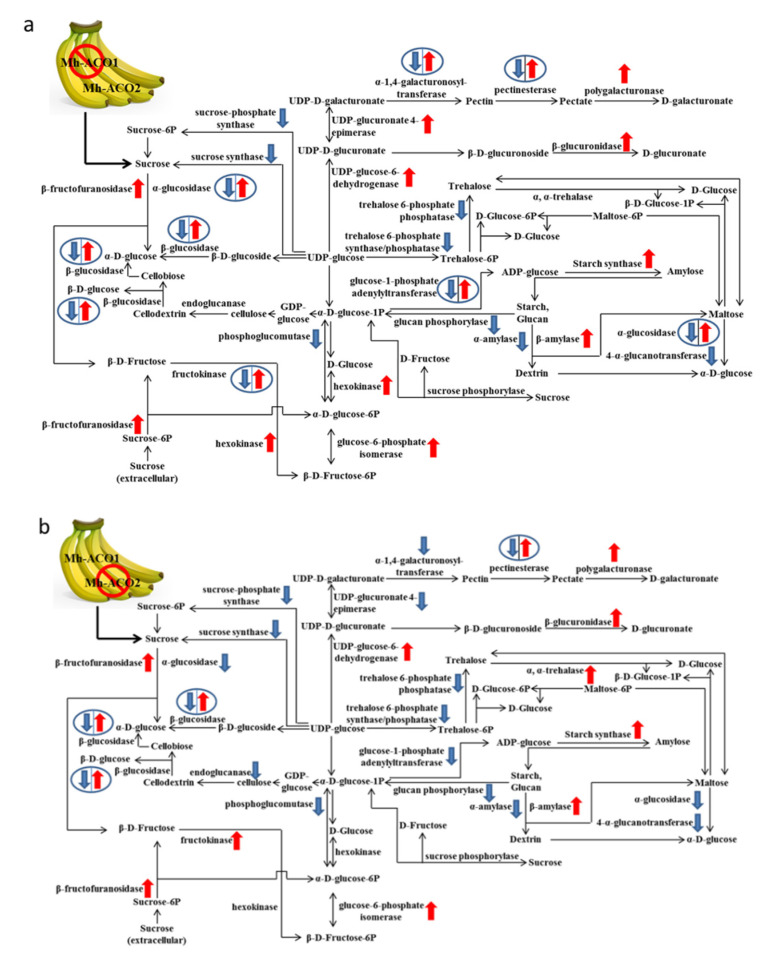

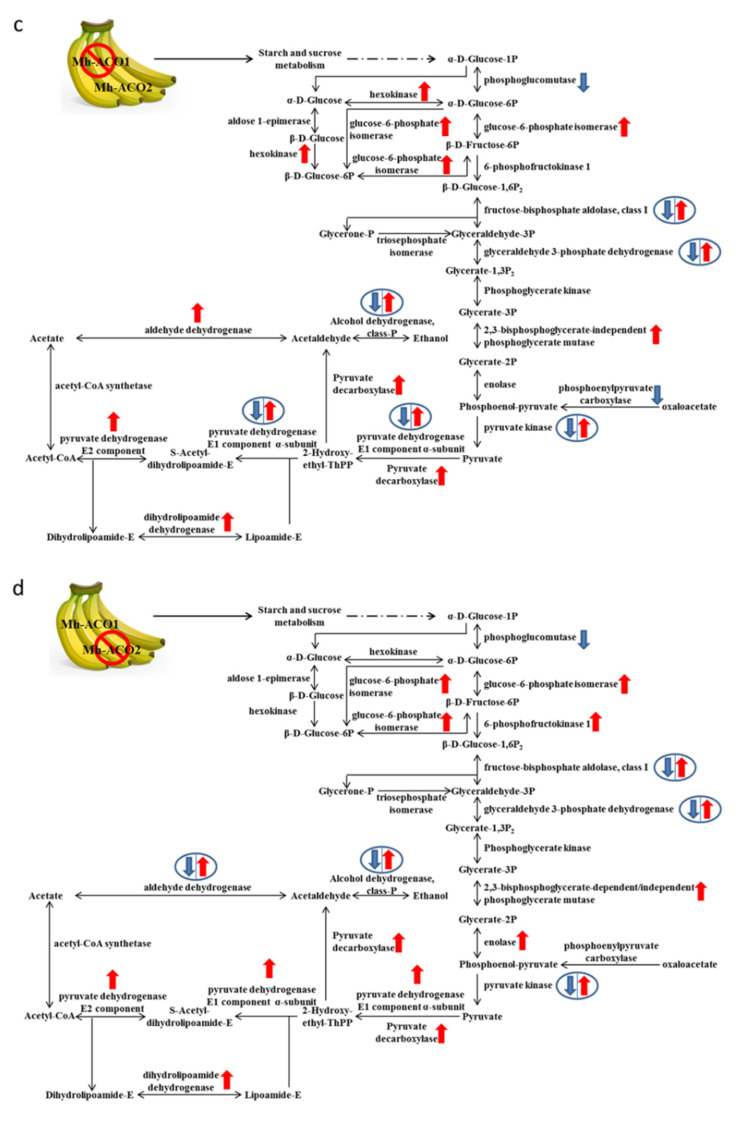
Schematic expression patterns of genes related to carbohydrate metabolism. Enzyme names are indicated between metabolites. Arrowheads with red or blue color indicate differential up- or down-regulation compared with the control WT, respectively. The red and blue arrowheads encircled by a ring indicate that both up- and down-regulated contig ID’s were found. (**a**) Starch and sucrose metabolism pathway in the sample of As1 versus WT control (As1-vs-WT). (**b**) Starch and sucrose metabolism pathway in the sample of As2 versus WT control (As2-vs-WT). (**c**) Glycolysis and gluconeogenesis pathway in the sample of As1-vs-WT. (**d**) Glycolysis and gluconeogenesis metabolism pathway in the sample of As2-vs-WT. WT, As1, and As2 represent untransformed control, *Mh-ACO1,* and *Mh-ACO2* RNAi transgenic bananas, respectively.

Schematic expression patterns of genes related to starch and sucrose metabolism are shown in Figure 2 for the samples of As1 versus WT control (As1-vs-WT; Figure 2a) and samples of As2 versus WT control (As2-vs-WT; Figure 2b). As shown in Figure 2a,b, the gene expressions for sucrose synthase, sucrose phosphate synthase, glucan phosphorylase, and trehalose 6-phosphate synthase were detected down-regulated in both As1-vs-WT and As2-vs-WT, whereas gene expressions for polygalacturonase, β-fructofuranosidase, β-amylase, glucose-6-phosphate isomerase, and UDP-glucose-6-dehydrogenase were found up-regulated in both As1-vs-WT and As2-vs-WT. Among genes involved in starch and sucrose metabolism, α-amylase gene was found down-regulated only in As1-vs-WT and showed no significant change in As2-vs-WT. The endoglucanase gene was found down-regulated in As2-vs-WT and maintained the same expressions in As1-vs-WT. The gene for UDP-glucuronate 4-epimerase was found up-regulated in As1-vs-WT (Figure 2a) and down-regulated in As2-vs-WT (Figure 2b).

Moreover, the gene expressions of endoglucanase (EC3.2.1.4) and alpha, alpha-trehalase (EC3.2.1.28) were detected down-regulated and up-regulated, respectively, in As2-vs-WT, whereas these two gene families showed no change in gene expression in As1-vs-WT. Moreover, the expressions of hexokinase (EC2.7.1.1) gene family were only up-regulated in As1-vs-WT and showed no differences in As2-vs-WT.

### 2.3. Expression Profiles of Selected Relevant Genes in Glycolytic Metabolism

A total of 15 differentially expressed gene families were found to participate in glycolytic metabolism in As1-vs-WT, among which there were 8 up-regulated and 2 down-regulated gene families with 5 gene families containing either up- or down-regulated gene members. The number of differentially expressed gene families in the glycolysis pathway was 16 in As2-vs-WT, and these gene families consisted of 10 up-regulated, 1 down-regulated gene families, and 5 gene families with either up- or down-regulated members. In total, there were 20 gene families with 257 genes in terms of the number of unigene IDs subjected to this analysis. Genes in these gene families are listed in Table S3.

The most significantly enriched up-regulated DEGs in As1-vs-WT were gene families for 6-phosphofructokinase, 2,3-bisphosphoglycerate-independent phosphoglycerate mutase, pyruvate decarboxylase, pyruvate dehydrogenase E2 component, and dehydrolipoamide dehydrogenase (Figure 2c). Gene families for class I fructose-bisphosphate aldolase, aldehyde dehydrogenase, alcohol dehydrogenase, pyruvate dehydrogenase, E1 component, pyruvate kinase, and glyceraldehyde-3-phosphate dehydrogenase showed both up- and down-regulated gene expression members in As1-vs-WT.

Gene families for 6-phosphofructokinase, 2,3-bisphosphoglycerate-independent phosphoglycerate mutase, pyruvate decarboxylase, enolase, pyruvate dehydrogenase E1 component, and dihydrolipoamide dehydrogenase were up-regulated in As2-vs-WT (Figure 2d). Some gene families, such as genes for class I fructose-bisphosphate aldolase, glyceraldehyde-3-phosphate dehydrogenase, pyruvate kinase, aldehyde dehydrogenase, and alcohol dehydrogenase were detected containing both up- and down-regulated gene members in As2-vs-WT, as shown in Figure 2d.

Gene expression variations between As1 and As2 in glycolysis metabolism were found (Figure 2c,d). The genes that encode 6-phosphofructokinase 1 (EC2.7.1.11), 2,3-bisphosphoglycerate-dependent phosphoglycerate mutase (EC5.4.2.11), and enolase (EC4.2.1.11) were up-regulated in As2-vs-WT, while in As1-vs-WT only up-regulation of the gene family for 2,3-bisphosphoglycerate-dependent phosphoglycerate mutase was found. Moreover, up-regulation of hexokinase gene family and down-regulation of phosphoenolpyruvate carboxylase (EC4.1.1.32) gene family were detected only in As1-vs-WT but not in As2-vs-WT. For expression of the enolase gene, there was no significant change in As1-vs-WT but significant up-regulation was observed in As2-vs-WT. For expression of the gene family of pyruvate dehydrogenase E2 component, there was up-regulation in As1-vs-WT, while there was no change in As2-vs-WT. The gene family of pyruvate dehydrogenase E1 component contained both up-and down-regulation in As1-vs-WT, but only up-regulation was detected in As2-vs-WT (Figure 2c,d).

### 2.4. Gene Expressions of Sucrose Synthase, Sucrose Phosphate Synthase, and Neutral Invertase as Analyzed by Avadis and Trinity

**Figure 3:**
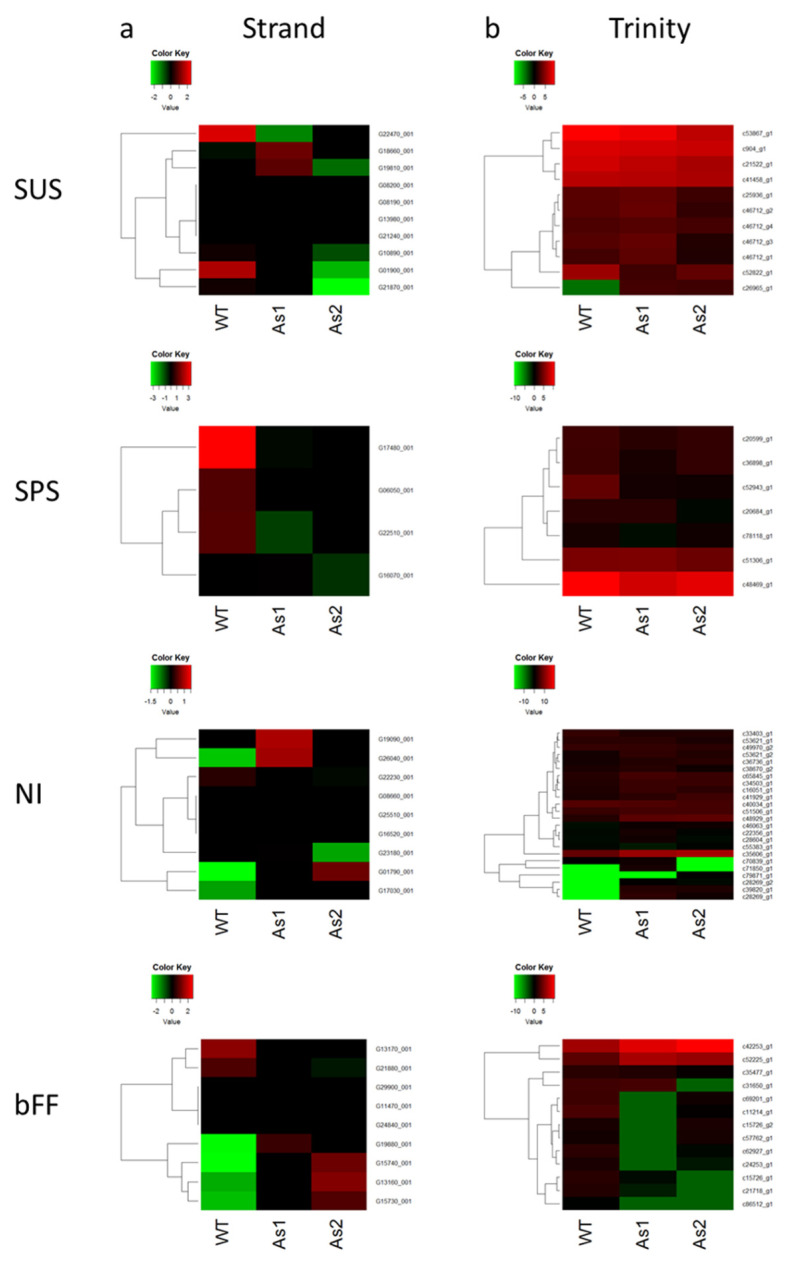
Differentially expressed genes of sucrose synthase (SUS), sucrose phosphate synthase (SPS), neutral invertase (NI), and acidic invertase/β-fructofuranosidase (bFF) in *Mh-ACO1* RNAi (As1), *Mh-ACO2* RNAi (As2), and the control (WT) banana plants. The analyzed softwares are Strand NGS software package (Avadis) (**a**) and Trinity package (**b**). To highlight the relative differences between plants, log2 transformed fold change values for each contig are displayed. The detailed data are given in Tables S2 and S3.

Banana fruits have the typical climacteric behavior with an outbreak in ethylene production to initiate the ripening process. In order to gain a deeper insight into molecular mechanism of banana fruit ripening, the genes related to carbohydrate metabolism were conducted for further comprehensive studies. Sucrose and carbohydrate accumulation are imported into banana fruits in the form of starch during banana ripening [15,16]. Once ripening has been triggered, the banana fruits ripen quickly and sweeten due to conversion of starch-degraded products to soluble sugars [17,18]. Moreover, the invertase gene family is particularly interesting. There are two types of invertases: one is acidic, targeted to the cell wall and vacuole, the other one neutral/alkaline functioning in the cytosol. In order to investigate the gene activities of sucrose-phosphate synthase, sucrose synthase, neutral invertase (NI, EC3.2.1.26), and acidic invertase/β-fructofuranosidase (bFF, EC3.2.1.26) involved in the carbohydrate metabolic pathway, the gene expression profiles for these genes were analyzed by two different analysis software: Avadis and Trinity. The contigs analyzed by Trinity and their corresponding genes in the Banana Genome Hub are listed in Tables S2 and S3, respectively, and their expressions visualized by heat map according to fragments per kilobase to transcript per million mapped reads (FPKM) value (Tables S4 and S5) are shown in Figure 3, respectively. In this analysis, expression levels of contigs c25936_g1, c46712_g1, c46712_g1, c46712_g3, c52822_g1, and c26965_g1, corresponding to sucrose synthase, c36898_g1, c52943_g1, c20684_g1, and c78118_g1, corresponding to sucrose phosphate synthase, and c70839_g1, c71850_g1, and c79871_g1, corresponding to neutral invertase, showed apparently different among WT, As1, and As2 banana samples (Figure 3), and the expression profiles for these genes showed similar tendency based on reads per kilobases per million reads (RPKM) value (Tables S4 and S5). In the analysis with Avadis, data of RNA-Seq were aligned onto banana chromosomes for the comparison among samples with normalization of RPKM. The entities covered on the specific positions corresponding to the genes of sucrose synthase, sucrose-phosphate synthase, neutral invertase, and acidic invertase/β-fructofuranosidase on aligned chromosomes are listed in Table S6, and their gene expressions visualized by a heat map are shown in Figure 3. Entitis corresponded to the sucrose synthase gene, sucrose phosphate synthase, and neutral invertase also expressed differentially among WT, As1, and As2 banana samples are shown in Figure 3. The overall expression tendency of differentially expressed genes analyzed by Trinity is in agreement with those analyzed in Strand NGS. It is worthy of further investigation on the roles of these differentially expressed genes in fruit ripening.

### 2.5. Expression Profiles of the Sucrose Biosynthesis Pathway Genes Based on Real-Time Quantitative Reverse Transcription Polymerase Chain Reaction (qRT-PCR) Data

**Figure 4:**
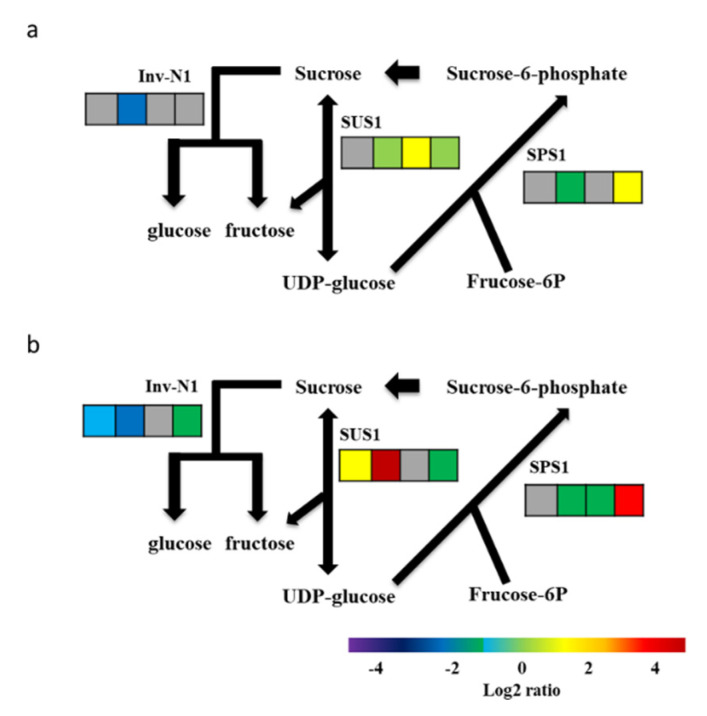
Expression patterns of the sucrose biosynthetic pathway genes in *Mh-ACO1* (**a**) and *Mh-ACO2* (**b**) *RNAi* transgenic banana fruits. Relative mRNA abundances were visualized based on qRT-PCR data. Four continuous blocks from left to right indicate that fruits were harvested at stages 1, 3, 5, and 7. Log2 transformed values of relative transcript abundance was normalized with the wild type banana. Gene expression levels are shown with a rainbow color scale. Red indicates high relative expression, yellow indicates moderate relative expression, and green indicates low relative expression. Genes without differential expression between transgenic banana and wild type are shown by grey boxes. The experiment was performed in two biological and three technical replicates. SUS1, sucrose synthase. SPS1, sucrose phosphate synthase. Inv-N1, neutral invertase.

Quantitative RT-PCR, using total RNAs from transgenic banana fruits, was performed to validate the expression profiles of the genes involved in sucrose biosynthesis in *Mh-ACO1* and *Mh-ACO2* RNAi transgenic bananas. The expression patterns of all selected genes based on qRT-PCR (Table S7) were similar to those detected by high-throughput sequencing. The overall tendency of these expression messages was clearly down-regulated (albeit not apparent), such as the patterns shown at stages 1 and 3 for neutral invertase gene *Inv-N1* in both As1 and As2, and up-regulated at all stages tested for genes of sucrose synthase 1 (SUS1) and sucrose-phosphate synthase 1 in both As1 and As2 (Figure 4). Moreover, in samples As1 and As2, the following observations were made, as shown in Figure 4: (1) a reduction in gene expression levels of Inv-N1 at stage 3 in As1 and at stages 1, 3, and 7 in As2; (2) a significant increase in gene expression levels of SUS1 at stage 5 in As1 and at stages 1 and 3 in As2; and (3) a significant increase in gene expression levels of SPS1 at stage 7 in both As1 and As2. Interestingly, gene expression of up-, down-, and up again- patterns was present in As1 for SUS1, where the gene expression was decreased at stage 3, increased at stage 5, and decreased again at stage 7. As for the gene expression of SPS1 in As1, it decreased at stage 3 and increased again at stage 7. In the sample of As1, the gene expression of Inv-N1 slightly decreased at stage 3 when compared to WT, and showed no obvious change between stages 5 and 7. Moreover, in the sample of As2, the gene expression of Inv-N1 was significantly down-regulated at stage 3, whereas expression of the gene SUS1 at stages 1 and 3 and SPS1 at stage 7 were significantly up-regulated (Figure 4). These results suggest that carbohydrate metabolism-related genes expressed during ripening may be controlled by ethylene biosynthesis-related genes in banana fruits.

## 3. Discussion

Banana fruits are the most popular fresh fruits worldwide with great economic value. During ripening of banana fruits, softening, discoloration, and decay make the fruits become fragile and lose value. In order to delay ripening and increase postharvest quality and storability of banana fruits, RNA interference against banana ACC oxidase genes had been applied to modify ethylene biosynthesis [14]. Most of the up-regulated DEGs in RNAi transgenic bananas are involved in starch and sucrose metabolism, glycolysis/gluconeogenesis, carbon metabolism, and biosynthesis of amino acids in KEGG enrichment analysis, indicating a close relationship between ethylene production and carbohydrate metabolism. According to previous studies, carbohydrate metabolism during banana fruit ripening is accelerated by the elevated activity of enzymes such as amylase, glycosidase, starch phosphorylase, sucrose synthase, sucrose phosphate synthase, and invertase, leading to increased enzyme activity in the pentose phosphate pathway and the formation and accumulation of monosaccharides in banana fruits [19]. Activities of enzymes participating in starch-sucrose interconversion such as sucrose synthase, sucrose phosphate synthase, and neutral invertase result in the degradation of the starch reserve and the formation of hexose sugars during banana fruit ripening. Furthermore, the carbohydrate properties of the storage organs affect the quality of banana fruits. However, the linkage between ethylene synthesis and ethylene-regulated expression of genes involved in starch and carbohydrate metabolism, one of the major criteria of fruit quality, has been far less investigated. Here, we show that the genes participating in carbohydrate metabolism were also affected by ethylene biosynthesis associated with ripening banana fruits.

Starch synthases catalyze amylose biosynthesis in higher plants [20]. In both As1 and As2, the expression of starch synthase genes was higher than those in WT samples (Figure 2a,b), indicating that the level of the amylose content in the banana fruits may be higher in both As1 and As2. According to our results, sucrose synthase and sucrose-phosphate synthase catalyzing sugar-starch conversion steps and a cytosolic neutral invertase may be key regulators for starch accumulation in the ripening banana fruits. This finding provides useful information for future investigation focused on clarifying the molecular mechanisms of determination of the starch properties in banana fruits.

More specifically, the following events have been evident occurring in the samples of As1 and As2. (1) The gene expressions for sucrose synthase and sucrose phosphate synthase were down-regulated, and with the increase in the gene expression level of β-fructofuranosidase, sucrose was decomposed into α-D-glucose and β-D-fructose in *Mh-ACO1* and *Mh-ACO2* RNAi transgenic banana lines, resulting in the reduction of sucrose accumulation. (2) Glucan phosphorylase in plants is usually called starch phosphorylase and the gene family for glucan phosphorylase showed down-regulated expression in As1 and As2, which reduced the phosphorylation process of starch. Moreover, the up-regulation of β-amylase genes accelerated the rate of starch decomposition into maltose. (3) Gene expression for the gene family of sucrose phosphorylase did not change, so the catalysis of sucrose transformation into D-fructose was not affected. (4) Under the action of phosphate catalytic fructose kinase, β-D-fructose-6 phosphate may be increasingly produced from β-D-fructose through the up-regulation of genes for hexokinase in As1 (Figure 2a) or fructokinase in As2 (Figure 2b). (5) It was found out that the genes for UDP-glucose-6-dehydrogenase, polygalacturonase, and β-glucuronidase were up-regulated, implying that the pectate, D-galacturonate, and D-glucuronate may be enriched in the *Mh-ACO1* and *Mh-ACO2* transgenic banana plants. (6) The breakdown of cellulose to soluble sugars involves the action of a multi-enzyme system. Cellulose is degraded by the synergistic action of three types of enzymes, namely endoglucanase (EC3.2.1.4), exoglucanase (EC3.2.1.91), and β-glucosidase (EC3.2.1.21). It was found out that the gene expression for endoglucanase was down-regulated in As2, implying that the synthesis of D-cellobiose might be reduced. However, this situation was not occurring in As1. (7) The starch decomposition into dextrin might be reduced because the gene expression for α-amylase was down-regulated in both As1 and As2.

The increase in gene expressions of polygalacturonase and β-glucuronidase, both in As1 and As2, as shown in Figure 2a,b, respectively, indicated that the structure of pectin and other hexuronates (D-galacturonate and D-glucuronate) might be changed in banana peels and pulps of As1 and As2. D-galacturonate and D-glucuronate are the sugar constituents of pectic polysaccharides which constitute a dominant portion of the primary cell walls of higher plants [21].

In total, 21 gene families involved in glycolysis and gluconeogenesis [22] were monitored during ripening of banana fruits in As1 and As2. Out of 21 gene families coding for enzymes involved in glycolysis/gluconeogenesis pathway and pyruvate metabolism, 15 gene families (accounting for 71%) were found to be up-regulated in gene expression in both As1 and As2.

The rate-limiting enzymes of the glycolysis and gluconeogenesis pathway were hexokinases, 6-phosphofructokinase 1, and pyruvate kinase [7]. From a comparison of all the enzymes involved in glycolysis, it appears that hexokinase in As1 and 6-phosphofructokinase 1 in As2 have higher gene expression levels in relation to WT levels. While gene expression for enolase remained unaffected in As1 (Figure 2c), the expression level of enolase gene was up-regulated in As2 (Figure 2d), implying that in As2, the efficiency of enzymes directly participating in glycolysis and glucose consumption may be increased.

The gene expression levels for hexokinase in As1 and for 6-phosphofructokinase [23,24] in As2 were increased, implying that the breakdown of phosphohexoses and hence the synthesis of pyruvate and ATP may be accelerated both in As1 and As2. This viewpoint was further enhanced in that point the pathway of carbon metabolism through phosphorylation was catalyzed by enolase in As2 (Figure 2d).

Taking all these results into accounts, the silencing of either *MhACO1* or *MhACO2* gene, and hence the decrease in ethylene biosynthesis in both As1 and As2, may affect the breakdown of phosphohexoses and also may directly or indirectly elevate the levels of pyruvate and ATP.

## 4. Materials and Methods

### 4.1. Plant Materials

The *Musa* spp. cv. Pei-Chiao (AAA group, Cavendish subgroup) was used. Untransformed (WT) and *Mh-ACO1* (As1) and *Mh-ACO2* (As2) RNAi transgenic banana plants were obtained as previously described [14]. They were grown in a planting medium including soil, peat, and sand at a proportion of 1:1:1 by volume in a 300 dm^3^ plastic tank. The banana plants were cultivated to flower and set fruits under an isolated greenhouse. The banana fruits were harvested roughly 15 weeks after flower emergence when the fruits had developed close to maturity. Banana fruit ripening is divided into stages 1 to 8 by peel color according to the CSIRO Banana Ripening Guide: all green, green with trace yellow, more green than yellow, more yellow than green, green tip, all yellow, yellow flecked with brown, and yellow with much brown, respectively [25]. The harvested fruits were treated as previously described [26].

### 4.2. Total RNA Extraction and Whole-Transcriptome Deep Sequencing

To analyze transcriptomic changes, fruits were harvested from As1, As2, and WT plants at ripening stage 3. Total RNA from pulp and peel of banana was extracted using NEBNext^®^ Ultra^TM^ RNA Library Prep Kit for Illumina^®^ (NEB, Ipswich, MA, USA). The integrity of RNA samples was estimated by electrophoresis on a 1.2% agarose gel. The integrated RNA samples consisting of a mixture of equal amounts of RNA from banana pulp and peel were used for transcriptome analysis using HiSeq^TM^ 2500 Illumina to generate paired-end sequencing reads. The cleaned reads were assembled into unigenes using Trinity [27] after adaptors and low-quality sequences were removed. The generated unigenes were assigned functional annotations by similarity searches with Blastx against protein databases nr, nt, and SWISS-PROT. The best hits obtained from the blast against the nr database using Blastx2GO were used to extract Gene Ontology (GO) (E-value < 10^−6^). Blastx was also used to align unique sequences to the KEGG (E-value < 10^−10^). Normalized read counts adjusted to FPKM were used to identify differentially expressed unigenes using DEGSeq from the R Statistical software package [28], with a threshold of FDR < 0.05 and log_2_ Ratio ≥ 1. [log_2_ (Fold Change)] > 1 and q-value < 0.005 were used as the threshold to identify and compare differentially expressed genes (DEGs) in As1-vs-WT, As2-vs-WT, and As1-vs-As2. To quantify the relative gene expressions by heat map visualization normalized by FPKM value (Tables S2 and S3), only those IDs specifically matching to genes that were annotated in the Banana Genome Hub with pairwise identity of greater than 95% as a cutoff using BlastN (as shown in Tables S2 and S3) were used. Tables S2 and S3 list the contig names corresponding to genes present in the Banana Genome Hub.

### 4.3. Real-Time Quantitative RT-PCR

The quantification of the transcripts of selected candidate genes related to starch and sucrose metabolism was performed by real-time quantitative RT-PCR (qRT-PCR) using the cDNA synthesized from RNA samples of banana fruits at ripening stages 1, 3, 5, and 7 by ProtoScript^®^ First Strand cDNA Synthesis Kit (NEB, Ipswich, MA, USA). The sequences of primers used in qRT-PCR are listed in Table S8. The relative expression level was calculated according to the 2^−ΔΔCt^ formula [29]. Expression levels in Ct values of the candidate genes were first normalized to the expression levels of *ACTIN* and then to the respective expression levels in WT plants. Each experiment had three replicates. Heat maps [14] were applied to visualize the relative mRNA abundances.

### 4.4. R Programming for Heat Map Illustration

The graphical visualization of expression profiles of genes associated in carbohydrate and sucrose metabolism in As1 and As2 transgenic banana plants was plotted as heat maps with R programming language within the Gplots package [30]. In WT, As1, and As2 unigene libraries from transcriptome sequencing after performing RNA-Seq, the number of the final assembled contig IDs was 88,031. To create heat maps for those IDs that specifically matched to the annotated genes in the Banana Genome Hub with pairwise sequence comparison exceeding 95% using BlastN were used.

## 5. Conclusions

In this study, the fruit transcriptomes of WT and RNAi transgenic banana plants for *Mh-MACO1* and *Mh-ACO2* were analyzed, revealing that the expression of genes involved in carbohydrate metabolism were affected by alterations of ethylene biosynthesis associated with ripening in banana fruits. Expression profiles of genes relevant to starch and sucrose metabolism, as well as of genes involved in glycolysis pathway, were thoroughly analyzed. The overall gene expression tendency through different ripening stages was validated by the implementation of quantitative real-time RT-PCR with mRNAs of banana fruits in *Mh-ACO1* and *Mh-ACO2* RNAi transgenic plants. Future work needs to further elucidate the global metabolomics examinations among WT, *Mh-ACO1,* and *Mh-ACO2* RNAi transgenic banana plants. These studies will provide new insights into the mechanisms and consequences of ethylene biosynthesis-related genes in carbohydrate metabolism during banana fruit ripening.

## Supplementary Materials

The following are available online at https://www.mdpi.com/2223-7747/9/9/1120/s1, Table S1: Gene ID, reads per kilobase of transcript per million mapped reads (RPKM) in log2 value and gene description for entities with high coverage and significantly differential expression in Figure 1; Table S2: Genes in starch and sucrose metabolism used for Blast analysis in this study; Table S3: Genes in glycolysis and gluconeogenesis used for Blast analysis in this study; Table S4: The fragments per kilobase of transcript per million mapped reads (FPKM) values of selected relevant genes in starch and sucrose metabolism which were applied in this study; Table S5: The fragments per kilobase of transcript per million mapped reads (FPKM) values of selected relevant genes in glycolysis and gluconeogenesis which were applied in this study; Table S6: The entities covered on the specific positions corresponding to the genes of sucrose; Table S7: Relative mRNA abundances based on qRT-PCR data in *Mh-ACO1* RNAi (As1) and *Mh-ACO2* RNAi (As2) transgenic and untransformed control (WT) banana pulps; Table S8: Primers used in qRT-PCR for genes related to sucrose biosynthesis.
