# Supplementary material for: Expression Fluctuations of Genes Involved in Carbohydrate Metabolism Affected by Alterations of Ethylene Biosynthesis Associated with Ripening in Banana Fruit
Source: Plants (Basel). 2020 Aug 30;9(9):1120. doi: 10.3390/plants9091120 (PMC7570234; doi:10.3390/plants9091120)
Supplement: Supplementary file 1 [file plants-09-01120-s001.zip › Table_S8.docx]

Table S8. Primers used in qRT-PCR for genes related to sucrose biosynthesis.

| Gene | Primer name | Sequence (5’→3’) |
| --- | --- | --- |
| *SUS1*  chr10:25291305..25295732 | SUS-F | AACTACAAGGGCATGTCGATG |
|  | SUS-R | GGTGTCAGATGCGATACTCAATAG |
| *SPS1*  chr4:14252455..14262207 | SPS-F | ACCGATATGGTCTGAGGTTATGC |
|  | SPS-R | TAGCATTGCCGGTAGACATC |
| *Inv-N1*  chr5:15850607..15854239 | *Inv-N1*-5 | GCTTTGTGTTTGTCCGAAGG |
|  | *Inv-N1*-3 | GTCTTGCTTCAGCATCGACA |
| *Actin*  *GeneBank AF246288* | *BACT5* | TAGCGACGTACCACAGGTAT |
|  | *BACT3* | GTAAGCAAGCTTCTCCTTGAT |
