# Supplementary material for: Expression Fluctuations of Genes Involved in Carbohydrate Metabolism Affected by Alterations of Ethylene Biosynthesis Associated with Ripening in Banana Fruit
Source: Plants (Basel). 2020 Aug 30;9(9):1120. doi: 10.3390/plants9091120 (PMC7570234; doi:10.3390/plants9091120)
Supplement: Supplementary file 1 [file plants-09-01120-s001.zip › Table_S2.docx]

# Table S2. Genes in starch and sucrose metabolism used for Blast analysis in this study. The gene annotations are obtained from Banana Genome Hub.

| Gene Name  Used in This  Research | Gene ID In  Banana Genome  Hub | Gene  Location | Start | End | Gene Annotation |
| --- | --- | --- | --- | --- | --- |
| 4-alpha-  glucanotransferase | Ma02_p23910.1 | chr02 | 28720064 | 28725527 | Ma02_g23910~ 4-alpha-glucanotransferase DPE1, chloroplastic/amyloplastic~unknown_gene~ missing_functional_completeness |
| 4-alpha-  glucanotransferase | Ma03_p08680.1 | chr03 | 6388263 | 6397980 | Ma03_g08680~ 4-alpha-glucanotransferase DPE2, transcript variant X1~ unknown_gene~ missing_functional_completeness |
| 4-alpha-  glucanotransferase | Ma03_p08680.2 | chr03 | 6388263 | 6397980 | Ma03_g08680~ 4-alpha-glucanotransferase DPE2, transcript variant X1~ unknown_gene~ missing_functional_completeness |
| alpha,alpha-trehalose-phosphate synthase | Ma03_p06080.1 | chr03 | 4194533 | 4203282 | Ma03_g06080~ Putative Probable alpha,alpha-trehalose-phosphate synthase [UDP-forming] 9~ TPS9~ missing_completeness |
| alpha,alpha-trehalose-phosphate synthase | Ma03_p07500.1 | chr03 | 5273571 | 5276313 | Ma03_g07500~ alpha,alpha-trehalose-phosphate synthase [UDP-forming]  6-like~ unknown_gene~ missing_functional_completeness |
| alpha,alpha-trehalose-phosphate synthase | Ma05_p20310.1 | chr05 | 31951693 | 31954538 | Ma05_g20310~ alpha,alpha-trehalose-phosphate synthase [UDP-forming] 5, transcript variant X1~ unknown_gene~ missing_functional_completeness |
| alpha,alpha-trehalose-phosphate synthase | Ma05_p20310.2 | chr05 | 31951693 | 31954538 | Ma05_g20310~ alpha,alpha-trehalose-phosphate synthase [UDP-forming] 5, transcript variant X1~ unknown_gene~ missing_functional_completeness |
| alpha,alpha-trehalose-phosphate synthase | Ma05_p20310.3 | chr05 | 31951693 | 31954538 | Ma05_g20310~ alpha,alpha-trehalose-phosphate synthase [UDP-forming] 5, transcript variant X1~ unknown_gene~ missing_functional_completeness |
| alpha,alpha-trehalose-phosphate synthase | Ma05_p20310.4 | chr05 | 31951693 | 31954538 | Ma05_g20310~ alpha,alpha-trehalose-phosphate synthase [UDP-forming] 5, transcript variant X1~ unknown_gene~ missing_functional_completeness |
| alpha,alpha-trehalose-phosphate synthase | Ma06_p16230.1 | chr06 | 10983150 | 10985919 | Ma06_g16230~ probable alpha,alpha-trehalose-phosphate synthase [UDP-forming]  7~ unknown_gene~ missing_functional_completeness |
| alpha,alpha-trehalose-phosphate synthase | Ma06_p27850.1 | chr06 | 29705284 | 29725549 | Ma06_g27850~ alpha,alpha-trehalose-phosphate synthase [UDP-forming] 1-like, transcript variant X2~ unknown_gene~ missing_functional_completeness |
| alpha,alpha-trehalose-phosphate synthase | Ma06_p27850.2 | chr06 | 29705284 | 29725549 | Ma06_g27850~ alpha,alpha-trehalose-phosphate synthase [UDP-forming] 1-like, transcript variant X2~ unknown_gene~ missing_functional_completeness |
| alpha,alpha-trehalose-phosphate synthase | Ma07_p11010.1 | chr07 | 8160210 | 8162993 | Ma07_g11010~ alpha,alpha-trehalose-phosphate synthase [UDP-forming] 6-like~unknown_gene~ missing_functional_completeness |
| alpha,alpha-trehalose-phosphate synthase | Ma07_p16080.1 | chr07 | 12463630 | 12467468 | Ma07_g16080~ probable alpha,alpha-trehalose-phosphate synthase [UDP-forming] 9~ unknown_gene~ missing_functional_completeness |
| alpha,alpha-trehalose-phosphate synthase | Ma07_p26280.1 | chr07 | 33143305 | 33146486 | Ma07_g26280~ probable alpha,alpha-trehalose-phosphate synthase [UDP-forming] 7~ unknown_gene~ missing_functional_completeness |
| invertase | Ma01_p08810.1 | chr01 | 6316776 | 6317345 | Ma01_g08810~ putative invertase inhibitor~ unknown_gene~missing_functional_completeness |
| invertase | Ma01_p08810.1 | chr01 | 6316776 | 6317345 | Ma01_g08810~ putative invertase inhibitor~ unknown_gene~missing_functional_completeness |
| invertase | Ma01_p18430.1 | chr01 | 13676457 | 13676993 | Ma01_g18430~ putative invertase inhibitor~ unknown_gene~missing_functional_completeness |
| invertase | Ma02_p03720.1 | chr02 | 15137159 | 15141931 | Ma02_g03720~ plant neutral invertase domain containing protein, expressed~ unknown_gene~missing_completeness |
| invertase | Ma08_p22930.1 | chr08 | 36412198 | 36418249 | Ma08_g22930~ alkaline/neutral invertase CINV2-like~ unknown_gene~ missing_functional_completeness |
| invertase | Ma02_p13890.1 | chr02 | 22077936 | 22078472 | Ma02_g13890~ putative invertase inhibitor~ unknown_gene~missing_functional_completeness |

**Table S2**.*Cont*.

| Gene Name  Used in This  Research | Gene ID In  Banana Genome  Hub | Gene  Location | Start | End | Gene Annotation |
| --- | --- | --- | --- | --- | --- |
| invertase | Ma02_p13910.1 | chr02 | 22083299 | 22083835 | Ma02_g13910~ putative invertase inhibitor~ unknown_gene~missing_functional_completeness |
| invertase | Ma03_p13920.1 | chr03 | 10970271 | 10970828 | Ma03_g13920~ putative invertase inhibitor~ unknown_gene~missing_functional_completeness |
| invertase | Ma03_p13970.1 | chr03 | 11114349 | 11114882 | Ma03_g13970~ putative invertase inhibitor~ unknown_gene~missing_functional_completeness |
| invertase | Ma03_p13980.1 | chr03 | 11130675 | 11131211 | Ma03_g13980~ putative invertase inhibitor~ unknown_gene~missing_functional_completeness |
| invertase | Ma03_p13990.1 | chr03 | 11170834 | 11171373 | Ma03_g13990~ putative invertase inhibitor~ unknown_gene~missing_functional_completeness |
| invertase | Ma03_p19780.1 | chr03 | 25035083 | 25038898 | Ma03_g19780~ neutral/alkaline invertase, putative, expressed~ ints3~ missing_completeness |
| invertase | Ma03_p19940.1 | chr03 | 25200019 | 25203320 | Ma03_g19940~ plant neutral invertase domain containing protein, expressed~unknown_gene~missing_completeness |
| invertase | Ma04_p04520.1 | chr04 | 3453964 | 3454728 | Ma04_g04520~ putative invertase inhibitor~ unknown_gene~ issing_functional_completeness |
| invertase | Ma04_p11300.1 | chr04 | 7938376 | 7938912 | Ma04_g11300~ putative invertase inhibitor~ unknown_gene~missing_functional_completeness |
| invertase | Ma04_p11620.1 | chr04 | 8208849 | 8209406 | Ma04_g11620~ putative invertase inhibitor~ unknown_gene~missing_functional_completeness |
| invertase | Ma04_p25320.1 | chr04 | 27136971 | 27137525 | Ma04_g25320~ putative invertase inhibitor~ unknown_gene~missing_functional_completeness |
| invertase | Ma04_p25340.1 | chr04 | 27142315 | 27142869 | Ma04_g25340~ putative invertase inhibitor~ unknown_gene~missing_functional_completeness |
| invertase | Ma05_p18400.1 | chr05 | 23798028 | 23799964 | Ma05_g18400~ plant neutral invertase domain containing protein, expressed~ gcvT~missing_completeness |
| invertase | Ma05_p20490.1 | chr05 | 32170997 | 32171512 | Ma05_g20490~ putative invertase inhibitor~ unknown_gene~missing_functional_completeness |
| invertase | Ma05_p28410.1 | chr05 | 39430839 | 39433905 | Ma05_g28410~ Alkaline/neutral invertase~ unknown_gene~ missing_completeness |
| invertase | Ma06_p18710.1 | chr06 | 12784923 | 12790131 | Ma06_g18710~ neutral/alkaline invertase, putative, expressed~ ttc26~ missing_completeness |
| invertase | Ma06_p27280.1 | chr06 | 29334746 | 29336693 | Ma06_g27280~ plant neutral invertase domain containing protein, expressed~ gcvT~ missing_completeness |
| invertase | Ma07_p04760.1 | chr07 | 3488716 | 3489276 | Ma07_g04760~ putative invertase inhibitor~ unknown_gene~missing_functional_completeness |
| invertase | Ma07_p24650.1 | chr07 | 31942909 | 31946255 | Ma07_g24650~ alkaline/neutral invertase CINV2-like, transcript variant X1~ unknown_gene~missing_functional_completeness |
| invertase | Ma07_p24650.2 | chr07 | 31942909 | 31946255 | Ma07_g24650~ alkaline/neutral invertase CINV2-like, transcript variant X1~ unknown_gene~missing_functional_completeness |
| invertase | Ma07_p24650.3 | chr07 | 31942909 | 31946255 | Ma07_g24650~ alkaline/neutral invertase CINV2-like, transcript variant X1~ un known_gene~missing_functional_completeness |
| invertase | Ma08_p06150.1 | chr08 | 4162979 | 4167866 | Ma08_g06150~ alkaline/neutral invertase CINV1-like, transcript variant X2~ unknown_gene~missing_functional_completeness |
| invertase | Ma08_p06150.2 | chr08 | 4162979 | 4167866 | Ma08_g06150~ alkaline/neutral invertase CINV1-like, transcript variant X2~ unknown_gene~missing_functional_completeness |
| invertase | Ma08_p15520.1 | chr08 | 15702459 | 15702959 | Ma08_g15520~ putative invertase inhibitor~ unknown_gene~missing_functional_completeness |
| invertase | Ma09_p08320.1 | chr09 | 5512236 | 5515812 | Ma09_g08320~ alkaline/neutral invertase CINV2-like, transcript variant X1~ unknown_gene~missing_functional_completeness |
| invertase | Ma09_p08320.2 | chr09 | 5512236 | 5515812 | Ma09_g08320~ alkaline/neutral invertase CINV2-like, transcript variant X1~ unknown_gene~missing_functional_completeness |
| invertase | Ma11_p00140.1 | chr11 | 51818 | 52333 | Ma11_g00140~ putative invertase inhibitor~ unknown_gene~missing_functional_completeness |
| invertase | Ma11_p00730.1 | chr11 | 525336 | 525899 | Ma11_g00730~ putative invertase inhibitor~ unknown_gene~missing_functional_completeness |

**Table S2**.*Cont*.

| Gene Name  Used in This  Research | Gene ID In  Banana Genome  Hub | Gene  Location | Start | End | Gene Annotation |
| --- | --- | --- | --- | --- | --- |
| invertase | Ma11_p15080.1 | chr11 | 20780848 | 20791224 | Ma11_g15080~ plant neutral invertase domain containing protein, expressed~ FBXO27~missing_completeness |
| alpha,alpha-trehalose-phosphate synthase | Ma08_p24250.1 | chr08 | 37500873 | 37503634 | Ma08_g24250~ probable alpha,alpha-trehalose-phosphate synthase [UDP-forming] 9~ unknown_gene~ missing_functional_completeness |
| alpha,alpha-trehalose-phosphate synthase | Ma08_p28360.1 | chr08 | 40314881 | 40318305 | Ma08_g28360~ probable alpha,alpha-trehalose-phosphate synthase [UDP-forming]  7~ unknown_gene~ missing_functional_completeness |
| alpha,alpha-trehalose-phosphate synthase | Ma09_p05470.1 | chr09 | 3517016 | 3520579 | Ma09_g05470~ probable alpha,alpha-trehalose-phosphate synthase [UDP-forming] 7, transcriptvariant X1~ unknown_gene~ missing_functional_completenes |
| alpha,alpha-trehalose-phosphate synthase | Ma09_p05470.2 | chr09 | 3517016 | 3520579 | Ma09_g05470~ probable alpha,alpha-trehalose-phosphate synthase [UDP-forming] 7, transcript variant X1~ unknown_gene~ missing_functional_completeness |
| alpha,alpha-trehalose-phosphate synthase | Ma09_p05570.1 | chr09 | 3577045 | 3595699 | Ma09_g05570~ alpha,alpha-trehalose-phosphate synthase [UDP-forming] 1~ unknown_gene~missing_functional_completeness |
| alpha,alpha-trehalose-phosphate synthase | Ma09_p15280.1 | chr09 | 10600787 | 10603526 | Ma09_g15280~ alpha,alpha-trehalose-phosphate synthase [UDP-forming] 6-like~ unknown_gene~ missing_functional_completeness |
| alpha,alpha-trehalose-phosphate synthase | Ma08_p28360.1 | chr08 | 40314881 | 40318305 | Ma08_g28360~ probable alpha,alpha-trehalose-phosphate synthase [UDP-forming]  7~ unknown_gene~ missing_functional_completeness |
| alpha,alpha-trehalose-phosphate synthase | Ma09_p05470.1 | chr09 | 3517016 | 3520579 | Ma09_g05470~ probable alpha,alpha-trehalose-phosphate synthase [UDP-forming] 7, transcriptvariant X1~ unknown_gene~ missing_functional_completenes |
| alpha,alpha-trehalose-phosphate synthase | Ma09_p05470.2 | chr09 | 3517016 | 3520579 | Ma09_g05470~ probable alpha,alpha-trehalose-phosphate synthase [UDP-forming] 7, transcript variant X1~ unknown_gene~ missing_functional_completeness |
| alpha,alpha-trehalose-phosphate synthase | Ma09_p05570.1 | chr09 | 3577045 | 3595699 | Ma09_g05570~ alpha,alpha-trehalose-phosphate synthase [UDP-forming] 1~ unknown_gene~missing_functional_completeness |
| alpha,alpha-trehalose-phosphate synthase | Ma09_p15280.1 | chr09 | 10600787 | 10603526 | Ma09_g15280~ alpha,alpha-trehalose-phosphate synthase [UDP-forming] 6-like~ unknown_gene~ missing_functional_completeness |
| alpha-amylase | Ma04_p21990.4 | chr04 | 24409413 | 24415152 | Ma04_g21990~ probable alpha-amylase 2, transcript variant X3~ unknown_gene~ missing_functional_completeness |
| alpha-amylase | Ma04_p22000.1 | chr04 | 24423034 | 24428870 | Ma04_g22000~ probable alpha-amylase 2~ unknown_gene~ missing_functional_completeness |
| alpha-amylase | Ma04_p22020.1 | chr04 | 24437598 | 24442436 | Ma04_g22020~ probable alpha-amylase 2, transcript variant X2~ unknown_gene~ missing_functional_completeness |
| alpha-amylase | Ma04_p22020.2 | chr04 | 24437598 | 24442436 | Ma04_g22020~ probable alpha-amylase 2, transcript variant X2~ unknown_gene~ missing_functional_completeness |
| alpha-amylase | Ma04_p22020.3 | chr04 | 24437598 | 24442436 | Ma04_g22020~ probable alpha-amylase 2, transcript variant X2~ unknown_gene~ missing_functional_completeness |
| alpha-amylase | Ma05_p08000.1 | chr05 | 5930054 | 5930738 | Ma05_g08000~ Alpha-amylase isozyme 3D~ AMY1.3~ fragment |
| alpha-amylase | Ma05_p08040.1 | chr05 | 5973638 | 5975129 | Ma05_g08040~ alpha-amylase isozyme 3C-like~ unknown_gene~ missing_functional_completeness |
| alpha-amylase | Ma05_p10310.1 | chr05 | 7435850 | 7437322 | Ma05_g10310~ Alpha-amylase isozyme 3C~ AMY1.2~ missing_completeness |
| alpha-amylase | Ma07_p20300.1 | chr07 | 28215978 | 28217929 | Ma07_g20300~ Alpha-amylase isozyme 3D~ AMY1.1~ complete |
| alpha-amylase | Ma08_p04100.1 | chr08 | 2873663 | 2886564 | Ma08_g04100~ alpha-amylase 3, chloroplastic~ unknown_gene~ missing_functional_completeness |

**Table S2**.*Cont*.

| Gene Name  Used in This  Research | Gene ID In  Banana Genome  Hub | Gene  Location | Start | End | Gene Annotation |
| --- | --- | --- | --- | --- | --- |
| alpha-amylase | Ma10_p30040.1 | chr10 | 36447425 | 36449250 | Ma10_g30040~ Alpha-amylase isozyme 3D~ AMY1.2~ missing_completeness |
| alpha-amylase | Ma04_p21990.1 | chr04 | 24409413 | 24415152 | Ma04_g21990~ probable alpha-amylase 2, transcript variant X3~ unknown_gene~ missing_functional_completeness |
| alpha-amylase | Ma04_p21990.2 | chr04 | 24409413 | 24415152 | Ma04_g21990~ probable alpha-amylase 2, transcript variant X3~ unknown_gene~ missing_functional_completeness |
| alpha-amylase | Ma04_p21990.3 | chr04 | 24409413 | 24415152 | Ma04_g21990~ probable alpha-amylase 2, transcript variant X3~ unknown_gene~ missing_functional_completeness |
| alpha-amylase | Ma04_p21990.3 | chr04 | 24409413 | 24415152 | Ma04_g21990~ probable alpha-amylase 2, transcript variant X3~ unknown_gene~ missing_functional_completeness |
| alpha-amylase | Ma04_p21990.2 | chr04 | 24409413 | 24415152 | Ma04_g21990~ probable alpha-amylase 2, transcript variant X3~ unknown_gene~ missing_functional_completeness |
| alpha-amylase | Ma04_p21990.1 | chr04 | 24409413 | 24415152 | Ma04_g21990~ probable alpha-amylase 2, transcript variant X3~ unknown_gene~ missing_functional_completeness |
| beta-amylase | Ma01_p10710.1 | chr01 | 7689326 | 7697895 | Ma01_g10710~ beta-amylase 2, chloroplastic-like, transcript variant X1~ unknown_gene~ missing_functional_completeness |
| alpha-glucosidase | [Ma01_p16300.1](http://banana-genome-hub.southgreen.fr/jbrowse_ma2/?loc=chr01:11805825..11814152) | chr01 | 11806825 | 11813152 | Ma01_g16300~ probable glucan 1,3-alpha-glucosidase~ unknown_gene~ missing_functional_completeness |
| alpha-glucosidase | [Ma03_p24590.1](http://banana-genome-hub.southgreen.fr/jbrowse_ma2/?loc=chr03:28885269..28890216) | chr03 | 28886269 | 28889216 | Ma03_g24590~ probable alpha-glucosidase Os06g0675700~ unknown_gene~ missing_functional_completeness |
| alpha-glucosidase | [Ma11_p06070.1](http://banana-genome-hub.southgreen.fr/jbrowse_ma2/?loc=chr11:4838812..4858808) | chr11 | 4839812 | 4857808 | Ma11_g06070~ neutral alpha-glucosidase C~ unknown_gene~missing_functional_ completeness |
| alpha-glucosidase | [Ma11_p24070.1](http://banana-genome-hub.southgreen.fr/jbrowse_ma2/?loc=chr11:27198106..27203152) | chr11 | 27199106 | 27202152 | Ma11_g24070~ Probable alpha-glucosidase Os06g0675700~ GAA~ complete |
| beta-amylase | Ma02_p15910.2 | chr02 | 23423787 | 23425928 | Ma02_g15910~ beta-amylase, transcript variant X2~ unknown_gene~ missing_functional_completeness |
| beta-amylase | Ma03_p08740.1 | chr03 | 6438399 | 6440252 | Ma03_g08740~ inactive beta-amylase 9-like~ unknown_gene~ missing_functional_completeness |
| beta-amylase | Ma03_p28390.1 | chr03 | 31507650 | 31514815 | Ma03_g28390~ beta-amylase 2, chloroplastic-like, transcript variant X2~ unknown_gene~ missing_functional_completeness |
| beta-amylase | Ma03_p28390.2 | chr03 | 31507650 | 31514815 | Ma03_g28390~ beta-amylase 2, chloroplastic-like, transcript variant X2~ unknown_gene~ missing_functional_completeness |
| beta-amylase | Ma04_p18390.1 | chr04 | 20279819 | 20283231 | Ma04_g18390~ beta-amylase 3, chloroplastic-like~ unknown_gene~ missing_functional_ completeness |
| beta-amylase | Ma05_p07800.1 | chr05 | 5704266 | 5706470 | Ma05_g07800~ inactive beta-amylase 9-like~ unknown_gene~ missing_functional_completeness |
| beta-amylase | Ma05_p09860.1 | chr05 | 7083186 | 7085680 | Ma05_g09860~ beta-amylase 1,chloroplastic~unknown_gene~missing_functional_completeness |
| beta-amylase | Ma06_p07470.1 | chr06 | 5301431 | 5307283 | Ma06_g07470~ beta-amylase 3, chloroplastic-like~unknown_gene~missing_functional_completeness |
| beta-amylase | Ma07_p10880.1 | chr07 | 8076577 | 8081499 | Ma07_g10880~ beta-amylase 3, chloroplastic-like~ unknown_gene~ missing_functional_completeness |
| beta-amylase | Ma08_p15460.1 | chr08 | 15535400 | 15537516 | Ma08_g15460~ beta-amylase 3, chloroplastic~ unknown_gene~ missing_functional_completeness |
| beta-amylase | Ma06_p25720.1 | chr06 | 26192247 | 26206326 | Ma06_g25720~ beta-amylase-like~ unknown_gene~ missing_functional_completeness |

**Table S2**.*Cont*.

| Gene Name  Used in This  Research | Gene ID In  Banana Genome  Hub | Gene  Location | Start | End | Gene Annotation |
| --- | --- | --- | --- | --- | --- |
| beta-fructofuranosidase | Ma04_p36400.1 | chr04 | 34796182 | 34799024 | Ma04_g36400~ Beta-fructofuranosidase, insoluble isoenzyme~ MACINV4~missing_completeness |
| beta-fructofuranosidase | Ma05_p22250.1 | chr05 | 33988495 | 33992359 | Ma05_g22250~ Beta-fructofuranosidase 1~ MAVIN2~ missing_completeness |
| beta-fructofuranosidase | Ma06_p14460.1 | chr06 | 9900683 | 9903068 | Ma06_g14460~ beta-fructofuranosidase, insoluble isoenzyme 3-like~ unknown_gene~ missing_functional_completeness |
| beta-fructofuranosidase | Ma06_p14700.1 | chr06 | 10051306 | 10054528 | Ma06_g14700~ Beta-fructofuranosidase, insoluble isoenzyme~ MACINV6~missing_completeness |
| beta-fructofuranosidase | Ma06_p14710.1 | chr06 | 10057144 | 10063200 | Ma06_g14710~ Beta-fructofuranosidase, insoluble isoenzyme~ MACINV7~missing_completeness |
| beta-fructofuranosidase | Ma10_p15780.1 | chr10 | 27750070 | 27751103 | Ma10_g15780~ Beta-fructofuranosidase,insoluble isoenzyme~MACINV1.1~ missing_completeness |
| beta-fructofuranosidase | Ma11_p20240.1 | chr11 | 24777565 | 24780421 | Ma11_g20240~ Beta-fructofuranosidase 1~ MAVIN1~ missing_completeness |
| beta-glucosidase | Ma02_p02580.1 | chr02 | 13812143 | 13816248 | Ma02_g02580~ beta-glucosidase 4-like~ unknown_gene~ missing_functional_completeness |
| beta-glucosidase | Ma02_p02590.1 | chr02 | 13818577 | 13830924 | Ma02_g02590~ beta-glucosidase 4-like~ unknown_gene~ missing_functional_completeness |
| beta-glucosidase | Ma02_p14610.1 | chr02 | 22609957 | 22611483 | Ma02_g14610~ glucan endo-1,3-beta-glucosidase 8-like~ unknown_gene~ missing_functional_completeness |
| beta-glucosidase | Ma02_p15740.1 | chr02 | 23320041 | 23323499 | Ma02_g15740~ glucan endo-1,3-beta-glucosidase 1-like~ unknown_gene~ missing_functional_completeness |
| beta-glucosidase | Ma02_p16750.1 | chr02 | 23966435 | 23968002 | Ma02_g16750~ glucan endo-1,3-beta-glucosidase 5-like~ unknown_gene~ missing_functional_completeness |
| beta-glucosidase | Ma02_p19490.1 | chr02 | 25639840 | 25643098 | Ma02_g19490~ glucan endo-1,3-beta-glucosidase 4-like, transcript variant X2~ unknown_gene~ missing_functional_completeness |
| beta-glucosidase | Ma02_p19490.2 | chr02 | 25639840 | 25644474 | Ma02_g19490~ glucan endo-1,3-beta-glucosidase 4-like, transcript variant X2~ unknown_gene~ missing_functional_completeness |
| beta-glucosidase | Ma02_p23470.1 | chr02 | 28449679 | 28453709 | Ma02_g23470~ glucan endo-1,3-beta-glucosidase 3-like~ unknown_gene~ missing_functional_completeness |
| beta-glucosidase | Ma03_p05470.1 | chr03 | 3584021 | 3588659 | Ma03_g05470~ glucan endo-1,3-beta-glucosidase 6-like, transcript variant X6~ unknown_gene~ missing_functional_completeness |
| beta-glucosidase | Ma03_p05470.2 | chr03 | 3584021 | 3588659 | Ma03_g05470~ glucan endo-1,3-beta-glucosidase 6-like, transcript variant X6~ unknown_gene~ missing_functional_completeness |
| beta-glucosidase | Ma03_p05470.3 | chr03 | 3584021 | 3588659 | Ma03_g05470~ glucan endo-1,3-beta-glucosidase 6-like, transcript variant X6~ unknown_gene~ missing_functional_completeness |
| beta-glucosidase | Ma03_p05470.4 | chr03 | 3584021 | 3588659 | Ma03_g05470~ glucan endo-1,3-beta-glucosidase 6-like, transcript variant X6~ unknown_gene~ missing_functional_completeness |
| beta-glucosidase | Ma03_p05470.6 | chr03 | 3584021 | 3588659 | Ma03_g05470~ glucan endo-1,3-beta-glucosidase 6-like, transcript variant X6~ unknown_gene~ missing_functional_completeness |
| beta-glucosidase | Ma03_p07920.1 | chr03 | 5673094 | 5674240 | Ma03_g07920~ glucan endo-1,3-beta-glucosidase-like~ unknown_gene~ missing_functional_completeness |
| beta-glucosidase | Ma03_p14720.1 | chr03 | 14518212 | 14519793 | Ma03_g14720~ glucan endo-1,3-beta-glucosidase~ unknown_gene~ missing_functional_completeness |
| beta-glucosidase | Ma03_p21750.1 | chr03 | 26667161 | 26678169 | Ma03_g21750~ beta-glucosidase 26-like~ unknown_gene~ missing_functional_completeness |
| beta-glucosidase | Ma03_p21770.1 | chr03 | 26699065 | 26701507 | Ma03_g21770~ beta-glucosidase 26-like~ unknown_gene~ missing_functional_completeness |

**Table S2**.*Cont*.

| Gene Name  Used in This  Research | Gene ID In  Banana Genome  Hub | Gene  Location | Start | End | Gene Annotation |
| --- | --- | --- | --- | --- | --- |
| beta-glucosidase | Ma03_p21870.1 | chr03 | 26762513 | 26762809 | Ma03_g21870~ Beta-glucosidase 26~ BGLU26~ fragment |
| beta-glucosidase | Ma03_p22600.1 | chr03 | 27331872 | 27350637 | Ma03_g22600~ beta-glucosidase 4, transcript variant X1~ unknown_gene~ missing_functional_completeness |
| beta-glucosidase | Ma03_p22600.2 | chr03 | 27331872 | 27350637 | Ma03_g22600~ beta-glucosidase 4, transcript variant X1~ unknown_gene~ missing_functional_completeness |
| beta-glucosidase | Ma03_p26780.1 | chr03 | 30378093 | 30379444 | Ma03_g26780~ glucan endo-1,3-beta-glucosidase 8~ unknown_gene~ missing_functional_completeness |
| beta-glucosidase | Ma03_p27680.1 | chr03 | 30972155 | 30973737 | Ma03_g27680~ glucan endo-1,3-beta-glucosidase 8-like~ unknown_gene~ missing_functional_completeness |
| beta-glucosidase | Ma03_p28110.1 | chr03 | 31268659 | 31269856 | Ma03_g28110~ glucan endo-1,3-beta-glucosidase-like~ unknown_gene~ missing_functional_completeness |
| beta-glucosidase | Ma03_p28760.1 | chr03 | 31855083 | 31857590 | Ma03_g28760~ glucan endo-1,3-beta-glucosidase 6-like~ unknown_gene~ missing_functional_completeness |
| beta-glucosidase | Ma03_p30760.1 | chr03 | 33252008 | 33254578 | Ma03_g30760~ beta-glucosidase 1-like~ unknown_gene~ missing_functional_completeness |
| beta-glucosidase | Ma03_p30770.1 | chr03 | 33257281 | 33258426 | Ma03_g30770~ glucan endo-1,3-beta-glucosidase 4-like, transcript variant X2~ unknown_gene~ missing_functional_completeness |
| beta-glucosidase | Ma03_p30770.2 | chr03 | 33257393 | 33258426 | Ma03_g30770~ glucan endo-1,3-beta-glucosidase 4-like, transcript variant X2~ unknown_gene~ missing_functional_completeness |
| beta-glucosidase | Ma03_p30770.3 | chr03 | 33257281 | 33258426 | Ma03_g30770~ glucan endo-1,3-beta-glucosidase 4-like, transcript variant X2~ unknown_gene~ missing_functional_completeness |
| beta-glucosidase | Ma03_p31730.1 | chr03 | 33835240 | 33836563 | Ma03_g31730~ glucan endo-1,3-beta-glucosidase 7-like~ unknown_gene~ missing_functional_completeness |
| beta-glucosidase | Ma04_p01380.1 | chr04 | 1257159 | 1260177 | Ma04_g01380~ beta-glucosidase 12-like~ unknown_gene~ missing_functional_completeness |
| beta-glucosidase | Ma04_p02670.1 | chr04 | 2228342 | 2231754 | Ma04_g02670~ glucan endo-1,3-beta-glucosidase 13, transcript variant X2~ unknown_gene~ missing_functional_completeness |
| beta-glucosidase | Ma04_p02670.2 | chr04 | 2228404 | 2231754 | Ma04_g02670~ glucan endo-1,3-beta-glucosidase 13, transcript variant X2~ unknown_gene~ missing_functional_completeness |
| beta-glucosidase | Ma04_p03310.1 | chr04 | 2564339 | 2565757 | Ma04_g03310~ glucan endo-1,3-beta-glucosidase 7-like, transcript variant X2~ unknown_gene~ missing_functional_completeness |
| beta-glucosidase | Ma04_p03310.2 | chr04 | 2564339 | 2565757 | Ma04_g03310~ glucan endo-1,3-beta-glucosidase 7-like, transcript variant X2~ unknown_gene~ missing_functional_completeness |
| beta-glucosidase | Ma04_p04770.1 | chr04 | 3641365 | 3642726 | Ma04_g04770~ glucan endo-1,3-beta-glucosidase 11-like~ unknown_gene~ missing_functional_completeness |
| beta-glucosidase | Ma04_p07940.1 | chr04 | 5739739 | 5742213 | Ma04_g07940~ glucan endo-1,3-beta-glucosidase 12-like~ unknown_gene~ missing_functional_completeness |
| beta-glucosidase | Ma04_p09280.1 | chr04 | 6625593 | 6628795 | Ma04_g09280~ glucan endo-1,3-beta-glucosidase 5-like~ unknown_gene~ missing_functional_completeness |
| beta-glucosidase | Ma04_p09620.1 | chr04 | 6819736 | 6822620 | Ma04_g09620~ glucan endo-1,3-beta-glucosidase 6-like, transcript variant X1~ unknown_gene~ missing_functional_completeness |

**Table S2.***Cont*.

| Gene Name  Used in This  Research | Gene ID In  Banana Genome  Hub | Gene  Location | Start | End | Gene Annotation |
| --- | --- | --- | --- | --- | --- |
| beta-glucosidase | Ma04_p09620.2 | chr04 | 6819736 | 6822620 | Ma04_g09620~ glucan endo-1,3-beta-glucosidase 6-like, transcript variant X1~ unknown_gene~ missing_functional_completeness |
| beta-glucosidase | Ma04_p14970.1 | chr04 | 11346166 | 11347691 | Ma04_g14970~ glucan endo-1,3-beta-glucosidase 3-like~ unknown_gene~ missing_functional_completeness |
| beta-glucosidase | Ma04_p32220.1 | chr04 | 32299849 | 32301393 | Ma04_g32220~ glucan endo-1,3-beta-glucosidase 11-like~ unknown_gene~ missing_functional_completeness |
| beta-glucosidase | Ma04_p34330.1 | chr04 | 33584678 | 33587492 | Ma04_g34330~ glucan endo-1,3-beta-glucosidase 1-like~ unknown_gene~ missing_functional_completeness |
| beta-glucosidase | Ma04_p34450.1 | chr04 | 33635287 | 33636418 | Ma04_g34450~ Putative Glucan endo-1,3-beta-glucosidase-like protein 2~ At5g61130~ fragment |
| beta-glucosidase | Ma04_p35340.1 | chr04 | 34160325 | 34162779 | Ma04_g35340~ beta-glucosidase 18-like~ unknown_gene~ missing_functional_completeness |
| beta-glucosidase | Ma04_p39890.1 | chr04 | 36876032 | 36879467 | Ma04_g39890~ glucan endo-1,3-beta-glucosidase 1-like~ unknown_gene~ missing_functional_completeness |
| beta-glucosidase | Ma05_p05730.1 | chr05 | 4351316 | 4358144 | Ma05_g05730~ glucan endo-1,3-beta-glucosidase 14-like, transcript variant X1~ unknown_gene~ missing_functional_completeness |
| beta-glucosidase | Ma05_p05730.2 | chr05 | 4351316 | 4358144 | Ma05_g05730~ glucan endo-1,3-beta-glucosidase 14-like, transcript variant X1~ unknown_gene~ missing_functional_completeness |
| beta-glucosidase | Ma05_p07840.1 | chr05 | 5759343 | 5760476 | Ma05_g07840~ putative glucan endo-1,3-beta-glucosidase GVI, transcript variant X2~ unknown_gene~ missing_functional_completeness |
| beta-glucosidase | Ma05_p07840.2 | chr05 | 5759343 | 5760476 | Ma05_g07840~ putative glucan endo-1,3-beta-glucosidase GVI, transcript variant X2~ unknown_gene~ missing_functional_completeness |
| beta-glucosidase | Ma05_p09640.1 | chr05 | 6962581 | 6963445 | Ma05_g09640~ glucan endo-1,3-beta-glucosidase 4-like~ unknown_gene~ missing_functional_completeness |
| beta-glucosidase | Ma05_p11130.1 | chr05 | 8070540 | 8071841 | Ma05_g11130~ glucan endo-1,3-beta-glucosidase 14~ unknown_gene~ missing_functional_completeness |
| beta-glucosidase | Ma05_p16710.1 | chr05 | 17994498 | 17995886 | Ma05_g16710~ glucan endo-1,3-beta-glucosidase 11-like~ unknown_gene~ missing_functional_completeness |
| beta-glucosidase | Ma05_p23890.1 | chr05 | 36054793 | 36060791 | Ma05_g23890~ beta-glucosidase 22-like, transcript variant X2~ unknown_gene~ missing_functional_completeness |
| beta-glucosidase | Ma05_p23890.2 | chr05 | 36054793 | 36060791 | Ma05_g23890~ beta-glucosidase 22-like, transcript variant X2~ unknown_gene~ missing_functional_completeness |
| beta-glucosidase | Ma05_p26340.1 | chr05 | 37998976 | 37999455 | Ma05_g26340~ putative glucan endo-1,3-beta-glucosidase GVI~ unknown_gene~ missing_functional_completeness |
| beta-glucosidase | Ma05_p27000.1 | chr05 | 38477629 | 38478437 | Ma05_g27000~ glucan endo-1,3-beta-glucosidase 13-like, transcript variant X2~ unknown_gene~ missing_functional_completeness |
| beta-glucosidase | Ma05_p27000.2 | chr05 | 38477629 | 38478408 | Ma05_g27000~ glucan endo-1,3-beta-glucosidase 13-like, transcript variant X2~ unknown_gene~ missing_functional_completeness |
| beta-glucosidase | Ma05_p27180.1 | chr05 | 38579240 | 38580820 | Ma05_g27180~ glucan endo-1,3-beta-glucosidase 8-like~ unknown_gene~ missing_functional_completeness |
| beta-glucosidase | Ma06_p00720.1 | chr06 | 560663 | 562356 | Ma06_g00720~ putative glucan endo-1,3-beta-glucosidase GVI, transcript variant X2~ unknown_gene~ missing_functional_completeness |

**Table S2**.*Cont*.

| Gene Name  Used in This  Research | Gene ID In  Banana Genome  Hub | Gene  Location | Start | End | Gene Annotation |
| --- | --- | --- | --- | --- | --- |
| beta-glucosidase | Ma06_p00720.2 | chr06 | 560663 | 562356 | Ma06_g00720~ putative glucan endo-1,3-beta-glucosidase GVI, transcript variant X2~ unknown_gene~ missing_functional_completeness |
| beta-glucosidase | Ma06_p06670.1 | chr06 | 4809618 | 4811111 | Ma06_g06670~ glucan endo-1,3-beta-glucosidase-like~ unknown_gene~ missing_functional_completeness |
| beta-glucosidase | Ma06_p13880.1 | chr06 | 9521864 | 9527096 | Ma06_g13880~ beta-glucosidase 12-like~ unknown_gene~ missing_functional_completeness |
| beta-glucosidase | Ma06_p14770.1 | chr06 | 10082544 | 10083821 | Ma06_g14770~ glucan endo-1,3-beta-glucosidase 14-like, transcript variant X1~ unknown_gene~ missing_functional_completeness |
| beta-glucosidase | Ma06_p14770.2 | chr06 | 10082544 | 10083821 | Ma06_g14770~ glucan endo-1,3-beta-glucosidase 14-like, transcript variant X1~ unknown_gene~ missing_functional_completeness |
| beta-glucosidase | Ma06_p29240.1 | chr06 | 30691869 | 30696886 | Ma06_g29240~ beta-glucosidase 6-like~ unknown_gene~ missing_functional_completeness |
| beta-glucosidase | Ma06_p29250.1 | chr06 | 30698494 | 30702133 | Ma06_g29250~ beta-glucosidase 6-like~ unknown_gene~ missing_functional_completeness |
| beta-glucosidase | Ma06_p31390.1 | chr06 | 32525333 | 32533425 | Ma06_g31390~ glucan endo-1,3-beta-glucosidase 4-like, transcript variant X1~ unknown_gene~ missing_functional_completeness |
| beta-glucosidase | Ma06_p31390.2 | chr06 | 32525333 | 32533425 | Ma06_g31390~ glucan endo-1,3-beta-glucosidase 4-like, transcript variant X1~ unknown_gene~ missing_functional_completeness |
| beta-glucosidase | Ma06_p31390.3 | chr06 | 32529318 | 32533425 | Ma06_g31390~ glucan endo-1,3-beta-glucosidase 4-like, transcript variant X1~ unknown_gene~ missing_functional_completeness |
| beta-glucosidase | Ma06_p31390.4 | chr06 | 32529318 | 32533425 | Ma06_g31390~ glucan endo-1,3-beta-glucosidase 4-like, transcript variant X1~ unknown_gene~ missing_functional_completeness |
| beta-glucosidase | Ma06_p31390.5 | chr06 | 32529318 | 32533425 | Ma06_g31390~ glucan endo-1,3-beta-glucosidase 4-like, transcript variant X1~ unknown_gene~ missing_functional_completeness |
| beta-glucosidase | Ma06_p31390.6 | chr06 | 32529318 | 32533425 | Ma06_g31390~ glucan endo-1,3-beta-glucosidase 4-like, transcript variant X1~ unknown_gene~ missing_functional_completeness |
| beta-glucosidase | Ma06_p31390.7 | chr06 | 32525333 | 32533358 | Ma06_g31390~ glucan endo-1,3-beta-glucosidase 4-like, transcript variant X1~ unknown_gene~ missing_functional_completeness |
| beta-glucosidase | Ma06_p33340.1 | chr06 | 34020538 | 34022502 | Ma06_g33340~ glucan endo-1,3-beta-glucosidase 7-like~ unknown_gene~ missing_functional_completeness |
| beta-glucosidase | Ma07_p06240.1 | chr07 | 4496227 | 4499341 | Ma07_g06240~ glucan endo-1,3-beta-glucosidase 5-like~ unknown_gene~ missing_functional_completeness |
| beta-glucosidase | Ma07_p06460.1 | chr07 | 4647579 | 4649126 | Ma07_g06460~ glucan endo-1,3-beta-glucosidase 8~ unknown_gene~ missing_functional_completeness |
| beta-glucosidase | Ma07_p08080.1 | chr07 | 6028643 | 6030512 | Ma07_g08080~ glucan endo-1,3-beta-glucosidase 7-like~ unknown_gene~ missing_functional_completeness |
| beta-glucosidase | Ma07_p12970.1 | chr07 | 9759286 | 9762444 | Ma07_g12970~ glucan endo-1,3-beta-glucosidase 14-like~ unknown_gene~ missing_functional_completeness |
| beta-glucosidase | Ma07_p20470.1 | chr07 | 28351832 | 28357336 | Ma07_g20470~ glucan 1,3-beta-glucosidase A-like, transcript variant X5~ unknown_gene~ missing_functional_completeness |
| beta-glucosidase | Ma07_p20470.2 | chr07 | 28351832 | 28357058 | Ma07_g20470~ glucan 1,3-beta-glucosidase A-like, transcript variant X5~ unknown_gene~ missing_functional_completeness |

**Table S2**.*Cont*.

| Gene Name  Used in This  Research | Gene ID In  Banana Genome  Hub | Gene  Location | Start | End | Gene Annotation |
| --- | --- | --- | --- | --- | --- |
| beta-glucosidase | Ma07_p20470.3 | chr07 | 28351832 | 28357058 | Ma07_g20470~ glucan 1,3-beta-glucosidase A-like, transcript variant X5~ unknown_gene~ missing_functional_completeness |
| beta-glucosidase | Ma07_p20470.4 | chr07 | 28351832 | 28357058 | Ma07_g20470~ glucan 1,3-beta-glucosidase A-like, transcript variant X5~ unknown_gene~ missing_functional_completeness |
| beta-glucosidase | Ma07_p20470.5 | chr07 | 28351832 | 28357058 | Ma07_g20470~ glucan 1,3-beta-glucosidase A-like, transcript variant X5~ unknown_gene~ missing_functional_completeness |
| beta-glucosidase | Ma07_p20470.6 | chr07 | 28352633 | 28357336 | Ma07_g20470~ glucan 1,3-beta-glucosidase A-like, transcript variant X5~ unknown_gene~ missing_functional_completeness |
| beta-glucosidase | Ma07_p20470.7 | chr07 | 28351832 | 28357058 | Ma07_g20470~ glucan 1,3-beta-glucosidase A-like, transcript variant X5~ unknown_gene~ missing_functional_completeness |
| beta-glucosidase | Ma07_p22060.1 | chr07 | 30132894 | 30135096 | Ma07_g22060~ beta-glucosidase 24-like, transcript variant X1~ unknown_gene~ missing_functional_completeness |
| beta-glucosidase | Ma07_p22060.2 | chr07 | 30132894 | 30136171 | Ma07_g22060~ beta-glucosidase 24-like, transcript variant X1~ unknown_gene~ missing_functional_completeness |
| beta-glucosidase | Ma07_p22060.3 | chr07 | 30132894 | 30136171 | Ma07_g22060~ beta-glucosidase 24-like, transcript variant X1~ unknown_gene~ missing_functional_completeness |
| beta-glucosidase | Ma07_p28350.1 | chr07 | 34430789 | 34432101 | Ma07_g28350~ glucan endo-1,3-beta-glucosidase 7-like~ unknown_gene~ missing_functional_completeness |
| beta-glucosidase | Ma08_p04500.1 | chr08 | 3125402 | 3133696 | Ma08_g04500~ glucan endo-1,3-beta-glucosidase 8-like~ unknown_gene~ missing_functional_completeness |
| beta-glucosidase | Ma08_p08530.1 | chr08 | 5999326 | 6000859 | Ma08_g08530~ probable glucan endo-1,3-beta-glucosidase A6, transcript variant X1~ unknown_gene~ missing_functional_completeness |
| beta-glucosidase | Ma08_p08530.2 | chr08 | 5999187 | 6000859 | Ma08_g08530~ probable glucan endo-1,3-beta-glucosidase A6, transcript variant X1~ unknown_gene~ missing_functional_completeness |
| beta-glucosidase | Ma08_p15020.1 | chr08 | 14988716 | 14994591 | Ma08_g15020~ beta-glucosidase 18-like, transcript variant X1~ unknown_gene~ missing_functional_completeness |
| beta-glucosidase | Ma08_p15020.2 | chr08 | 14988716 | 14994591 | Ma08_g15020~ beta-glucosidase 18-like, transcript variant X1~ unknown_gene~ missing_functional_completeness |
| beta-glucosidase | Ma08_p20040.1 | chr08 | 33891394 | 33893988 | Ma08_g20040~ glucan endo-1,3-beta-glucosidase 4-like, transcript variant X1~ unknown_gene~ missing_functional_completeness |
| beta-glucosidase | Ma08_p23140.1 | chr08 | 36559173 | 36560272 | Ma08_g23140~ glucan endo-1,3-beta-glucosidase-like~ unknown_gene~ missing_functional_completeness |
| beta-glucosidase | Ma08_p23150.1 | chr08 | 36567538 | 36569826 | Ma08_g23150~ glucan endo-1,3-beta-glucosidase-like~ unknown_gene~ missing_functional_completeness |
| beta-glucosidase | Ma08_p23160.1 | chr08 | 36570764 | 36571867 | Ma08_g23160~ glucan endo-1,3-beta-glucosidase-like~ unknown_gene~ missing_functional_completeness |
| beta-glucosidase | Ma08_p23270.1 | chr08 | 36643929 | 36645834 | Ma08_g23270~ glucan endo-1,3-beta-glucosidase 10-like~ unknown_gene~ missing_functional_completeness |
| beta-glucosidase | Ma08_p25110.1 | chr08 | 38078316 | 38082227 | Ma08_g25110~ beta-glucosidase 24-like~ unknown_gene~ missing_functional_completeness |

**Table S2**.*Cont*.

| Gene Name  Used in This  Research | Gene ID In  Banana Genome  Hub | Gene  Location | Start | End | Gene Annotation |
| --- | --- | --- | --- | --- | --- |
| beta-glucosidase | Ma08_p25420.1 | chr08 | 38263637 | 38265816 | Ma08_g25420~ Glucan endo-1,3-beta-glucosidase 6~ At5g58090~ fragment |
| beta-glucosidase | Ma08_p33490.1 | chr08 | 43776193 | 43780299 | Ma08_g33490~ beta-glucosidase 25-like~ unknown_gene~ missing_functional_completeness |
| beta-glucosidase | Ma09_p00800.1 | chr09 | 544595 | 546367 | Ma09_g00800~ glucan endo-1,3-beta-glucosidase-like~ unknown_gene~ missing_functional_completeness |
| beta-glucosidase | Ma09_p06900.1 | chr09 | 4391138 | 4396236 | Ma09_g06900~ beta-glucosidase 6-like~ unknown_gene~ missing_functional_completeness |
| beta-glucosidase | Ma09_p07510.1 | chr09 | 4932369 | 4938337 | Ma09_g07510~ beta-glucosidase 13-like~ unknown_gene~ missing_functional_completeness |
| beta-glucosidase | Ma09_p08880.1 | chr09 | 5908023 | 5909127 | Ma09_g08880~ glucan endo-1,3-beta-glucosidase 1~ unknown_gene~ missing_functional_completeness |
| beta-glucosidase | Ma09_p12070.1 | chr09 | 8167038 | 8169953 | Ma09_g12070~ beta-glucosidase 12-like~ unknown_gene~ missing_functional_completeness |
| beta-glucosidase | Ma09_p15550.1 | chr09 | 10886165 | 10887931 | Ma09_g15550~ glucan endo-1,3-beta-glucosidase 11-like~ unknown_gene~ missing_functional_completeness |
| beta-glucosidase | Ma09_p15750.1 | chr09 | 11079092 | 11082405 | Ma09_g15750~ beta-glucosidase 22-like~ unknown_gene~ missing_functional_completeness |
| beta-glucosidase | Ma09_p15780.1 | chr09 | 11114219 | 11119097 | Ma09_g15780~ beta-glucosidase 22-like~ unknown_gene~ missing_functional_completeness |
| beta-glucosidase | Ma09_p23580.1 | chr09 | 35414373 | 35415923 | Ma09_g23580~ glucan endo-1,3-beta-glucosidase 6-like~ unknown_gene~ missing_functional_completeness |
| beta-glucosidase | Ma09_p26260.1 | chr09 | 37569105 | 37572154 | Ma09_g26260~ glucan endo-1,3-beta-glucosidase 8-like~ unknown_gene~ missing_functional_completeness |
| beta-glucosidase | Ma10_p00120.1 | chr10 | 1347091 | 1349143 | Ma10_g00120~ glucan endo-1,3-beta-glucosidase 14-like, transcript variantX1~ unknown_gene~ missing_functional_completeness |
| beta-glucosidase | Ma10_p00120.2 | chr10 | 1347091 | 1348198 | Ma10_g00120~ glucan endo-1,3-beta-glucosidase 14-like, transcript variant X1~ unknown_gene~ missing_functional_completeness |
| beta-glucosidase | Ma10_p05560.1 | chr10 | 16909591 | 16911905 | Ma10_g05560~ Glucan endo-1,3-beta-glucosidase~ At1g32860~ complete |
| beta-glucosidase | Ma10_p11590.1 | chr10 | 25055300 | 25056809 | Ma10_g11590~ glucan endo-1,3-beta-glucosidase 7~ unknown_gene~ missing_functional_completeness |
| beta-glucosidase | Ma10_p13270.1 | chr10 | 26129934 | 26133381 | Ma10_g13270~ glucan endo-1,3-beta-glucosidase 9-like~ unknown_gene~ missing_functional_completeness |
| beta-glucosidase | Ma10_p14500.1 | chr10 | 26926014 | 26927600 | Ma10_g14500~ glucan endo-1,3-beta-glucosidase 11-like~ unknown_gene~ missing_functional_completeness |
| beta-glucosidase | Ma10_p15690.1 | chr10 | 27706284 | 27708294 | Ma10_g15690~ glucan endo-1,3-beta-glucosidase 14-like, transcript variant X1~ unknown_gene~ missing_functional_completeness |
| beta-glucosidase | Ma10_p15690.2 | chr10 | 27706284 | 27708294 | Ma10_g15690~ glucan endo-1,3-beta-glucosidase 14-like, transcript variant X1~ unknown_gene~ missing_functional_completeness |
| beta-glucosidase | Ma10_p21680.1 | chr10 | 31365622 | 31367573 | Ma10_g21680~ glucan endo-1,3-beta-glucosidase 14-like, transcript variant X1~ unknown_gene~ missing_functional_completeness |
| beta-glucosidase | Ma10_p21680.2 | chr10 | 31365812 | 31367573 | Ma10_g21680~ glucan endo-1,3-beta-glucosidase 14-like, transcript variant X1~ unknown_gene~ missing_functional_completeness |
| beta-glucosidase | Ma10_p21680.3 | chr10 | 31365735 | 31367573 | Ma10_g21680~ glucan endo-1,3-beta-glucosidase 14-like, transcript variant X1~ unknown_gene~ missing_functional_completeness |

**Table S2**.*Cont*.

| Gene Name  Used in This  Research | Gene ID In  Banana Genome  Hub | Gene  Location | Start | End | Gene Annotation |
| --- | --- | --- | --- | --- | --- |
| beta-glucosidase | Ma10_p25200.1 | chr10 | 33465794 | 33486353 | Ma10_g25200~ glucan endo-1,3-beta-glucosidase 7-like~ unknown_gene~ missing_functional_completeness |
| beta-glucosidase | Ma10_p28720.1 | chr10 | 35511136 | 35512694 | Ma10_g28720~ glucan endo-1,3-beta-glucosidase 12-like~ unknown_gene~ missing_functional_completeness |
| beta-glucosidase | Ma11_p00930.1 | chr11 | 674437 | 675825 | Ma11_g00930~ glucan endo-1,3-beta-glucosidase 10-like~ unknown_gene~ missing_functional_completeness |
| beta-glucosidase | Ma11_p05350.1 | chr11 | 4094196 | 4106732 | Ma11_g05350~ beta-glucosidase 22-like~ unknown_gene~ missing_functional_completeness |
| beta-glucosidase | Ma11_p05360.1 | chr11 | 4098728 | 4099445 | Ma11_g05360~ beta-glucosidase 22-like~ unknown_gene~ missing_functional_completeness |
| beta-glucosidase | Ma11_p06280.1 | chr11 | 5075789 | 5081133 | Ma11_g06280~ beta-glucosidase 18-like~ unknown_gene~ missing_functional_completeness |
| beta-glucosidase | Ma11_p06420.1 | chr11 | 5174637 | 5177449 | Ma11_g06420~ beta-glucosidase 32-like, transcript variant X1~ unknown_gene~ missing_functional_completeness |
| beta-glucosidase | Ma11_p06420.2 | chr11 | 5174637 | 5177449 | Ma11_g06420~ beta-glucosidase 32-like, transcript variant X1~ unknown_gene~ missing_functional_completeness |
| beta-glucosidase | Ma11_p06460.1 | chr11 | 5270328 | 5270989 | Ma11_g06460~ glucan endo-1,3-beta-glucosidase 1-like~ unknown_gene~ missing_functional_completeness |
| beta-glucosidase | Ma11_p09090.1 | chr11 | 7257836 | 7262029 | Ma11_g09090~ glucan endo-1,3-beta-glucosidase 3-like~ unknown_gene~ missing_functional_completeness |
| beta-glucosidase | Ma11_p12600.1 | chr11 | 16490299 | 16493098 | Ma11_g12600~ glucan endo-1,3-beta-glucosidase 3-like~ unknown_gene~ missing_functional_completeness |
| beta-glucosidase | Ma11_p13910.1 | chr11 | 19070734 | 19073503 | Ma11_g13910~ probable glucan endo-1,3-beta-glucosidase A6, transcript variant X1~ unknown_gene~ missing_functional_completeness |
| beta-glucosidase | Ma11_p13910.2 | chr11 | 19070734 | 19073503 | Ma11_g13910~ probable glucan endo-1,3-beta-glucosidase A6, transcript variant X1~ unknown_gene~ missing_functional_completeness |
| beta-glucosidase | Ma11_p20910.1 | chr11 | 25274750 | 25276083 | Ma11_g20910~ glucan endo-1,3-beta-glucosidase 10-like, transcript variant X2~ unknown_gene~ missing_functional_completeness |
| beta-glucosidase | Ma11_p20910.2 | chr11 | 25274750 | 25276083 | Ma11_g20910~ glucan endo-1,3-beta-glucosidase 10-like, transcript variant X2~ unknown_gene~ missing_functional_completeness |
| beta-glucosidase | Ma11_p22700.1 | chr11 | 26383910 | 26385706 | Ma11_g22700~ glucan endo-1,3-beta-glucosidase-like~ unknown_gene~ missing_functional_completeness |
| beta-glucosidase | Ma11_p24540.1 | chr11 | 27474240 | 27476957 | Ma11_g24540~ glucan endo-1,3-beta-glucosidase 14-like, transcript variant X1~ unknown_gene~ missing_functional_completeness |
| beta-glucosidase | Ma10_p15800.1 | chr10 | 27768148 | 27769178 | Ma10_g15800~ Beta-fructofuranosidase, insoluble isoenzyme~ MACINV1.2~missing_completeness |
| fructokinase | Ma01_p05870.1 | chr01 | 4152379 | 4163421 | Ma01_g05870~ 6-phosphofructokinase 5, chloroplastic~ pfp~ complete |
| fructokinase | Ma02_p02410.1 | chr02 | 13652692 | 13654157 | Ma02_g02410~ fructokinase-1-like~ unknown_gene~ missing_functional_completeness |
| fructokinase | Ma02_p18360.1 | chr02 | 24871930 | 24876386 | Ma02_g18360~ 6-phosphofructokinase 3~ pfp~ complete |
| fructokinase | Ma03_p22670.1 | chr03 | 27426336 | 27427703 | Ma03_g22670~ fructokinase-2~ unknown_gene~ missing_functional_completeness |
| fructokinase | Ma04_p01000.1 | chr04 | 885610 | 892035 | Ma04_g01000~ 6-phosphofructokinase 3~ pfp~ complete |

**Table S2**.*Cont*.

| Gene Name  Used in This  Research | Gene ID In  Banana Genome  Hub | Gene  Location | Start | End | Gene Annotation |
| --- | --- | --- | --- | --- | --- |
| fructokinase | Ma04_p08280.1 | chr04 | 5948415 | 5954645 | Ma04_g08280~ 6-phosphofructokinase 3~ pfp~ complete |
| fructokinase | Ma04_p14670.1 | chr04 | 11117212 | 11120851 | Ma04_g14670~ fructokinase-2-like, transcript variant X2~ unknown_gene~ missing_functional_completeness |
| fructokinase | Ma04_p14670.2 | chr04 | 11117212 | 11120851 | Ma04_g14670~ fructokinase-2-like, transcript variant X2~ unknown_gene~ missing_functional_completeness |
| fructokinase | Ma04_p15430.1 | chr04 | 11709406 | 11713186 | Ma04_g15430~ 6-phosphofructokinase 3~ pfp~ pseudogene |
| fructokinase | Ma04_p16060.1 | chr04 | 14703159 | 14704473 | Ma04_g16060~ fructokinase-1-like~ unknown_gene~ missing_functional_completeness |
| fructokinase | Ma04_p24700.1 | chr04 | 26656659 | 26662692 | Ma04_g24700~ 6-phosphofructokinase 3~ pfp~ complete |
| fructokinase | Ma04_p25170.1 | chr04 | 26976696 | 26985497 | Ma04_g25170~ fructokinase-1~ unknown_gene~ missing_functional_completeness |
| fructokinase | Ma05_p05600.1 | chr05 | 4249350 | 4255738 | Ma05_g05600~ fructokinase-1-like~ unknown_gene~ missing_functional_completeness |
| fructokinase | Ma05_p24880.1 | chr05 | 37017753 | 37020935 | Ma05_g24880~ 6-phosphofructokinase 2~ pfkA1~ complete |
| fructokinase | Ma06_p01570.1 | chr06 | 1282563 | 1284132 | Ma06_g01570~ fructokinase-1-like, transcript variant X2~ unknown_gene~ missing_functional_completeness |
| fructokinase | Ma06_p01570.2 | chr06 | 1282563 | 1284132 | Ma06_g01570~ fructokinase-1-like, transcript variant X2~ unknown_gene~ missing_functional_completeness |
| fructokinase | Ma06_p13970.1 | chr06 | 9571759 | 9572972 | Ma06_g13970~ fructokinase-1-like~ unknown_gene~ missing_functional_completeness |
| fructokinase | Ma06_p19740.1 | chr06 | 13830476 | 13835302 | Ma06_g19740~ fructokinase-2-like~ unknown_gene~ missing_functional_completeness |
| fructokinase | Ma07_p10560.1 | chr07 | 7890072 | 7894434 | Ma07_g10560~ 6-phosphofructokinase 3~ pfkA~ pseudogene |
| fructokinase | Ma07_p11340.1 | chr07 | 8423633 | 8430537 | Ma07_g11340~ 6-phosphofructokinase 5, chloroplastic~ pfkA2~ complete |
| fructokinase | Ma07_p13520.1 | chr07 | 10169690 | 10173615 | Ma07_g13520~ 6-phosphofructokinase 3~ pfp~ complete |
| fructokinase | Ma09_p18430.1 | chr09 | 17917457 | 17918920 | Ma09_g18430~ 6-phosphofructokinase 2~ pfp~ complete |
| fructokinase | Ma10_p13930.1 | chr10 | 26535733 | 26537427 | Ma10_g13930~ fructokinase-1-like~ unknown_gene~ missing_functional_completeness |
| fructokinase | Ma10_p16460.1 | chr10 | 28151870 | 28153162 | Ma10_g16460~ probable fructokinase-1~ unknown_gene~ missing_functional_completeness |
| fructokinase | Ma11_p08450.1 | chr11 | 6731755 | 6733030 | Ma11_g08450~ fructokinase-1-like~ unknown_gene~ missing_functional_completeness |
| fructokinase | Ma11_p17680.1 | chr11 | 22937810 | 22939030 | Ma11_g17680~ fructokinase-1-like~ unknown_gene~ missing_functional_completeness |
| glucan phosphorylase | Ma04_p39130.1 | chr04 | 36333919 | 36341460 | Ma04_g39130~ alpha-1,4 glucan phosphorylase L isozyme, chloroplastic/amyloplastic~ unknown_gene~ missing_functional_completeness |
| glucan phosphorylase | Ma06_p35790.1 | chr06 | 35532228 | 35539621 | Ma06_g35790~ alpha-glucan phosphorylase, H isozyme~ unknown_gene~ missing_functional_completenes |
| glucose-1-phosphate adenylyltransferase | Ma01_p00130.1 | chr01 | 117249 | 122508 | Ma01_g00130~ glucose-1-phosphate adenylyltransferase small subunit,chloroplastic/amyloplastic~unknown_gene~missing_functional_completeness |
| glucose-1-phosphate adenylyltransferase | Ma01_p05380.1 | chr01 | 3785658 | 3799336 | Ma01_g05380~ glucose-1-phosphate adenylyltransferase large subunit 3,chloroplastic/amyloplastic~unknown_gene~ missing_functional_completeness |
| glucose-1-phosphate adenylyltransferase | Ma03_p22640.1 | chr03 | 27387485 | 27390075 | Ma03_g22640~ glucose-1-phosphate adenylyltransferase small subunit, chloroplastic-like~ unknown_gene~missing_functional_completeness |
| glucose-1-phosphate adenylyltransferase | Ma04_p02930.1 | chr04 | 2364543 | 2370015 | Ma04_g02930~ glucose-1-phosphate adenylyltransferase large subunit 1-like~ unknown_gene~  missing_functional_completeness |

**Table S2**.*Cont*.

| Gene Name  Used in This  Research | Gene ID In  Banana Genome  Hub | Gene  Location | Start | End | Gene Annotation |
| --- | --- | --- | --- | --- | --- |
| glucose-1-phosphate adenylyltransferase | Ma04_p10600.1 | chr04 | 7534659 | 7539106 | Ma04_g10600~ glucose-1-phosphate adenylyltransferase large subunit 1-like~ unknown_gene~  missing_functional_completeness |
| glucose-1-phosphate adenylyltransferase | Ma06_p17480.1 | chr06 | 11872689 | 11876113 | Ma06_g17480~ glucose-1-phosphate adenylyltransferase large subunit, putative,expressed~ TC_0679~ fragment |
| glucose-1-phosphate adenylyltransferase | Ma06_p28940.1 | chr06 | 30492186 | 30495094 | Ma06_g28940~ glucose-1-phosphate adenylyltransferase large subunit 1, transcript variant X1~ unknown_gene~missing_functional_completeness |
| glucose-1-phosphate adenylyltransferase | Ma06_p28940.2 | chr06 | 30492186 | 30495094 | Ma06_g28940~ glucose-1-phosphate adenylyltransferase large subunit 1, transcript variant X1~ unknown_gene~missing_functional_completeness |
| glucose-1-phosphate adenylyltransferase | Ma09_p06650.1 | chr09 | 4254772 | 4259173 | Ma09_g06650~ glucose-1-phosphate adenylyltransferase large subunit1-like~ unknown_gene~  missing_functional_completeness |
| glucose-6-phosphate  isomerase | Ma07_p08070.1 | chr07 | 6016867 | 6025844 | Ma07_g08070~ glucose-6-phosphate isomerase, cytosolic, transcript variant X1~ nknown_gene~ missing_functional_Completeness |
| glucose-6-phosphate isomerase | Ma09_p14520.1 | chr09 | 9911884 | 9922066 | Ma09_g14520~glucose-6-phosphate isomerase1,chloroplastic-like~unknown_gene~missing_functional_completeness |
| glucose-6-phosphate isomerase | Ma10_p24940.1 | chr10 | 33300445 | 33308162 | Ma10_g24940~glucose-6-phosphate isomerase 1, chloroplastic-like~unknown_gene~ missing_functional_completeness |
| glucose-6-phosphate isomerase | Ma10_p28640.1 | chr10 | 35455241 | 35470300 | Ma10_g28640~ glucose-6-phosphate isomerase, cytosolic 2B-like~unknown_gene~ missing_functional_completeness |
| pectinesterase | Ma01_p07260.1 | chr01 | 5247736 | 5249274 | Ma01_g07260~ probable pectinesterase 53~ unknown_gene~ missing_functional_completeness |
| pectinesterase | Ma01_p13770.1 | chr01 | 10084235 | 10086198 | Ma01_g13770~ putative pectinesterase 10~ unknown_gene~ missing_functional_completeness |
| pectinesterase | Ma02_p01680.1 | chr02 | 10810328 | 10812110 | Ma02_g01680~ putative pectinesterase/pectinesterase inhibitor 28~ unknown_gene~ missing_functional_completeness |
| pectinesterase | Ma02_p06020.1 | chr02 | 17105514 | 17107232 | Ma02_g06020~ putative pectinesterase 14~ unknown_gene~ missing_functional_completeness |
| pectinesterase | Ma02_p20500.1 | chr02 | 26466652 | 26468005 | Ma02_g20500~ probable pectinesterase 67~ unknown_gene~ missing_functional_completeness |
| pectinesterase | Ma02_p21670.1 | chr02 | 27228384 | 27230254 | Ma02_g21670~ probable pectinesterase/pectinesterase inhibitor 34~ unknown_gene~ missing_functional_completeness |
| pectinesterase | Ma03_p05660.1 | chr03 | 3884878 | 3886525 | Ma03_g05660~ pectinesterase-like~ unknown_gene~ missing_functional_completeness |
| pectinesterase | Ma03_p05670.1 | chr03 | 3890642 | 3892718 | Ma03_g05670~ probable pectinesterase/pectinesterase inhibitor 12~ unknown_gene~ missing_functional_completeness |
| pectinesterase | Ma03_p05680.1 | chr03 | 3893202 | 3895295 | Ma03_g05680~ pectinesterase-like~ unknown_gene~ missing_functional_completeness |
| pectinesterase | Ma03_p05690.1 | chr03 | 3897191 | 3899042 | Ma03_g05690~ pectinesterase 3~ unknown_gene~ missing_functional_completeness |
| pectinesterase | Ma03_p32650.1 | chr03 | 34369577 | 34370524 | Ma03_g32650~ Putative Probable pectinesterase/pectinesterase inhibitor 51~ PME51~ fragment |
| pectinesterase | Ma04_p20040.1 | chr04 | 22784960 | 22786638 | Ma04_g20040~ pectinesterase-like~ unknown_gene~ missing_functional_completeness |
| pectinesterase | Ma04_p20250.1 | chr04 | 22981012 | 22982442 | Ma04_g20250~ probable pectinesterase 68~ unknown_gene~ missing_functional_completeness |
| pectinesterase | Ma04_p30590.1 | chr04 | 31210429 | 31212126 | Ma04_g30590~ probable pectinesterase/pectinesterase inhibitor 46~ unknown_gene~missing_functional_completeness |
| pectinesterase | Ma04_p33470.1 | chr04 | 33069792 | 33071416 | Ma04_g33470~ probable pectinesterase 68~ unknown_gene~ missing_functional_completeness |
| pectinesterase | Ma04_p40060.1 | chr04 | 36981551 | 36983755 | Ma04_g40060~ pectinesterase-like~ unknown_gene~ missing_functional_completeness |

**Table S2**.*Cont*.

| Gene Name  Used in This  Research | Gene ID In  Banana Genome  Hub | Gene  Location | Start | End | Gene Annotation |
| --- | --- | --- | --- | --- | --- |
| pectinesterase | Ma05_p01200.1 | chr05 | 721988 | 723855 | Ma05_g01200~ probable pectinesterase/pectinesterase inhibitor 51~ unknown_gene~ missing_functional_completeness |
| pectinesterase | Ma05_p07660.1 | chr05 | 5565752 | 5567539 | Ma05_g07660~ probable pectinesterase 29~ unknown_gene~ missing_functional_completeness |
| pectinesterase | Ma05_p13530.1 | chr05 | 9812367 | 9814040 | Ma05_g13530~ pectinesterase-like~ unknown_gene~ missing_functional_completeness |
| pectinesterase | Ma05_p20500.1 | chr05 | 32172210 | 32172713 | Ma05_g20500~ pectinesterase inhibitor-like~ unknown_gene~ missing_functional_completeness |
| pectinesterase | Ma05_p23700.1 | chr05 | 35848754 | 35850514 | Ma05_g23700~ putative pectinesterase/pectinesterase inhibitor 28~ unknown_gene~ missing_functional_completeness |
| pectinesterase | Ma05_p23770.1 | chr05 | 35912756 | 35913427 | Ma05_g23770~ Pectinesterase~ PECS-2.1~ fragment |
| pectinesterase | Ma05_p23780.1 | chr05 | 35916985 | 35918043 | Ma05_g23780~ pectinesterase 3-like~ unknown_gene~ missing_functional_completeness |
| pectinesterase | Ma05_p24470.1 | chr05 | 36716828 | 36719120 | Ma05_g24470~ putative pectinesterase 63~ unknown_gene~ missing_functional_completeness |
| pectinesterase | Ma05_p26410.1 | chr05 | 38032410 | 38034514 | Ma05_g26410~ pectinesterase/pectinesterase inhibitor PPE8B-like~ unknown_gene~ missing_functional_completeness |
| pectinesterase | Ma06_p12310.1 | chr06 | 8552217 | 8554950 | Ma06_g12310~ putative pectinesterase/pectinesterase inhibitor 45~ unknown_gene~ missing_functional_completeness |
| pectinesterase | Ma06_p27730.1 | chr06 | 29620226 | 29620330 | Ma06_g27730~ Probable pectinesterase/pectinesterase inhibitor 51~ PME51~ fragment |
| pectinesterase | Ma06_p28140.1 | chr06 | 29903768 | 29906395 | Ma06_g28140~ probable pectinesterase 53, transcript variant X2~ unknown_gene~ missing_functional_completeness |
| pectinesterase | Ma06_p28140.2 | chr06 | 29904413 | 29906395 | Ma06_g28140~ probable pectinesterase 53, transcript variant X2~ unknown_gene~ missing_functional_completeness |
| pectinesterase | Ma06_p28140.3 | chr06 | 29903768 | 29906395 | Ma06_g28140~ probable pectinesterase 53, transcript variant X2~ unknown_gene~ missing_functional_completeness |
| pectinesterase | Ma06_p28980.1 | chr06 | 30512471 | 30513935 | Ma06_g28980~ putative pectinesterase 11~ unknown_gene~ missing_functional_completeness |
| pectinesterase | Ma06_p30950.1 | chr06 | 32184182 | 32185864 | Ma06_g30950~ probable pectinesterase/pectinesterase inhibitor 51~  unknown_gene~ missing_functional_completeness |
| pectinesterase | Ma06_p37410.1 | chr06 | 36438373 | 36440290 | Ma06_g37410~ pectinesterase-like~ unknown_gene~ missing_functional_completeness |
| pectinesterase | Ma06_p37420.1 | chr06 | 36457172 | 36464428 | Ma06_g37420~ putative pectinesterase/pectinesterase inhibitor 28~ unknown_gene~ missing_functional_completeness |
| pectinesterase | Ma07_p11280.1 | chr07 | 8397950 | 8400299 | Ma07_g11280~ pectinesterase-like~ unknown_gene~ missing_functional_completeness |
| pectinesterase | Ma07_p12250.1 | chr07 | 9161561 | 9163349 | Ma07_g12250~ pectinesterase-like~ unknown_gene~ missing_functional_completeness |
| pectinesterase | Ma07_p12260.1 | chr07 | 9174098 | 9176079 | Ma07_g12260~ pectinesterase-like~ unknown_gene~ missing_functional_completeness |
| pectinesterase | Ma07_p15530.1 | chr07 | 11684722 | 11687076 | Ma07_g15530~ pectinesterase/pectinesterase inhibitor PPE8B-like~ unknown_gene~ missing_functional_completeness |
| pectinesterase | Ma07_p28300.1 | chr07 | 34399503 | 34400959 | Ma07_g28300~ pectinesterase QRT1~ unknown_gene~ missing_functional_completeness |
| pectinesterase | Ma08_p10100.1 | chr08 | 7363347 | 7365148 | Ma08_g10100~ pectinesterase-like~ unknown_gene~ missing_functional_completeness |
| pectinesterase | Ma08_p10420.1 | chr08 | 7623625 | 7625475 | Ma08_g10420~ putative pectinesterase 10~ unknown_gene~ missing_functional_completeness |
| pectinesterase | Ma08_p10430.1 | chr08 | 7630961 | 7631812 | Ma08_g10430~ probable pectinesterase 66~ unknown_gene~ missing_functional_completeness |
| pectinesterase | Ma08_p10440.1 | chr08 | 7633099 | 7637947 | Ma08_g10440~ putative pectinesterase 10~ unknown_gene~ missing_functional_completeness |
| pectinesterase | Ma08_p10460.1 | chr08 | 7644451 | 7646295 | Ma08_g10460~ putative pectinesterase 10~ unknown_gene~ missing_functional_completeness |

**Table S2**.*Cont*.

| Gene Name  Used in This  Research | Gene ID  In Banana  Genome Hub | Gene  Location | Start | End | Gene Annotation |
| --- | --- | --- | --- | --- | --- |
| pectinesterase | Ma08_p23010.1 | chr08 | 36477866 | 36478540 | Ma08_g23010~ pectinesterase inhibitor-like~ unknown_gene~ missing_functional_completeness |
| pectinesterase | Ma08_p23170.1 | chr08 | 36573309 | 36575198 | Ma08_g23170~ putative pectinesterase/pectinesterase inhibitor 22~ unknown_gene~ missing_functional_completeness |
| pectinesterase | Ma08_p24480.1 | chr08 | 37637351 | 37639132 | Ma08_g24480~ pectinesterase-like~ unknown_gene~ missing_functional_completeness |
| pectinesterase | Ma08_p24490.1 | chr08 | 37642286 | 37642837 | Ma08_g24490~ pectinesterase inhibitor-like~ unknown_gene~ missing_functional_completeness |
| pectinesterase | Ma08_p24500.1 | chr08 | 37646153 | 37648166 | Ma08_g24500~ probable pectinesterase/pectinesterase inhibitor 25, transcript variant X2~unknown_gene~missing_functional_completeness |
| pectinesterase | Ma08_p24500.2 | chr08 | 37646153 | 37648166 | Ma08_g24500~ probable pectinesterase/pectinesterase inhibitor 25, transcript variant X2~ unknown_gene~missing_functional_completeness |
| pectinesterase | Ma08_p24510.1 | chr08 | 37648562 | 37650539 | Ma08_g24510~ probable pectinesterase/pectinesterase inhibitor 58~ unknown_gene~ missing_functional_Completeness |
| pectinesterase | Ma08_p24520.1 | chr08 | 37655856 | 37657811 | Ma08_g24520~ pectinesterase-like~ unknown_gene~ missing_functional_completeness |
| pectinesterase | Ma08_p30380.1 | chr08 | 41665824 | 41667098 | Ma08_g30380~ probable pectinesterase 66~ unknown_gene~ missing_functional_completeness |
| pectinesterase | Ma08_p34870.1 | chr08 | 44869358 | 44871562 | Ma08_g34870~ probable pectinesterase 53, transcript variant X2~ unknown_gene~ missing_functional_completeness |
| pectinesterase | Ma08_p34870.2 | chr08 | 44869358 | 44871562 | Ma08_g34870~ probable pectinesterase 53, transcript variant X2~ unknown_gene~ missing_functional_completeness |
| pectinesterase | Ma08_p34870.3 | chr08 | 44869358 | 44871562 | Ma08_g34870~ probable pectinesterase 53, transcript variant X2~ unknown_gene~ missing_functional_completeness |
| pectinesterase | Ma09_p06050.1 | chr09 | 3906182 | 3906328 | Ma09_g06050~ Probable pectinesterase/pectinesterase inhibitor 51~ PME51~ fragment |
| pectinesterase | Ma09_p13670.1 | chr09 | 9264626 | 9266730 | Ma09_g13670~ probable pectinesterase/pectinesterase inhibitor 12~ unknown_gene~ missing_functional_completeness |
| pectinesterase | Ma09_p13680.1 | chr09 | 9267076 | 9269022 | Ma09_g13680~ pectinesterase-like~ unknown_gene~ missing_functional_completeness |
| pectinesterase | Ma09_p13690.1 | chr09 | 9269656 | 9269901 | Ma09_g13690~ pectinesterase 3-like~ unknown_gene~ missing_functional_completeness |
| pectinesterase | Ma09_p13700.1 | chr09 | 9277059 | 9279053 | Ma09_g13700~ probable pectinesterase/pectinesterase inhibitor 16~ unknown_gene~ missing_functional_completeness |
| pectinesterase | Ma09_p28080.1 | chr09 | 38921999 | 38923779 | Ma09_g28080~ pectinesterase-like~ unknown_gene~ missing_functional_completeness |
| pectinesterase | Ma09_p30710.1 | chr09 | 40700227 | 40700760 | Ma09_g30710~ pectinesterase 1-like~ unknown_gene~ missing_functional_completeness |
| pectinesterase | Ma10_p02970.1 | chr10 | 10151971 | 10154101 | Ma10_g02970~ pectinesterase-like~ unknown_gene~ missing_functional_completeness |
| pectinesterase | Ma10_p02990.1 | chr10 | 10310576 | 10311163 | Ma10_g02990~ pectinesterase inhibitor-like~ unknown_gene~ missing_functional_completeness |
| pectinesterase | Ma10_p04350.1 | chr10 | 15035641 | 15037842 | Ma10_g04350~ probable pectinesterase/pectinesterase inhibitor 51~ unknown_gene~ missing_functional_completeness |
| pectinesterase | Ma10_p15140.1 | chr10 | 27363170 | 27365036 | Ma10_g15140~ Probable pectinesterase/pectinesterase inhibitor 34~ PME2~ complete |
| pectinesterase | Ma11_p04760.1 | chr11 | 3694531 | 3695352 | Ma11_g04760~ Putative pectinesterase 10~ PME10~ fragment |
| pectinesterase | Ma11_p05180.1 | chr11 | 3992443 | 3994228 | Ma11_g05180~ pectinesterase-like~ unknown_gene~ missing_functional_completeness |
| pectinesterase | Ma11_p05190.1 | chr11 | 3995625 | 3998518 | Ma11_g05190~ pectinesterase-like~ unknown_gene~ missing_functional_completeness |
| pectinesterase | Ma11_p11240.1 | chr11 | 12691305 | 12704353 | Ma11_g11240~ pectinesterase 31~ unknown_gene~ missing_functional_completeness |
| pectinesterase | Ma11_p14380.1 | chr11 | 19679851 | 19683335 | Ma11_g14380~ pectinesterase-like~ unknown_gene~ missing_functional_completeness |

**Table S2**.*Cont*.

| Gene Name  Used in This  Research | Gene ID  In Banana  Genome Hub | Gene  Location | Start | End | Gene Annotation |
| --- | --- | --- | --- | --- | --- |
| pectinesterase | Ma11_p14580.1 | chr11 | 20310559 | 20311907 | Ma11_g14580~ putative pectinesterase 10~ unknown_gene~ missing_functional_completeness |
| pectinesterase | Ma11_p18960.1 | chr11 | 23953694 | 23955499 | Ma11_g18960~ pectinesterase-like~ unknown_gene~ missing_functional_completeness |
| pectinesterase | Ma11_p19000.1 | chr11 | 23973316 | 23975063 | Ma11_g19000~ putative pectinesterase/pectinesterase inhibitor 28~ unknown_gene~ missing_functional_completeness |
| pectinesterase | Ma11_p23740.1 | chr11 | 26982541 | 26984350 | Ma11_g23740~ putative pectinesterase/pectinesterase inhibitor 28~ unknown_gene~ missing_functional_completeness |
| phosphoglucomutase | Ma02_p08630.1 | chr02 | 18912151 | 18920306 | Ma02_g08630~ phosphoglucomutase, cytoplasmic 2-like~ unknown_gene~ missing_functional_completeness |
| phosphoglucomutase | Ma08_p15070.1 | chr08 | 15015087 | 15022457 | Ma08_g15070~ phosphoglucomutase, chloroplastic~ unknown_gene~ missing_functional_completeness |
| phosphoglucomutase | Ma08_p31340.1 | chr08 | 42285583 | 42294687 | Ma08_g31340~ phosphoglucomutase, cytoplasmic 2~ unknown_gene~ missing_functional_completeness |
| polygalacturonase | Ma01_p06340.1 | chr01 | 4521997 | 4524438 | Ma01_g06340~ polygalacturonase At1g48100~ unknown_gene~missing_functional_completeness |
| polygalacturonase | Ma01_p09800.1 | chr01 | 7033940 | 7035021 | Ma01_g09800~ exopolygalacturonase-like~ unknown_gene~ missing_functional_completeness |
| polygalacturonase | Ma01_p09810.1 | chr01 | 7039841 | 7040651 | Ma01_g09810~ polygalacturonase-like~ unknown_gene~ missing_functional_completeness |
| polygalacturonase | Ma01_p12940.1 | chr01 | 9469476 | 9471666 | Ma01_g12940~ exopolygalacturonase-like~ unknown_gene~ missing_functional_completeness |
| polygalacturonase | Ma01_p16370.1 | chr01 | 11861778 | 11864169 | Ma01_g16370~ polygalacturonase-like~ unknown_gene~ missing_functional_completeness |
| polygalacturonase | Ma01_p17690.1 | chr01 | 12976122 | 12977642 | Ma01_g17690~ exopolygalacturonase-like~ unknown_gene~ missing_functional_completeness |
| polygalacturonase | Ma01_p21350.1 | chr01 | 20983314 | 20986768 | Ma01_g21350~ Putative Polygalacturonase At1g48100~ ADPG1~ complete |
| polygalacturonase | Ma01_p22170.1 | chr01 | 23826585 | 23827030 | Ma01_g22170~ Putative Exopolygalacturonase~ PG2C~ fragment |
| polygalacturonase | Ma02_p01230.1 | chr02 | 8885497 | 8920990 | Ma02_g01230~ exopolygalacturonase-like~ unknown_gene~ missing_functional_completeness |
| polygalacturonase | Ma02_p03440.1 | chr02 | 14781968 | 14783516 | Ma02_g03440~ polygalacturonase QRT3~ unknown_gene~ missing_functional_completeness |
| polygalacturonase | Ma02_p03760.1 | chr02 | 15174619 | 15179079 | Ma02_g03760~ probable polygalacturonase~ unknown_gene~ missing_functional_completeness |
| polygalacturonase | Ma02_p04450.1 | chr02 | 15882268 | 15884071 | Ma02_g04450~ polygalacturonase QRT3~ unknown_gene~ missing_functional_completeness |
| polygalacturonase | Ma02_p09960.1 | chr02 | 19703091 | 19705072 | Ma02_g09960~ probable polygalacturonase~ unknown_gene~ missing_functional_completeness |
| polygalacturonase | Ma02_p16140.1 | chr02 | 23615704 | 23617633 | Ma02_g16140~ polygalacturonase At1g48100-like~ unknown_gene~ missing_functional_completeness |
| polygalacturonase | Ma03_p08330.1 | chr03 | 6009445 | 6011414 | Ma03_g08330~ polygalacturonase At1g48100-like~ unknown_gene~ missing_functional_completeness |
| polygalacturonase | Ma03_p09990.1 | chr03 | 7443668 | 7448354 | Ma03_g09990~ probable polygalacturonase~ unknown_gene~ missing_functional_completeness |
| polygalacturonase | Ma03_p10820.1 | chr03 | 8176605 | 8178416 | Ma03_g10820~ polygalacturonase ADPG2~ unknown_gene~ missing_functional_completeness |
| polygalacturonase | Ma03_p15740.1 | chr03 | 17014633 | 17017951 | Ma03_g15740~ probable polygalacturonase At1g80170, transcript variant X1~ unknown_gene~ missing_functional_Completeness |
| polygalacturonase | Ma03_p15740.2 | chr03 | 17014633 | 17017951 | Ma03_g15740~ probable polygalacturonase At1g80170, transcript variant X1~ unknown_gene~ missing_functional_Completeness |
| polygalacturonase | Ma03_p28500.1 | chr03 | 31649863 | 31650507 | Ma03_g28500~ probable polygalacturonase~ unknown_gene~ missing_functional_completeness |
| polygalacturonase | Ma03_p28510.1 | chr03 | 31651337 | 31652330 | Ma03_g28510~ probable polygalacturonase~ unknown_gene~ missing_functional_completeness |
| polygalacturonase | Ma04_p02960.1 | chr04 | 2379433 | 2381513 | Ma04_g02960~ Putative Polygalacturonase At1g48100~ JNA2~ complete |

**Table S2**.*Cont*.

| Gene Name  Used in This  Research | Gene ID  In Banana  Genome Hub | Gene  Location | Start | End | Gene Annotation |
| --- | --- | --- | --- | --- | --- |
| polygalacturonase | Ma04_p05610.1 | chr04 | 4187957 | 4190512 | Ma04_g05610~ probable polygalacturonase, transcript variant X2~ unknown_gene~ missing_functional_completeness |
| polygalacturonase | Ma04_p05610.2 | chr04 | 4187957 | 4190512 | Ma04_g05610~ probable polygalacturonase, transcript variant X2~ unknown_gene~ missing_functional_completeness |
| polygalacturonase | Ma04_p06360.1 | chr04 | 4682711 | 4686229 | Ma04_g06360~ probable polygalacturonase At1g80170~ unknown_gene~ missing_functional_completeness |
| polygalacturonase | Ma04_p06370.1 | chr04 | 4687087 | 4692330 | Ma04_g06370~ polygalacturonase ADPG2-like~ unknown_gene~ missing_functional_completeness |
| polygalacturonase | Ma04_p08510.1 | chr04 | 6078581 | 6083422 | Ma04_g08510~ probable polygalacturonase~ unknown_gene~ missing_functional_completeness |
| polygalacturonase | Ma04_p13200.1 | chr04 | 9975303 | 9978003 | Ma04_g13200~ polygalacturonase ADPG1-like~ unknown_gene~ missing_functional_completeness |
| polygalacturonase | Ma04_p15690.1 | chr04 | 12020766 | 12024043 | Ma04_g15690~ probable polygalacturonase~ unknown_gene~ missing_functional_completeness |
| polygalacturonase | Ma04_p25920.1 | chr04 | 27550752 | 27552447 | Ma04_g25920~ exopolygalacturonase-like~ unknown_gene~ missing_functional_completeness |
| polygalacturonase | Ma04_p28680.1 | chr04 | 29691749 | 29696657 | Ma04_g28680~ probable polygalacturonase At1g80170~ unknown_gene~ missing_functional_completeness |
| polygalacturonase | Ma04_p31040.1 | chr04 | 31477424 | 31479351 | Ma04_g31040~ polygalacturonase At1g48100-like~ unknown_gene~ missing_functional_completeness |
| polygalacturonase | Ma05_p00110.1 | chr05 | 75223 | 79124 | Ma05_g00110~ probable polygalacturonase, transcript variant X1~ unknown_gene~ missing_functional_completeness |
| polygalacturonase | Ma05_p00110.2 | chr05 | 75223 | 79124 | Ma05_g00110~ probable polygalacturonase, transcript variant X1~ unknown_gene~ missing_functional_completeness |
| polygalacturonase | Ma05_p03980.1 | chr05 | 2980915 | 2997183 | Ma05_g03980~ exopolygalacturonase-like~ unknown_gene~ missing_functional_completeness |
| polygalacturonase | Ma05_p08780.1 | chr05 | 6465834 | 6468048 | Ma05_g08780~ Putative Polygalacturonase At1g48100~ JNA2~ complete |
| polygalacturonase | Ma05_p24510.1 | chr05 | 36736808 | 36741620 | Ma05_g24510~ probable polygalacturonase~ unknown_gene~ missing_functional_completeness |
| polygalacturonase | Ma06_p01640.1 | chr06 | 1336374 | 1337774 | Ma06_g01640~ polygalacturonase-like~ unknown_gene~ missing_functional_completeness |
| polygalacturonase | Ma06_p15750.1 | chr06 | 10661039 | 10664363 | Ma06_g15750~ probable polygalacturonase, transcript variant X1~ unknown_gene~ missing_functional_completeness |
| polygalacturonase | Ma06_p15750.2 | chr06 | 10661039 | 10664363 | Ma06_g15750~ probable polygalacturonase, transcript variant X1~ unknown_gene~ missing_functional_completeness |
| polygalacturonase | Ma06_p19580.1 | chr06 | 13500671 | 13502081 | Ma06_g19580~ polygalacturonase-like~ unknown_gene~ missing_functional_completeness |
| polygalacturonase | Ma06_p23580.1 | chr06 | 21659559 | 21663168 | Ma06_g23580~ Putative Polygalacturonase At1g48100~ QRT2~ complete |
| polygalacturonase | Ma06_p24560.1 | chr06 | 23103936 | 23104922 | Ma06_g24560~ polygalacturonase inhibitor 1-like~ unknown_gene~ missing_functional_completeness |
| polygalacturonase | Ma07_p01880.1 | chr07 | 1436403 | 1441226 | Ma07_g01880~ probable polygalacturonase At1g80170, transcript variant X2~ unknown_gene~ missing_functional_Completeness |
| polygalacturonase | Ma07_p01880.2 | chr07 | 1436403 | 1441226 | Ma07_g01880~ probable polygalacturonase At1g80170, transcript variant X2~ unknown_gene~ missing_functional_Completeness |
| polygalacturonase | Ma07_p16630.1 | chr07 | 15127383 | 15128879 | Ma07_g16630~ exopolygalacturonase-like~ unknown_gene~ missing_functional_completeness |

**Table S2**.*Cont*.

| Gene Name  Used in This  Research | Gene ID  In Banana  Genome Hub | Gene  Location | Start | End | Gene Annotation |
| --- | --- | --- | --- | --- | --- |
| polygalacturonase | Ma07_p25980.1 | chr07 | 32959879 | 32961226 | Ma07_g25980~ Putative Probable polygalacturonase~ GSVIVT00026920001~ fragment |
| polygalacturonase | Ma08_p06040.1 | chr08 | 4087649 | 4091179 | Ma08_g06040~ probable polygalacturonase~ unknown_gene~ missing_functional_completeness |
| polygalacturonase | Ma08_p06080.1 | chr08 | 4114576 | 4116373 | Ma08_g06080~ polygalacturonase At1g48100-like~ unknown_gene~ missing_functional_completeness |
| polygalacturonase | Ma08_p21130.1 | chr08 | 35114780 | 35117214 | Ma08_g21130~ probable polygalacturonase, transcript variant X1~ unknown_gene~ missing_functional_completeness |
| polygalacturonase | Ma08_p21130.2 | chr08 | 35114780 | 35117214 | Ma08_g21130~ probable polygalacturonase, transcript variant X1~ unknown_gene~ missing_functional_completeness |
| polygalacturonase | Ma08_p22940.1 | chr08 | 36419342 | 36421153 | Ma08_g22940~ polygalacturonase At1g48100-like, transcript variant X1~ unknown_gene~ missing_functional_Completeness |
| polygalacturonase | Ma08_p22940.2 | chr08 | 36419347 | 36421153 | Ma08_g22940~ polygalacturonase At1g48100-like, transcript variant X1~ unknown_gene~ missing_functional_Completeness |
| polygalacturonase | Ma08_p24340.1 | chr08 | 37560827 | 37565193 | Ma08_g24340~ probable polygalacturonase~ unknown_gene~ missing_functional_completeness |
| polygalacturonase | Ma08_p25100.1 | chr08 | 38076085 | 38077020 | Ma08_g25100~ polygalacturonase inhibitor-like~ unknown_gene~ missing_functional_completeness |
| polygalacturonase | Ma09_p02430.1 | chr09 | 1715931 | 1717354 | Ma09_g02430~ polygalacturonase-like~ unknown_gene~ missing_functional_completeness |
| polygalacturonase | Ma09_p02440.1 | chr09 | 1717731 | 1718897 | Ma09_g02440~ polygalacturonase-like~ unknown_gene~ missing_functional_completeness |
| polygalacturonase | Ma09_p06190.1 | chr09 | 3977785 | 3978119 | Ma09_g06190~ polygalacturonase, putative, expressed~ unknown_gene~ fragment |
| polygalacturonase | Ma09_p12060.1 | chr09 | 8156469 | 8157455 | Ma09_g12060~ polygalacturonase inhibitor-like~ unknown_gene~ missing_functional_completeness |
| polygalacturonase | Ma09_p15970.1 | chr09 | 11323418 | 11325379 | Ma09_g15970~ polygalacturonase At1g48100-like~ unknown_gene~ missing_functional_completeness |
| polygalacturonase | Ma09_p17590.1 | chr09 | 13168137 | 13172543 | Ma09_g17590~ probable polygalacturonase~ unknown_gene~ missing_functional_completeness |
| polygalacturonase | Ma09_p22850.1 | chr09 | 34721849 | 34730620 | Ma09_g22850~ polygalacturonase ADPG1-like~ unknown_gene~ missing_functional_completeness |
| polygalacturonase | Ma09_p28250.1 | chr09 | 39079595 | 39081234 | Ma09_g28250~ exopolygalacturonase-like~ unknown_gene~ missing_functional_completeness |
| polygalacturonase | Ma10_p04110.1 | chr10 | 14760541 | 14760974 | Ma10_g04110~ polygalacturonase-like~ unknown_gene~ missing_functional_completeness |
| polygalacturonase | Ma10_p09950.1 | chr10 | 24065021 | 24067994 | Ma10_g09950~ polygalacturonase-like~ unknown_gene~ missing_functional_completeness |
| polygalacturonase | Ma10_p09960.1 | chr10 | 24072197 | 24075826 | Ma10_g09960~ polygalacturonase-like~ unknown_gene~ missing_functional_completeness |
| polygalacturonase | Ma10_p21800.1 | chr10 | 31424344 | 31426618 | Ma10_g21800~ probable polygalacturonase~ unknown_gene~ missing_functional_completeness |
| polygalacturonase | Ma10_p28490.1 | chr10 | 35349899 | 35369984 | Ma10_g28490~ probable polygalacturonase At3g15720~ unknown_gene~ missing_functional_completeness |
| polygalacturonase | Ma10_p28500.1 | chr10 | 35372730 | 35373661 | Ma10_g28500~ polygalacturonase ADPG1-like~ unknown_gene~ missing_functional_completeness |
| polygalacturonase | Ma11_p02770.1 | chr11 | 2025842 | 2037114 | Ma11_g02770~ probable polygalacturonase, transcript variant X1~ unknown_gene~ missing_functional_completeness |
| polygalacturonase | Ma11_p02770.2 | chr11 | 2025842 | 2069313 | Ma11_g02770~ probable polygalacturonase, transcript variant X1~ unknown_gene~ missing_functional_completeness |

**Table S2**.*Cont*.

| Gene Name  Used in This  Research | Gene ID  In Banana  Genome Hub | Gene  Location | Start | End | Gene Annotation |
| --- | --- | --- | --- | --- | --- |
| polygalacturonase | Ma11_p02780.1 | chr11 | 2027929 | 2029485 | Ma11_g02780~ probable polygalacturonase~ unknown_gene~ missing_functional_completeness |
| polygalacturonase | Ma11_p04650.1 | chr11 | 3626911 | 3628781 | Ma11_g04650~ polygalacturonase At1g48100~ unknown_gene~ missing_functional_completeness |
| polygalacturonase | Ma11_p08890.1 | chr11 | 7039835 | 7043213 | Ma11_g08890~ Putative Probable polygalacturonase~ ADPG1~ complete |
| polygalacturonase | Ma11_p12450.1 | chr11 | 16408528 | 16411919 | Ma11_g12450~ probable polygalacturonase~ unknown_gene~ missing_functional_completeness |
| polygalacturonase | Ma11_p12720.1 | chr11 | 16631892 | 16635134 | Ma11_g12720~ probable polygalacturonase~ unknown_gene~ missing_functional_completeness |
| polygalacturonase | Ma11_p15340.1 | chr11 | 21019430 | 21024051 | Ma11_g15340~ polygalacturonase QRT2-like~ unknown_gene~ missing_functional_completeness |
| polygalacturonase | Ma11_p16570.1 | chr11 | 22064992 | 22068008 | Ma11_g16570~ probable polygalacturonase~ unknown_gene~ missing_functional_completeness |
| polygalacturonate4-alpha-galacturonosyltransferase | Ma07_p18890.1 | chr07 | 26829897 | 26835326 | Ma07_g18890~ polygalacturonate 4-alpha-galacturonosyltransferase-like~ unknown_gene~missing_functional_completeness |
| Polygalacturonate4-alpha-galacturonosyltransferase | Ma09_p24740.1 | chr09 | 36359778 | 36366613 | Ma09_g24740~ polygalacturonate 4-alpha-galacturonosyltransferase-like, transcript  variant X2~ unknown_gene~ missing_functional_completeness |
| Polygalacturonate 4-alpha-  galacturonosyltransferase | Ma09_p24740.2 | chr09 | 36359778 | 36366613 | Ma09_g24740~ polygalacturonate 4-alpha-galacturonosyltransferase-like, transcript variant X2~unknown_gene~ missing_functional_Completeness |
| starch synthase | Ma01_p03500.1 | chr01 | 2315559 | 2319734 | Ma01_g03500~ granule-bound starch synthase 1, chloroplastic/amyloplastic-like~  unknown_gene~ missing_functional_completeness |
| starch synthase | Ma01_p09900.1 | chr01 | 7112269 | 7113901 | Ma01_g09900~ Granule-bound starch synthase 1, chloroplastic/amyloplastic~ WAXY~ fragment |
| starch synthase | Ma01_p09910.1 | chr01 | 7113976 | 7115127 | Ma01_g09910~ Granule-bound starch synthase 1, chloroplastic/amyloplastic~ WAXY~ fragment |
| starch synthase | Ma01_p17750.1 | chr01 | 13011043 | 13020134 | Ma01_g17750~ Granule-bound starch synthase 2, chloroplastic/amyloplastic~ SSII-2~ missing_completeness |
| starch synthase | Ma01_p17760.1 | chr01 | 13020177 | 13021906 | Ma01_g17760~ Putative Granule-bound starch synthase 2, chloroplastic/amyloplastic~ SS2~ fragment |
| starch synthase | Ma03_p03110.1 | chr03 | 2099013 | 2110334 | Ma03_g03110~ soluble starch synthase 1, chloroplastic/amyloplastic, transcript variant X1~  unknown_gene~ missing_functional_completeness |
| starch synthase | Ma03_p03110.2 | chr03 | 2099013 | 2110334 | Ma03_g03110~ soluble starch synthase 1, chloroplastic/amyloplastic, transcript variant X1~  unknown_gene~ missing_functional_completeness |
| starch synthase | Ma03_p16630.1 | chr03 | 19223227 | 19239353 | Ma03_g16630~ probable starch synthase 4, chloroplastic/amyloplastic, transcript variant X1~  unknown_gene~ missing_functional_completeness |
| starch synthase | Ma03_p16630.2 | chr03 | 19221054 | 19239353 | Ma03_g16630~ probable starch synthase 4, chloroplastic/amyloplastic, transcript variant X1~  unknown_gene~ missing_functional_completeness |
| starch synthase | Ma04_p10170.1 | chr04 | 7272430 | 7278033 | Ma04_g10170~ Granule-bound starch synthase 2, chloroplastic/amyloplastic~ SSII-2~ missing_completeness |
| starch synthase | Ma04_p25840.1 | chr04 | 27471598 | 27475417 | Ma04_g25840~ granule-bound starch synthase 1, chloroplastic/amyloplastic-like~ unknown_  gene~ missing_functional_completeness |
| starch synthase | Ma05_p00630.1 | chr05 | 350325 | 370981 | Ma05_g00630~ starch synthase 3, chloroplastic/amyloplastic-like~ unknown_gene~ missing_functional_completeness |
| starch synthase | Ma06_p25980.1 | chr06 | 26590301 | 26595478 | Ma06_g25980~ Soluble starch synthase 2-1, chloroplastic/amyloplastic~ SSII-2~ missing_completeness |
| starch synthase | Ma08_p24830.1 | chr08 | 37903440 | 37904345 | Ma08_g24830~ Granule-bound starch synthase 2, chloroplastic/amyloplastic~ SSII-2~ missing_completeness |

**Table S2.***Cont*.

| Gene Name  Used in This  Research | Gene ID  In Banana  Genome Hub | Gene  Location | Start | End | Gene Annotation |
| --- | --- | --- | --- | --- | --- |
| starch synthase | Ma08_p28160.1 | chr08 | 40195725 | 40207318 | Ma08_g28160~ probable starch synthase 4, chloroplastic/amyloplastic, transcript variant X1~  unknown_gene~ missing_functional_completeness |
| starch synthase | Ma08_p28160.2 | chr08 | 40195725 | 40207318 | Ma08_g28160~ probable starch synthase 4, chloroplastic/amyloplastic, transcript variant X1~  unknown_gene~ missing_functional_completeness |
| starch synthase | Ma09_p21400.1 | chr09 | 32393292 | 32396211 | Ma09_g21400~ granule-bound starch synthase 1, chloroplastic/amyloplastic-like~ unknown_gene~missing_functional_completens |
| starch synthase | Ma11_p17030.1 | chr11 | 22423709 | 22426932 | Ma11_g17030~ Soluble starch synthase 3, chloroplastic/amyloplastic~ glgA2~ missing_completeness |
| starch synthase | Ma11_p17040.1 | chr11 | 22428748 | 22444376 | Ma11_g17040~ Soluble starch synthase 3, chloroplastic/amyloplastic~ glgA2~missing_completeness |
| sucrose-phosphate  synthase | Ma04_p06090.1 | chr04 | 4497097 | 4505492 | Ma04_g06090~Sucrose-phosphate synthase~ SPS~ missing_completeness |
| sucrose-phosphate  synthase | Ma04_p16190.1 | chr04 | 14918502 | 14925734 | Ma04_g16190~ Sucrose-phosphate synthase~ SPS~ missing_completeness |
| sucrose-phosphate  synthase | Ma06_p18890.1 | chr06 | 12915073 | 12919344 | Ma06_g18890~ Sucrose-phosphate synthase~ SPS~ missing_completeness |
| sucrose-phosphate  synthase | Ma09_p23020.1 | chr09 | 34924004 | 34936459 | Ma09_g23020~ Sucrose-phosphate synthase~ SPS~ missing_completeness |
| sucrose synthase | Ma02_p23730.1 | chr02 | 28634738 | 28638803 | Ma02_g23730~ sucrose synthase 2-like~ unknown_gene~ missing_functional_completeness |
| sucrose synthase | Ma03_p01820.1 | chr03 | 1238804 | 1243225 | Ma03_g01820~ sucrose synthase 2-like~ unknown_gene~ missing_functional_completeness |
| sucrose synthase | Ma03_p08100.1 | chr03 | 5838112 | 5846436 | Ma03_g08100~ Sucrose synthase 2~ unknown_gene~ pseudogene |
| sucrose synthase | Ma03_p15100.1 | chr03 | 14864201 | 14867914 | Ma03_g15100~ sucrose synthase 7-like~ unknown_gene~ missing_functional_completeness |
| sucrose synthase | Ma06_p12350.1 | chr06 | 8581522 | 8585693 | Ma06_g12350~ sucrose synthase 2, transcript variant X2~ unknown_gene~ missing_functional_completeness |
| sucrose synthase | Ma06_p12350.2 | chr06 | 8581522 | 8585693 | Ma06_g12350~ sucrose synthase 2, transcript variant X2~ unknown_gene~ missing_functional_completeness |
| sucrose synthase | Ma07_p21330.1 | chr07 | 29451699 | 29455757 | Ma07_g21330~ sucrose synthase 1-like~ unknown_gene~ missing_functional_completeness |
| sucrose synthase | Ma08_p23180.1 | chr08 | 36576149 | 36584491 | Ma08_g23180~ sucrose synthase 4-like~ unknown_gene~ missing_functional_completeness |
| sucrose synthase | Ma09_p20960.1 | chr09 | 31409713 | 31413577 | Ma09_g20960~ Sucrose synthase 2~ unknown_gene~ complete |
| sucrose synthase | Ma10_p18610.1 | chr10 | 29481321 | 29485531 | Ma10_g18610~ sucrose synthase 2-like, transcript variant X2~ unknown_gene~ missing_functional_completeness |
| sucrose synthase | Ma10_p18610.2 | chr10 | 29481321 | 29485531 | Ma10_g18610~ sucrose synthase 2-like, transcript variant X2~ unknown_gene~ missing_functional_completeness |
| sucrose synthase | Ma10_p18610.3 | chr10 | 29481321 | 29485531 | Ma10_g18610~ sucrose synthase 2-like, transcript variant X2~  unknown_gene~ missing_functional_completeness |
| UDP-glucose 6-dehydrogenase | Ma03_p19560.1 | chr03 | 24848498 | 24849940 | Ma03_g19560~ UDP-glucose 6-dehydrogenase 4-like~ unknown_gene~ missing_  functional_completeness |
| UDP-glucose 6-dehydrogenase | Ma03_p31530.1 | chr03 | 33726073 | 33727515 | Ma03_g31530~ UDP-glucose 6-dehydrogenase 4~ unknown_gene~ missing_  functional_completeness |
| UDP-glucose 6-dehydrogenase | Ma04_p03400.1 | chr04 | 2626190 | 2627632 | Ma04_g03400~ UDP-glucose 6-dehydrogenase 4~ unknown_gene~ missing_functional_completeness |
| UDP-glucose 6-dehydrogenase | Ma04_p07270.1 | chr04 | 5275082 | 5276527 | Ma04_g07270~ UDP-glucose 6-dehydrogenase 4-like, transcript variant X1~  unknown_gene~ missing_functional_completeness |
| UDP-glucose 6-dehydrogenase | Ma04_p07270.2 | chr04 | 5275082 | 5276527 | Ma04_g07270~ UDP-glucose 6-dehydrogenase 4-like, transcript variant X1~unknown_gene~ missing_functional_completeness |
| UDP-glucose 6-dehydrogenase | Ma04_p07270.3 | chr04 | 5275082 | 5276527 | Ma04_g07270~ UDP-glucose 6-dehydrogenase 4-like, transcript variant X1~ unknown_gene~ missing_functional_Completeness |
| UDP-glucose 6-dehydrogenase | Ma04_p10810.1 | chr04 | 7659505 | 7660947 | Ma04_g10810~UDP-glucose 6-dehydrogenase 4-like~ unknown_gene~ missing_functional_completeness |
| UDP-glucose 6-dehydrogenase | Ma04_p14290.1 | chr04 | 10827627 | 10829069 | Ma04_g14290~ UDP-glucose 6-dehydrogenase 4~unknown_gene~missing_functional_completeness |
| UDP-glucose 6-dehydrogenase | Ma06_p12550.1 | chr06 | 8701224 | 8702666 | Ma06_g12550~ UDP-glucose 6-dehydrogenase 5-like~ unknown_gene~ missing_functional_completeness |
| UDP-glucose 6-dehydrogenase | Ma06_p25230.1 | chr06 | 25484560 | 25486002 | Ma06_g25230~ UDP-glucose 6-dehydrogenase 4-like~ unknown_gene~ missing_functional_completeness |

**Table S2**.*Cont*.

| Gene Name  Used in This  Research | Gene ID  In Banana  Genome Hub | Gene  Location | Start | End | Gene Annotation |  |
| --- | --- | --- | --- | --- | --- | --- |
| UDP-glucose 6-dehydrogenase | Ma07_p28080.1 | chr07 | 34250931 | 34252290 | Ma07_g28080~ UDP-glucose 6-dehydrogenase 4-like~ unknown_gene~ missing_functional_completeness |  |
| UDP-glucose 6-dehydrogenase | Ma07_p28090.1 | chr07 | 34254338 | 34254601 | Ma07_g28090~ UDP-glucose 6-dehydrogenase 1-like~ unknown_gene~ missing_functional_completeness |  |
| UDP-glucose 6-dehydrogenase | Ma10_p18290.1 | chr10 | 29273830 | 29275272 | Ma10_g18290~ UDP-glucose 6-dehydrogenase 4-like, transcript variant X2~ unknown_gene~ missing unctional_completeness |  |
| UDP-glucose 6-dehydrogenase | Ma10_p18290.2 | chr10 | 29273830 | 29275272 | Ma10_g18290~ UDP-glucose 6-dehydrogenase 4-like, transcript variant X2~ unknown_gene~ missing_ functional_completeness |  |
| UDP-glucose 6-dehydrogenase | Ma11_p09150.1 | chr11 | 7299283 | 7306372 | Ma11_g09150~ UDP-glucose 6-dehydrogenase 5-like~unknown_gene~ missing_functional_  Completeness |  |
| 2,3-bisphosphoglycerate-independent phosphoglycerate mutase | Ma04_p10290.1 | chr04 | 7342891 | 7348391 | Ma04_g10290~2,3-bisphosphoglycerate-independent phosphoglycerate mutase-like~  unknown_gene~ missing_functional_completeness |  |
| 2,3-bisphosphoglycerate-independent phosphoglycerate mutase | Ma07_p23360.1 | chr07 | 31035010 | 31043234 | Ma07_g23360~2,3-bisphosphoglycerate-independent phosphoglycerate mutase, transcript  variant X2~ unknown_gene~ missing_functional_completeness |  |
| 2,3-bisphosphoglycerate-independent phosphoglycerate mutase | Ma07_p23360.2 | chr07 | 31035010 | 31043234 | Ma07_g23360~2,3-bisphosphoglycerate-independent phosphoglycerate mutase, transcript  variant X2~ unknown_gene~ missing_functional_completeness |  |
| 2,3-bisphosphoglycerate-independent phosphoglycerate mutase | Ma07_p23360.3 | chr07 | 31035010 | 31043234 | Ma07_g23360~2,3-bisphosphoglycerate-independent phosphoglycerate mutase, transcript  variant X2~ unknown_gene~ missing_functional_completeness |  |
| 2,3-bisphosphoglycerate-  independent phosphoglycerate mutase | Ma09_p04830.1 | chr09 | 3108253 | 3109803 | Ma09_g04830~2,3-bisphosphoglycerate-independent phosphoglycerate mutase~ PGM1~ fragment |  |
| 2,3-bisphosphoglycerate-independent phosphoglycerate mutase | Ma09_p04840.1 | chr09 | 3111004 | 3113789 | Ma09_g04840~2,3-bisphosphoglycerate-independent phosphoglycerate mutase~ PGM1~ fragment |  |
| 2,3-bisphosphoglycerate-independent phosphoglycerate mutase | Ma10_p29170.1 | chr10 | 35830183 | 35835552 | Ma10_g29170~2,3-bisphosphoglycerate-independent phosphoglycerate mutase, transcript  variant X2~ unknown_gene~ missing_functional_completeness |  |
| 6-phosphofructokinase | Ma02_p18360.1 | chr02 | 24871930 | 24876386 | Ma02_g18360~ 6-phosphofructokinase 3~ pfp~ complete |  |
| 6-phosphofructokinase | Ma04_p01000.1 | chr04 | 885610 | 892035 | Ma04_g01000~ 6-phosphofructokinase 3~ pfp~ complete |  |
| 6-phosphofructokinase | Ma04_p08280.1 | chr04 | 5948415 | 5954645 | Ma04_g08280~ 6-phosphofructokinase 3~ pfp~ complete |  |
| 6-phosphofructokinase | Ma04_p15430.1 | chr04 | 11709406 | 11713186 | Ma04_g15430~ 6-phosphofructokinase 3~ pfp~ pseudogene |  |
| 6-phosphofructokinase | Ma04_p24700.1 | chr04 | 26656659 | 26662692 | Ma04_g24700~ 6-phosphofructokinase 3~ pfp~ complete |  |
| 6-phosphofructokinase | Ma05_p24880.1 | chr05 | 37017753 | 37020935 | Ma05_g24880~ 6-phosphofructokinase 2~ pfkA1~ complete | |
| 6-phosphofructokinase | Ma07_p10560.1 | chr07 | 7890072 | 7894434 | Ma07_g10560~ 6-phosphofructokinase 3~ pfkA~ pseudogene | |
| 6-phosphofructokinase | Ma07_p11340.1 | chr07 | 8423633 | 8430537 | Ma07_g11340~ 6-phosphofructokinase 5, chloroplastic~ pfkA2~ complete | |

**Table S2**.*Cont*.

| Gene Name  Used in This  Research | Gene ID  In Banana  Genome Hub | Gene  Location | Start | End | Gene Annotation |
| --- | --- | --- | --- | --- | --- |
| 6-phosphofructokinase | Ma07_p13520.1 | chr07 | 10169690 | 10173615 | Ma07_g13520~ 6-phosphofructokinase 3~ pfp~ complete |
| 6-phosphofructokinase | Ma09_p18430.1 | chr09 | 17917457 | 17918920 | Ma09_g18430~ 6-phosphofructokinase 2~ pfp~ complete |
| alcohol dehydrogenase | Ma02_p09950.1 | chr02 | 19697137 | 19699288 | Ma02_g09950~ Alcohol dehydrogenase 1~ ADH2~ complete |
| alcohol dehydrogenase | Ma04_p06170.1 | chr04 | 4555258 | 4556681 | Ma04_g06170~ cinnamyl alcohol dehydrogenase 2-like~ unknown_gene~ missing_functional_completeness |
| alcohol dehydrogenase | Ma04_p12960.1 | chr04 | 9804459 | 9806842 | Ma04_g12960~ cinnamyl alcohol dehydrogenase 2-like, transcript variant X2~ unknown_gene~  missing_functional_completeness |
| alcohol dehydrogenase | Ma04_p12960.2 | chr04 | 9804459 | 9807069 | Ma04_g12960~ cinnamyl alcohol dehydrogenase 2-like, transcript variant X2~ unknown_gene~ missing_functional_completeness |
| alcohol dehydrogenase | Ma04_p20820.1 | chr04 | 23534434 | 23538284 | Ma04_g20820~ probable cinnamyl alcohol dehydrogenase 1~ unknown_gene~ missing_functional_completeness |
| alcohol dehydrogenase | Ma04_p25530.1 | chr04 | 27271288 | 27275698 | Ma04_g25530~ Alcohol dehydrogenase class-3~ FDH~ complete |
| alcohol dehydrogenase | Ma04_p31940.1 | chr04 | 32111836 | 32116353 | Ma04_g31940~ Alcohol dehydrogenase-like 6~ ADH3~ complete |
| alcohol dehydrogenase | Ma04_p33020.1 | chr04 | 32856683 | 32858231 | Ma04_g33020~ probable cinnamyl alcohol dehydrogenase 1~ unknown_gene~ missing_functional_completeness |
| alcohol dehydrogenase | Ma04_p36000.1 | chr04 | 34517921 | 34521770 | Ma04_g36000~ Alcohol dehydrogenase-like 3~ ADH2~ complete |
| alcohol dehydrogenase | Ma05_p11250.1 | chr05 | 8140552 | 8142425 | Ma05_g11250~ probable cinnamyl alcohol dehydrogenase 6~ unknown_gene~ missing_functional_completeness |
| alcohol dehydrogenase | Ma06_p05880.1 | chr06 | 4322130 | 4351346 | Ma06_g05880~ zinc-binding alcohol dehydrogenase domain-containing protein 2,  transcript variant X1~ unknown_gene~ missing_functional_completeness |
| alcohol dehydrogenase | Ma06_p05880.2 | chr06 | 4322130 | 4351346 | Ma06_g05880~ zinc-binding alcohol dehydrogenase domain-containing protein 2,  transcript variant X1~ unknown_gene~ missing_functional_completeness |
| alcohol dehydrogenase | Ma06_p19900.1 | chr06 | 13988688 | 13990608 | Ma06_g19900~ Alcohol dehydrogenase 3~ ADH~ complete |
| alcohol dehydrogenase | Ma07_p11500.1 | chr07 | 8539962 | 8543179 | Ma07_g11500~ Alcohol dehydrogenase-like 7~ ADH~ fragment |
| alcohol dehydrogenase | Ma07_p11510.1 | chr07 | 8546813 | 8550412 | Ma07_g11510~ Alcohol dehydrogenase-like 7~ Os02g0815500~ complete |
| alcohol dehydrogenase | Ma08_p29910.1 | chr08 | 41352852 | 41355359 | Ma08_g29910~ Alcohol dehydrogenase 1~ ADH~ complete |
| alcohol dehydrogenase | Ma09_p08110.1 | chr09 | 5334827 | 5337766 | Ma09_g08110~ Alcohol dehydrogenase 2~ ADH~ complete |
| alcohol dehydrogenase | Ma10_p17990.1 | chr10 | 29138978 | 29140670 | Ma10_g17990~ probable cinnamyl alcohol dehydrogenase 9~ unknown_gene~ missing_functional_completeness |
| alcohol dehydrogenase | Ma11_p07340.1 | chr11 | 5828866 | 5830843 | Ma11_g07340~ Alcohol dehydrogenase 1~ ADH2~ complete |
| aldolase | Ma01_p00110.1 | chr01 | 104577 | 105077 | Ma01_g00110~putative 4-hydroxy-4-methyl-2-oxoglutarate aldolase 3, transcript variant X1~unknown_gene~ missing_functional_completeness |
| aldolase | Ma01_p00110.2 | chr01 | 104577 | 105077 | Ma01_g00110~ putative 4-hydroxy-4-methyl-2-oxoglutarate aldolase 3, transcript variant X1~ unknown_gene~ missing_functional_completeness |
| aldolase | Ma01_p14880.1 | chr01 | 10840654 | 10842684 | Ma01_g14880~ phospho-2-dehydro-3-deoxyheptonate aldolase 2, chloroplastic-like~ unknown_gene~ missing_functional_completeness |
| aldolase | Ma02_p14050.1 | chr02 | 22205971 | 22209128 | Ma02_g14050~ probable low-specificity L-threonine aldolase 1~ unknown_gene~ missing_functional_completeness |

**Table S2**.*Cont*.

| Gene Name  Used in This  Research | Gene ID  In Banana  Genome Hub | Gene  Location | Start | End | Gene Annotation |
| --- | --- | --- | --- | --- | --- |
| aldolase | Ma03_p11730.1 | chr03 | 9104927 | 9107389 | Ma03_g11730~ fructose-bisphosphate aldolase, cytoplasmic isozyme 1-like~  unknown_gene~missing_functional_completeness |
| aldolase | Ma03_p15050.1 | chr03 | 14821573 | 14822079 | Ma03_g15050~ putative 4-hydroxy-4-methyl-2-oxoglutarate aldolase 2~ unknown_gene~ missing_functional_Completeness |
| aldolase | Ma04_p05300.1 | chr04 | 3961099 | 3961605 | Ma04_g05300~ putative 4-hydroxy-4-methyl-2-oxoglutarate aldolase 1, transcript variant X1~unknown_gene~ missing_functional_completeness |
| aldolase | Ma04_p05300.2 | chr04 | 3961099 | 3961605 | Ma04_g05300~ putative 4-hydroxy-4-methyl-2-oxoglutarate aldolase 1, transcript variant X1~unknown_gene~ missing_functional_completeness |
| aldolase | Ma04_p11800.1 | chr04 | 8414330 | 8414836 | Ma04_g11800~ putative 4-hydroxy-4-methyl-2-oxoglutarate aldolase 2~ unknown_gene~ missing_functional_completeness |
| aldolase | Ma04_p35430.1 | chr04 | 34216197 | 34220389 | Ma04_35430~ phospho-2-dehydro-3-deoxyheptonate aldolase 1, chloroplastic-like~unknown_gene~ missing_functional_completeness |
| aldolase | Ma05_p19700.1 | chr05 | 28239181 | 28240038 | Ma05_g19700~Phospho-2-dehydro-3-deoxyheptonate aldolase 1, chloroplastic~ DAHPS1~ fragment |
| aldolase | Ma05_p22300.1 | chr05 | 34032047 | 34033642 | Ma05_g22300~ fructose-bisphosphate aldolase 1, chloroplastic~ unknown_gene~ missing_functional_completeness |
| aldolase | Ma05_p27790.1 | chr05 | 38933448 | 38936469 | Ma05_g27790~ fructose-bisphosphate aldolase cytoplasmic isozyme-like~ unknown_gene~ missing_functional_completeness |
| aldolase | Ma06_p11050.1 | chr06 | 7762827 | 7766709 | Ma06_g11050~ fructose-bisphosphate aldolase 1, chloroplastic-like~ unknown_gene~ missing_functional_completeness |
| aldolase | Ma06_p19280.1 | chr06 | 13242366 | 13244002 | Ma06_g19280~ fructose-bisphosphate aldolase, chloroplastic~ unknown_gene~ missing_functional_completeness |
| aldolase | Ma06_p21500.1 | chr06 | 15729162 | 15731466 | Ma06_g21500~ dihydroneopterin aldolase-like, transcript variant X2~ unknown_gene~ missing_functional_completeness |
| aldolase | Ma06_p21500.2 | chr06 | 15729162 | 15731466 | Ma06_g21500~ dihydroneopterin aldolase-like, transcript variant X2~ unknown_gene~ missing_functional_completeness |
| aldolase | Ma07_p12540.1 | chr07 | 9437560 | 9441145 | Ma07_g12540~ phospho-2-dehydro-3-deoxyheptonate aldolase 2, chloroplastic-like~ unknown_gene~missing_functional_completeness |
| aldolase | Ma07_p22590.1 | chr07 | 30469232 | 30473457 | Ma07_g22590~ [Fructose-bisphosphate aldolase]-lysine N-methyltransferase, chloroplastic-like,transcript variant X2~ unknown_gene~ missing_functional_completeness |
| aldolase | Ma07_p22620.1 | chr07 | 30495722 | 30500848 | Ma07_g22620~ [Fructose-bisphosphate aldolase]-lysine N-methyltransferase, chloroplastic-like~  unknown_gene~ missing_functional_completeness |
| aldolase | Ma08_p01140.1 | chr08 | 1061124 | 1063311 | Ma08_g01140~ fructose-bisphosphate aldolase cytoplasmic isozyme~ unknown_gene~ missing_functional_completeness |
| aldolase | Ma08_p07480.1 | chr08 | 5120544 | 5123633 | Ma08_g07480~ fructose-bisphosphate aldolase cytoplasmic isozyme-like, transcript variant X1~  unknown_gene~ missing_functional_completeness |
| aldolase | Ma08_p07480.2 | chr08 | 5120544 | 5123633 | Ma08_g07480~ fructose-bisphosphate aldolase cytoplasmic isozyme-like, transcript variant X1~  unknown_gene~ missing_functional_completeness |

**Table S2**.*Cont*.

| Gene Name  Used in This  Research | Gene ID  In Banana  Genome Hub | Gene  Location | Start | End | Gene Annotation |
| --- | --- | --- | --- | --- | --- |
| aldolase | Ma08_p08700.1 | chr08 | 6148508 | 6151704 | Ma08_g08700~ fructose-bisphosphate aldolase cytoplasmic isozyme-like~ unknown_gene~  missing_functional_completeness |
| aldolase | Ma08_p16120.1 | chr08 | 17083244 | 17085611 | Ma08_g16120~ fructose-bisphosphate aldolase, chloroplastic-like~ unknown_gene~missing_functional_completeness |
| aldolase | Ma08_p16810.1 | chr08 | 20778782 | 20781999 | Ma08_g16810~ fructose-bisphosphate aldolase 1, chloroplastic-like~ unknown_gene~ missing_functional_completeness |
| aldolase | Ma08_p29530.1 | chr08 | 41090337 | 41103884 | Ma08_g29530~ 2-dehydro-3-deoxyphosphooctonate aldolase~ unknown_gene~ missing_functional_completeness |
| aldolase | Ma09_p29310.1 | chr09 | 39788075 | 39790321 | Ma09_g29310~ dihydroneopterin aldolase-like~ unknown_gene~ missing_functional_completeness |
| aldolase | Ma09_p31410.1 | chr09 | 41149275 | 41151595 | Ma09_g31410~ dihydroneopterin aldolase-like, transcript variant X1~ unknown_gene~ missing_functional_completeness |
| aldolase | Ma09_p31410.2 | chr09 | 41149275 | 41169523 | Ma09_g31410~ dihydroneopterin aldolase-like, transcript variant X1~ unknown_gene~ missing_functional_completeness |
| aldolase | Ma09_p31420.1 | chr09 | 41155528 | 41157762 | Ma09_g31420~ dihydroneopterin aldolase-like~ unknown_gene~missing_functional_completeness |
| aldolase | Ma10_p10680.1 | chr10 | 24499808 | 24500398 | Ma10_g10680~ fructose-bisphosphate aldolase, chloroplastic-like~ unknown_gene~ missing_functional_completeness |
| aldolase | Ma10_p24640.1 | chr10 | 33139892 | 33143015 | Ma10_g24640~ phospho-2-dehydro-3-deoxyheptonate aldolase 2, chloroplastic-like~  unknown_gene~ missing_functional_completeness |
| aldolase | Ma10_p30330.1 | chr10 | 36652311 | 36655242 | Ma10_g30330~ phospho-2-dehydro-3-deoxyheptonate aldolase 2, chloroplastic-like~  unknown_gene~ missing_functional_completeness |
| aldolase | Ma11_p03650.1 | chr11 | 2719211 | 2725378 | Ma11_g03650~ probable transaldolase~ unknown_gene~ missing_functional_completeness |
| aldolase | Ma11_p22910.1 | chr11 | 26483024 | 26495256 | Ma11_g22910~ [Fructose-bisphosphate aldolase]-lysine N-methyltransferase, chloroplastic,  transcript variant X2~ unknown_gene~ missing_functional_completeness |
| aldolase | Ma11_p22910.2 | chr11 | 26483024 | 26495256 | Ma11_g22910~ [Fructose-bisphosphate aldolase]-lysine N-methyltransferase, chloroplastic,  transcript variant X2~ unknown_gene~ missing_functional_completeness |
| aldolase | Ma11_p25030.1 | chr11 | 27796560 | 27796955 | Ma11_g25030~ Fructose-bisphosphate aldolase cytoplasmic isozyme~ FBA~ fragment |
| aldolase | Ma11_p25040.1 | chr11 | 27796964 | 27800885 | Ma11_g25040~ Fructose-bisphosphate aldolase cytoplasmic isozyme~ FBA~ fragment |
| Aldose 1-epimerase | Ma05_p25750.1 | chr05 | 37592782 | 37595266 | Ma05_g25750~ aldose 1-epimerase-like~ unknown_gene~ missing_functional_completeness |
| aldose 1-epimerase | Ma06_p23880.1 | chr06 | 22056975 | 22059413 | Ma06_g23880~ aldose 1-epimerase-like~ unknown_gene~ missing_functional_completeness |
| aldose 1-epimerase | Ma08_p34760.1 | chr08 | 44752873 | 44755226 | Ma08_g34760~ aldose 1-epimerase-like~ unknown_gene~ missing_functional_completeness |
| aldose 1-epimerase | Ma09_p11280.1 | chr09 | 7625588 | 7628750 | Ma09_g11280~ aldose 1-epimerase-like~ unknown_gene~ missing_functional_completeness |
| dihydrolipoyl dehydrogenase | Ma01_p11770.1 | chr01 | 8534387 | 8546449 | Ma01_g11770~ dihydrolipoyl dehydrogenase 1, chloroplastic-like~ unknown_gene~ missing_functional_completeness |
| dihydrolipoyl dehydrogenase | Ma02_p03980.1 | chr02 | 15413307 | 15417984 | Ma02_g03980~ dihydrolipoyl dehydrogenase, mitochondrial-like~ unknown_gene~ missing_functional_completeness |
| dihydrolipoyl dehydrogenase | Ma03_p12420.1 | chr03 | 9579211 | 9592446 | Ma03_g12420~ dihydrolipoyl dehydrogenase 2, chloroplastic-like~ unknown_gene~ missing_functional_completeness |

**Table S2**.*Cont*.

| Gene Name  Used in This  Research | Gene ID  In Banana  Genome Hub | Gene  Location | Start | End | Gene Annotation |
| --- | --- | --- | --- | --- | --- |
| dihydrolipoyl dehydrogenase | Ma08_p22830.1 | chr08 | 36315704 | 36316032 | Ma08_g22830~ Putative Dihydrolipoyl dehydrogenase~ lpdA~ fragment |
| dihydrolipoyl dehydrogenase | Ma11_p12560.1 | chr11 | 16469206 | 16473715 | Ma11_g12560~ dihydrolipoyl dehydrogenase, mitochondrial-like~ unknown_gene~ missing_functional_completeness |
| dihydrolipoyllysine-residue acetyltransferase | Ma03_p07300.1 | chr03 | 5092566 | 5096206 | Ma03_g07300~ dihydrolipoyllysine-residue acetyltransferase component of pyruvate dehydrogenase complex, mitochondrial~ unknown_gene~ missing_functional_completeness |
| dihydrolipoyllysine-residue  acetyltransferase | Ma03_p18460.1 | chr03 | 24089928 | 24095495 | Ma03_g18460~ dihydrolipoyllysine-residue acetyltransferase component of pyruvate dehydrogenase complex, mitochondrial~ unknown_gene~ missing_functional_completeness |
| dihydrolipoyllysine-residue  acetyltransferase | Ma08_p17990.1 | chr08 | 27759660 | 27793869 | Ma08_g17990~ dihydrolipoyllysine-residue acetyltransferase component 1 of pyruvate dehydrogenase complex, mitochondrial, transcript variant X1~ unknown_gene~ missing_functional_completeness |
| dihydrolipoyllysine-  residue acetyltransferase | Ma03_p07300.1 | chr03 | 5092566 | 5096206 | Ma03_g07300~ dihydrolipoyllysine-residue acetyltransferase component of pyruvate dehydrogenase complex, mitochondrial~ unknown_gene~ missing_functional_completeness |
| dihydrolipoyllysine-  residue acetyltransferase | Ma03_p18460.1 | chr03 | 24089928 | 24095495 | Ma03_g18460~ dihydrolipoyllysine-residue acetyltransferase component of pyruvate dehydrogenase complex, mitochondrial~ unknown_gene~ missing_functional_completeness |
| dihydrolipoyllysine-  residue acetyltransferase | Ma08_p17990.2 | chr08 | 27759660 | 27796760 | Ma08_g17990~ dihydrolipoyllysine-residue acetyltransferase component 1 of pyruvate dehydrogenase complex, mitochondrial, transcript variant X1~ unknown_gene~ missing_functional_completeness |
| dihydrolipoyllysine-  residue acetyltransferase | Ma08_p17990.3 | chr08 | 27759660 | 27796760 | Ma08_g17990~ dihydrolipoyllysine-residue acetyltransferase component 1 of pyruvate dehydrogenase complex, mitochondrial, transcript variant X1~ unknown_gene~ missing_functional_completeness |
| dihydrolipoyllysine-  residue acetyltransferase | Ma09_p08190.1 | chr09 | 5393185 | 5396657 | Ma09_g08190~ dihydrolipoyllysine-residue acetyltransferase component of pyruvate dehydrogenase complex, mitochondrial-like~ unknown_gene~ missing_functional_completeness |
| dihydrolipoyllysine-  residue acetyltransferase | Ma10_p09080.1 | chr10 | 23277888 | 23294714 | Ma10_g09080~ dihydrolipoyllysine-residue acetyltransferase component 3 of pyruvate dehydrogenase complex, mitochondrial-like~ unknown_gene~ missing_functional_completeness |
| fructose-bisphosphate aldolase | Ma03_p11730.1 | chr03 | 9104927 | 9107389 | Ma03_g11730~ fructose-bisphosphate aldolase, cytoplasmic isozyme 1-like~ unknown_  gene~ missing_functional_completeness |
| fructose-bisphosphate aldolase | Ma05_p22300.1 | chr5 | 34032047 | 34033642 | Ma05_g22300~ fructose-bisphosphate aldolase 1, chloroplastic~ unknown_gene~ missing_functional_completeness |
| fructose-bisphosphate aldolase | Ma05_p27790.1 | chr5 | 38933448 | 38936469 | Ma05_g27790~ fructose-bisphosphate aldolase cytoplasmic isozyme-like~ unknown_gene~  missing_functional_completeness |
| fructose-bisphosphate aldolase | Ma06_p11050.1 | chr6 | 7762827 | 7766709 | Ma06_g11050~ fructose-bisphosphate aldolase 1, chloroplastic-like~ unknown_  gene~ missing_functional_completeness |
| fructose-bisphosphate aldolase | Ma06_p19280.1 | chr6 | 13242366 | 13244002 | Ma06_g19280~ fructose-bisphosphate aldolase, chloroplastic~ unknown_gene~ missing_functional_completeness |
| fructose-bisphosphate aldolase | Ma07_p22590.1 | chr7 | 30469232 | 30473457 | Ma07_g22590~ [Fructose-bisphosphate aldolase]-lysine N-methyltransferase,  chloroplastic-like, transcript variant X2~ unknown_gene~ missing_functional_completeness |
| fructose-bisphosphate aldolase | Ma07_p22590.2 | chr7 | 30469232 | 30473457 | Ma07_g22590~ [Fructose-bisphosphate aldolase]-lysine N-methyltransferase,  chloroplastic-like, transcript variant X2~ unknown_gene~ missing_functional_completeness |

**Table S2**.*Cont*.

| Gene Name  Used in This  Research | Gene ID  In Banana  Genome Hub | Gene  Location | Start | End | Gene Annotation |
| --- | --- | --- | --- | --- | --- |
| fructose-bisphosphate aldolase | Ma07_p22620.1 | chr7 | 30495722 | 30500848 | Ma07_g22620~ [Fructose-bisphosphate aldolase]-lysine N-methyltransferase,  chloroplastic-like~ unknown_gene~ missing_functional_completeness |
| fructose-bisphosphate aldolase | Ma08_p01140.1 | chr08 | 1061124 | 1063311 | Ma08_g01140~ fructose-bisphosphate aldolase cytoplasmic isozyme~ unknown_  gene~ missing_functional_completeness |
| fructose-bisphosphate aldolase | Ma08_p07480.1 | chr08 | 5120544 | 5123633 | Ma08_g07480~ fructose-bisphosphate aldolase cytoplasmic isozyme-like,  transcript variant X1~ unknown_gene~ missing_functional_completeness |
| fructose-bisphosphate aldolase | Ma08_p07480.2 | chr08 | 5120544 | 5123633 | Ma08_g07480~ fructose-bisphosphate aldolase cytoplasmic isozyme-like,  transcript variant X1~ unknown_gene~ missing_functional_completeness |
| fructose-bisphosphate  aldolase | Ma08_p08700.1 | chr08 | 6148508 | 6151704 | Ma08_g08700~ fructose-bisphosphate aldolase cytoplasmic isozyme-like~ unknown_  gene~ missing_functional_completeness |
| fructose-bisphosphate  aldolase | Ma08_p16120.1 | chr08 | 17083244 | 17085611 | Ma08_g16120~ fructose-bisphosphate aldolase, chloroplastic-like~ unknown_gene~ missing_functional_completeness |
| fructose-bisphosphate  aldolase | Ma08_p16810.1 | chr08 | 20778782 | 20781999 | Ma08_g16810~ fructose-bisphosphate aldolase 1, chloroplastic-like~ unknown_gene~missing_functional_completeness |
| fructose-bisphosphate  aldolase | Ma10_p10680.1 | chr10 | 24499808 | 24500398 | Ma10_g10680~ fructose-bisphosphate aldolase, chloroplastic-like~ unknown_gene~missing_functional_completeness |
| fructose-bisphosphate  aldolase | Ma11_p22910.1 | chr11 | 26483024 | 26495256 | Ma11_g22910~ [Fructose-bisphosphate aldolase]-lysine N-methyltransferase,  chloroplastic, transcript variant X2~ unknown_gene~ missing_functional_completeness |
| fructose-bisphosphate  aldolase | Ma11_p22910.2 | chr11 | 26483024 | 26495256 | Ma11_g22910~ [Fructose-bisphosphate aldolase]-lysine N-methyltransferase,  chloroplastic, transcript variant X2~ unknown_gene~ missing_functional_completeness |
| fructose-bisphosphate  aldolase | Ma11_p25030.1 | chr11 | 27796560 | 27796955 | Ma11_g25030~ Fructose-bisphosphate aldolase cytoplasmic isozyme~ FBA~ fragment |
| fructose-bisphosphate  aldolase | Ma11_p25040.1 | chr11 | 27796964 | 27800885 | Ma11_g25040~ Fructose-bisphosphate aldolase cytoplasmic isozyme~ FBA~ fragment |
| glucose-6-phosphate isomerase | Ma07_p08070.1 | chr07 | 6016867 | 6025844 | Ma07_g08070~ glucose-6-phosphate isomerase, cytosolic, transcript variant X1~ unknown_gene~missing_  functional_completeness |
| glucose-6-phosphate isomerase | Ma07_p08070.2 | chr07 | 6016867 | 6025844 | Ma07_g08070~ glucose-6-phosphate isomerase, cytosolic, transcript variant X1~ unknown_gene~  missing_functional_completeness |
| glucose-6-phosphate isomerase | Ma09_p14520.1 | chr09 | 9911884 | 9922066 | Ma09_g14520~ glucose-6-phosphate isomerase 1, chloroplastic-like~ unknown_gene~missing_functional_completeness |
| glucose-6-phosphate isomerase | Ma10_p24940.1 | chr10 | 33300445 | 33308162 | Ma10_g24940~ glucose-6-phosphate isomerase 1, chloroplastic-like~ unknown_gene~ missing_functional_completeness |
| glucose-6-phosphate isomerase | Ma10_p28640.1 | chr10 | 35455241 | 35470300 | Ma10_g28640~ glucose-6-phosphate isomerase, cytosolic 2B-like~ unknown_gene~ missing_unctional_completeness |
| glyceraldehyde-3-phosphate dehydrogenase | Ma01_p11940.1 | chr01 | 8654885 | 8655250 | Ma01_g11940~ glyceraldehyde-3-phosphate dehydrogenase, testis-specific~ unknown_gene~ missing_functional_completeness |
| glyceraldehyde-3-phosphate dehydrogenase | Ma02_p04500.1 | chr02 | 15907679 | 15908105 | Ma02_g04500~ Glyceraldehyde-3-phosphate dehydrogenase~ gapdh~ fragment |

**Table S2**.*Cont*.

| Gene Name  Used in This  Research | Gene ID  In Banana  Genome Hub | Gene  Location | Start | End | Gene Annotation |
| --- | --- | --- | --- | --- | --- |
| glyceraldehyde-3-  phosphate dehydrogenase | Ma02_p04510.1 | chr02 | 15908123 | 15908890 | Ma02_g04510~ Glyceraldehyde-3-phosphate dehydrogenase, cytosolic~ GAPC~ fragment |
| glyceraldehyde-3-  phosphate dehydrogenase | Ma05_p00210.1 | chr05 | 153041 | 158138 | Ma05_g00210~ glyceraldehyde-3-phosphate dehydrogenase GAPCP2, chloroplastic-like~ unknown_gene~ missing_functional_completeness |
| glyceraldehyde-3-  phosphate dehydrogenase | Ma05_p17770.1 | chr05 | 21614196 | 21615064 | Ma05_g17770~ glyceraldehyde-3-phosphate dehydrogenase, putative, expressed~ GAPC1~ fragment |
| glyceraldehyde-3-  phosphate dehydrogenase | Ma05_p27700.1 | chr05 | 38887130 | 38891852 | Ma05_g27700~ glyceraldehyde-3-phosphate dehydrogenase 2, cytosolic~ unknown_gene~ missing_functional_completeness |
| glyceraldehyde-3-  phosphate dehydrogenase | Ma06_p01470.1 | chr06 | 1190774 | 1194336 | Ma06_g01470~ glyceraldehyde-3-phosphate dehydrogenase 2, cytosolic-like~ unknown_gene~ missing_functional_completeness |
| glyceraldehyde-3-  phosphate dehydrogenase | Ma06_p03680.1 | chr06 | 2676010 | 2681182 | Ma06_g03680~ NADP-dependent glyceraldehyde-3-phosphate dehydrogenase~ unknown_gene~ missing_functional_completeness |
| glyceraldehyde-3-  phosphate dehydrogenase | Ma06_p04220.1 | chr06 | 3061274 | 3061714 | Ma06_g04220~ glyceraldehyde-3-phosphate dehydrogenase, testis-specific-like~ unknown_gene~ missing_functional_completeness |
| glyceraldehyde-3-  phosphate dehydrogenase | Ma06_p17760.1 | chr06 | 12055149 | 12057250 | Ma06_g17760~ glyceraldehyde-3-phosphate dehydrogenase A, chloroplastic-like~ unknown_gene~missing_functional_completeness |
| glyceraldehyde-3-  phosphate dehydrogenase | Ma07_p03670.1 | chr07 | 2807539 | 2814308 | Ma07_g03670~ glyceraldehyde-3-phosphate dehydrogenase GAPCP2, chloroplastic-like~ unknown_gene~missing_functional_completeness |
| glyceraldehyde-3-  phosphate dehydrogenase | Ma07_p20820.1 | chr07 | 28829713 | 28832911 | Ma07_g20820~ NADP-dependent glyceraldehyde-3-phosphate dehydrogenase-like~ unknown_gene~missing_functional_completeness |
| glyceraldehyde-3-  phosphate dehydrogenase | Ma08_p33830.1 | chr08 | 44031420 | 44037840 | Ma08_g33830~ glyceraldehyde-3-phosphate dehydrogenase GAPCP1, chloroplastic-like~ unknown_gene~missing_functional_completeness |
| glyceraldehyde-3-  phosphate dehydrogenase | Ma09_p02110.1 | chr09 | 1536946 | 1540648 | Ma09_g02110~ glyceraldehyde-3-phosphate dehydrogenase 2, cytosolic-like~ unknown_gene~  missing_functional_completeness |
| glyceraldehyde-3-  phosphate dehydrogenase | Ma10_p12550.1 | chr10 | 25599210 | 25602699 | Ma10_g12550~ glyceraldehyde-3-phosphate dehydrogenase A, chloroplastic~ unknown_gene~  missing_functional_completeness |
| glyceraldehyde-3-  phosphate dehydrogenase | Ma11_p01390.1 | chr11 | 977738 | 981496 | Ma11_g01390~ glyceraldehyde-3-phosphate dehydrogenase B, chloroplastic-like~ unknown_gene~missing_functional_completeness |
| glyceraldehyde-3-  phosphate dehydrogenase | Ma11_p08300.1 | chr11 | 6609958 | 6613558 | Ma11_g08300~ glyceraldehyde-3-phosphate dehydrogenase 2, cytosolic-like~ unknown_gene~  missing_functional_completeness |
| glyceraldehyde-3-  phosphate dehydrogenase | Ma11_p17540.1 | chr11 | 22844667 | 22848537 | Ma11_g17540~ glyceraldehyde-3-phosphate dehydrogenase 2, cytosolic-like~ unknown_gene~  missing_functional_completeness |
| glyceraldehyde-3-  phosphate dehydrogenase | Ma11_p20650.1 | chr11 | 25051336 | 25053582 | Ma11_g20650~ glyceraldehyde-3-phosphate dehydrogenase B, chloroplastic, transcript variant X2~unknown_gene~ missing_functional_completeness |
| glyceraldehyde-3-  phosphate dehydrogenase | Ma11_p20650.2 | chr11 | 25051336 | 25053582 | Ma11_g20650~ glyceraldehyde-3-phosphate dehydrogenase B, chloroplastic, transcript variant X2~unknown_gene~ missing_functional_completeness |
| hexokinase | Ma01_p05010.1 | chr01 | 3499938 | 3509769 | Ma01_g05010~ hexokinase-3-like~ unknown_gene~ missing_functional_completeness |

**Table S2**.*Cont*.

| Gene Name  Used in This  Research | Gene ID  In Banana  Genome Hub | Gene  Location | Start | End | Gene Annotation |
| --- | --- | --- | --- | --- | --- |
| hexokinase | Ma03_p16610.1 | chr03 | 19175906 | 19181474 | Ma03_g16610~ hexokinase-2-like~ unknown_gene~ missing_functional_completeness |
| hexokinase | Ma03_p32880.1 | chr03 | 34485478 | 34490214 | Ma03_g32880~ hexokinase-2-like~ unknown_gene~ missing_functional_completeness |
| hexokinase | Ma06_p07130.1 | chr06 | 5092760 | 5095193 | Ma06_g07130~ Hexokinase-2~ HXK1~ missing_completeness |
| hexokinase | Ma06_p28110.1 | chr06 | 29880977 | 29888258 | Ma06_g28110~ hexokinase-2-like~ unknown_gene~ missing_functional_completeness |
| hexokinase | Ma08_p09550.1 | chr08 | 6912537 | 6917118 | Ma08_g09550~ Hexokinase-3~ HXK3~ complete |
| hexokinase | Ma08_p09560.1 | chr08 | 6923759 | 6924034 | Ma08_g09560~ Hexokinase-1~ HXK1~ fragment |
| hexokinase | Ma08_p28220.1 | chr08 | 40234415 | 40240028 | Ma08_g28220~ hexokinase-2-like~ unknown_gene~ missing_functional_completeness |
| hexokinase | Ma08_p29950.1 | chr08 | 41374068 | 41381741 | Ma08_g29950~ Hexokinase-3~ HXK1~ missing_completeness |
| hexokinase | Ma09_p05860.1 | chr09 | 3787033 | 3789015 | Ma09_g05860~ Putative Hexokinase-2~ HXK2~ fragment |
| hexokinase | Ma11_p05730.1 | chr11 | 4414787 | 4423529 | Ma11_g05730~ hexokinase-3-like, transcript variant X2~ unknown_gene~ missing_functional_completeness |
| hexokinase | Ma11_p05730.2 | chr11 | 4414787 | 4423529 | Ma11_g05730~ hexokinase-3-like, transcript variant X2~ unknown_gene~ missing_functional_completeness |
| hexokinase | Ma11_p05730.3 | chr11 | 4414787 | 4423529 | Ma11_g05730~ hexokinase-3-like, transcript variant X2~ unknown_gene~ missing_functional_completeness |
| hexokinase | Ma11_p05730.4 | chr11 | 4414787 | 4423529 | Ma11_g05730~ hexokinase-3-like, transcript variant X2~ unknown_gene~ missing_functional_completeness |
| phosphoenolpyruvate carboxykinase | Ma04_p27480.1 | chr04 | 28655157 | 28662444 | Ma04_g27480~ phosphoenolpyruvate carboxykinase [ATP]-like~ unknown_gene~ missing_functional_completeness |
| phosphoenolpyruvate carboxykinase | Ma04_p28650.1 | chr04 | 29668523 | 29671758 | Ma04_g28650~ phosphoenolpyruvate carboxykinase [ATP]-like~ unknown_gene~ missing_functional_completeness |
| phosphoenolpyruvate carboxykinase | Ma08_p14790.1 | chr08 | 14730212 | 14733553 | Ma08_g14790~ phosphoenolpyruvate carboxykinase [ATP]-like, transcript variant X2~ unknown_gene~missing_functional_completeness |
| phosphoenolpyruvate carboxykinase | Ma08_p14790.2 | chr08 | 14730212 | 14734274 | Ma08_g14790~ phosphoenolpyruvate carboxykinase [ATP]-like, transcript variant X2~ unknown_gene~missing_functional_completeness |
