# Supplementary material for: Expression Fluctuations of Genes Involved in Carbohydrate Metabolism Affected by Alterations of Ethylene Biosynthesis Associated with Ripening in Banana Fruit
Source: Plants (Basel). 2020 Aug 30;9(9):1120. doi: 10.3390/plants9091120 (PMC7570234; doi:10.3390/plants9091120)
Supplement: Supplementary file 1 [file plants-09-01120-s001.zip › Table_S3.docx]

# Table S3. Genes in glycolysis and gluconeogenesis used for Blast analysis in this study. The gene annotations are obtained from Banana Genome Hub.

| Gene Name  Used in this  Research | Gene ID in  Banana Genome  Hub | Gene  Location | Start | End | Gene Annotation |
| --- | --- | --- | --- | --- | --- |
| Phosphoglucomutase | Ma02_p08630.1 | chr02 | 18912151 | 18920306 | Ma02_g08630~ phosphoglucomutase, cytoplasmic 2-like~ unknown_gene~ missing_functional_completeness |
| Phosphoglucomutase | Ma08_p15070.1 | chr08 | 15015087 | 15022457 | Ma08_g15070~ phosphoglucomutase, chloroplastic~ unknown_gene~ missing_functional_completeness |
| Phosphoglucomutase | Ma08_p31340.1 | chr08 | 42285583 | 42294687 | Ma08_g31340~ phosphoglucomutase, cytoplasmic 2~ unknown_gene~ missing_functional_completeness |
| pyruvate decarboxylase | Ma01_p22480.1 | chr01 | 25104475 | 25106718 | Ma01_g22480~ Pyruvate decarboxylase isozyme 1~ PDC3~ complete |
| pyruvate decarboxylase | Ma03_p09880.1 | chr03 | 7352328 | 7354702 | Ma03_g09880~ Pyruvate decarboxylase isozyme 2~ PDC3~ complete |
| pyruvate decarboxylase | Ma04_p16540.1 | chr04 | 15944615 | 15948829 | Ma04_g16540~ Pyruvate decarboxylase isozyme 1~ PDC3~ complete |
| pyruvate decarboxylase | Ma05_p07150.1 | chr05 | 5209629 | 5211926 | Ma05_g07150~ Pyruvate decarboxylase isozyme 2~ PDC3~ complete |
| pyruvate decarboxylase | Ma05_p30490.1 | chr05 | 40880480 | 40882769 | Ma05_g30490~ Pyruvate decarboxylase isozyme 2~ PDC3~ complete |
| pyruvate decarboxylase | Ma07_p25850.1 | chr07 | 32821239 | 32825545 | Ma07_g25850~ Putative Pyruvate decarboxylase isozyme 1~ PDC3~ pseudogene |
| pyruvate decarboxylase | Ma11_p23090.1 | chr11 | 26585664 | 26587913 | Ma11_g23090~ Pyruvate decarboxylase isozyme 2~ unknown_gene~ complete |
| pyruvate dehydrogenase E1 component | Ma01_p11600.1 | chr01 | 8385865 | 8391696 | Ma01_g11600~ pyruvate dehydrogenase E1 component subunit alpha-1, mitochondrial-like~ unknown_gene~ missing_functional_completeness |
| pyruvate dehydrogenase E1 component | Ma02_p14810.1 | chr02 | 22746876 | 22747064 | Ma02_g14810~ Pyruvate dehydrogenase E1 component subunit beta~ pdhB~ fragment |
| pyruvate dehydrogenase E1 component | Ma04_p26730.1 | chr04 | 28099280 | 28099432 | Ma04_g26730~ Pyruvate dehydrogenase E1 component subunit alpha-1, mitochondrial~ At1g59900~ fragment |
| pyruvate dehydrogenase E1 component | Ma04_p26740.1 | chr04 | 28099493 | 28102926 | Ma04_g26740~ Pyruvate dehydrogenase E1 component subunit alpha-2, mitochondrial~ IAR4~ fragment |
| pyruvate dehydrogenase E1 component | Ma04_p26750.1 | chr04 | 28103066 | 28103621 | Ma04_g26750~ Pyruvate dehydrogenase E1 component subunit alpha-1, mitochondrial~ At1g59900~ fragment |
| pyruvate dehydrogenase E1 component | Ma04_p38050.1 | chr04 | 35740777 | 35742096 | Ma04_g38050~ Pyruvate dehydrogenase E1 component subunit beta~ pdhB~ complete |
| pyruvate dehydrogenase E1 component | Ma05_p02480.1 | chr05 | 1594148 | 1597888 | Ma05_g02480~ pyruvate dehydrogenase E1 component subunit beta~ unknown_gene~ missing_functional_completeness |
| pyruvate dehydrogenase E1 component | Ma05_p21130.1 | chr05 | 32832959 | 32835903 | Ma05_g21130~ pyruvate dehydrogenase E1 component subunit alpha-3, chloroplastic-like~ unknown_gene~missing_functional_completeness |
| pyruvate dehydrogenase E1 component | Ma05_p27260.1 | chr05 | 38628305 | 38634533 | Ma05_g27260~ pyruvate dehydrogenase E1 component subunit beta-1, mitochondrial~ unknown_gene~ missing_functional_completeness |
| pyruvate dehydrogenase E1 component | Ma07_p05600.1 | chr07 | 4053515 | 4058677 | Ma07_g05600~ pyruvate dehydrogenase E1 component subunit alpha-1, mitochondrial-like~ unknown_gene~ missing_functional_completeness |
| pyruvate dehydrogenase E1 component | Ma08_p08880.1 | chr08 | 6313328 | 6316668 | Ma08_g08880~ Pyruvate dehydrogenase E1 component subunit beta, mitochondrial~ PDH2~ fragment |
| pyruvate dehydrogenase E1 component | Ma10_p29610.1 | chr10 | 36141963 | 36150350 | Ma10_g29610~ pyruvate dehydrogenase E1 component subunit alpha-1, mitochondrial-like~ unknown_gene~ missing_functional_completeness |

Table S3. *Cont*.

| Gene Name  Used in this  Research | Gene ID in  Banana Genome  Hub | Gene  Location | Start | End | Gene Annotation |
| --- | --- | --- | --- | --- | --- |
| pyruvate dehydrogenase E1 component | Ma11_p20960.1 | chr11 | 25296643 | 25299288 | Ma11_g20960~ pyruvate dehydrogenase E1 component subunit alpha-3, chloroplastic-like~ unknown_gene~ missing_functional_completeness |
| pyruvate kinase | Ma01_p17610.1 | chr01 | 12895513 | 12897766 | Ma01_g17610~ Pyruvate kinase~ PKM2~ complete |
| pyruvate kinase | Ma02_p05810.1 | chr02 | 16999492 | 17002657 | Ma02_g05810~ Pyruvate kinase, cytosolic isozyme~ pkiA~ complete |
| pyruvate kinase | Ma02_p19270.1 | chr02 | 25492410 | 25500153 | Ma02_g19270~ Pyruvate kinase isozyme A, chloroplastic~ pyk~ complete |
| pyruvate kinase | Ma02_p22100.1 | chr02 | 27533876 | 27542886 | Ma02_g22100~ Putative Pyruvate kinase, cytosolic isozyme~ pkiA~ complete |
| pyruvate kinase | Ma03_p28290.1 | chr03 | 31411983 | 31416934 | Ma03_g28290~ Pyruvate kinase, cytosolic isozyme~ PKM2~ complete |
| pyruvate kinase | Ma03_p33150.1 | chr03 | 34616911 | 34621334 | Ma03_g33150~ Putative Pyruvate kinase isozyme G, chloroplastic~ pyk~ remnant |
| pyruvate kinase | Ma04_p27720.1 | chr04 | 28890595 | 28898105 | Ma04_g27720~ Pyruvate kinase isozyme A, chloroplastic~ pyk1~ complete |
| pyruvate kinase | Ma04_p28340.1 | chr04 | 29401125 | 29408200 | Ma04_g28340~ Pyruvate kinase isozyme G, chloroplastic~ pyk~ complete |
| pyruvate kinase | Ma04_p35050.1 | chr04 | 34001188 | 34005980 | Ma04_g35050~ Pyruvate kinase isozyme A, chloroplastic~ pyk~ complete |
| pyruvate kinase | Ma04_p35390.1 | chr04 | 34191276 | 34197170 | Ma04_g35390~ Pyruvate kinase isozyme G, chloroplastic~ pyk~ complete |
| pyruvate kinase | Ma05_p20410.1 | chr05 | 32100667 | 32101999 | Ma05_g20410~ Pyruvate kinase isozyme G, chloroplastic~ pkiA~ remnant |
| pyruvate kinase | Ma05_p20420.1 | chr05 | 32102214 | 32102986 | Ma05_g20420~ Pyruvate kinase isozyme G, chloroplastic~ pykF~ remnant |
| pyruvate kinase | Ma05_p20430.1 | chr05 | 32105103 | 32105378 | Ma05_g20430~ Pyruvate kinase~ pyk~ remnant |
| pyruvate kinase | Ma05_p30050.1 | chr05 | 40583885 | 40586993 | Ma05_g30050~ Pyruvate kinase, cytosolic isozyme~ PKM2~ complete |
| pyruvate kinase | Ma06_p14950.1 | chr06 | 10182281 | 10190254 | Ma06_g14950~ Putative Pyruvate kinase, cytosolic isozyme~ pkiA~ complete |
| pyruvate kinase | Ma06_p17840.1 | chr06 | 12118394 | 12119005 | Ma06_g17840~ Pyruvate kinase, cytosolic isozyme~ Pkm2~ remnant |
| pyruvate kinase | Ma06_p38800.1 | chr06 | 37438357 | 37438964 | Ma06_g38800~ Pyruvate kinase, cytosolic isozyme~ pyk~ remnant |
| pyruvate kinase | Ma07_p17230.1 | chr07 | 16667646 | 16710150 | Ma07_g17230~ Pyruvate kinase isozyme A, chloroplastic~ pykF~ pseudogene |
| pyruvate kinase | Ma07_p23210.1 | chr07 | 30943911 | 30945416 | Ma07_g23210~ Pyruvate kinase, cytosolic isozyme~ pkiA~ complete |
| pyruvate kinase | Ma07_p26120.1 | chr07 | 33046978 | 33053835 | Ma07_g26120~ Pyruvate kinase isozyme G, chloroplastic~ pyk~ complete |
| pyruvate kinase | Ma08_p13380.1 | chr08 | 10554685 | 10559541 | Ma08_g13380~ Pyruvate kinase, cytosolic isozyme~ PKM2~ complete |
| pyruvate kinase | Ma08_p15530.1 | chr08 | 15729139 | 15735100 | Ma08_g15530~ Pyruvate kinase isozyme G, chloroplastic~ pyk~ complete |
| pyruvate kinase | Ma09_p08070.1 | chr09 | 5303388 | 5315598 | Ma09_g08070~ probable pyruvate kinase, cytosolic isozyme~ unknown_gene~ missing_functional_completeness |
| pyruvate kinase | Ma09_p24220.1 | chr09 | 35902688 | 35906649 | Ma09_g24220~ Pyruvate kinase, cytosolic isozyme~ PKM2~ complete |
| pyruvate kinase | Ma10_p04950.1 | chr10 | 15589645 | 15592987 | Ma10_g04950~ Pyruvate kinase, cytosolic isozyme~ PKM2~ complete |
| pyruvate kinase | Ma10_p15480.1 | chr10 | 27553717 | 27562525 | Ma10_g15480~ Putative Pyruvate kinase, cytosolic isozyme~ pkiA~ complete |
| pyruvate kinase | Ma11_p09970.1 | chr11 | 9163687 | 9171501 | Ma11_g09970~ Putative Pyruvate kinase, cytosolic isozyme~ pkiA~ complete |
| 2,3-bisphosphoglycerate-independent phosphoglycerate mutase | Ma04_p10290.1 | chr04 | 7342891 | 7348391 | Ma04_g10290~ 2,3-bisphosphoglycerate-independent phosphoglycerate mutase-like~ unknown_gene~ missing_functional_completeness |
| 2,3-bisphosphoglycerate-independent phosphoglycerate mutase | Ma07_p23360.1 | chr07 | 31035010 | 31043234 | Ma07_g23360~ 2,3-bisphosphoglycerate-independent phosphoglycerate mutase, transcriptvariant X2~unknown_gene~ missing_functional_completeness |
| 2,3-bisphosphoglycerate-independent phosphoglycerate mutase | Ma07_p23360.2 | chr07 | 31035010 | 31043234 | Ma07_g23360~ 2,3-bisphosphoglycerate-independent phosphoglycerate mutase, transcript variant X2~unknown_gene~ missing_functional_completeness |
| 2,3-bisphosphoglycerate-independent phosphoglycerate mutase | Ma07_p23360.3 | chr07 | 31035010 | 31043234 | Ma07_g23360~ 2,3-bisphosphoglycerate-independent phosphoglycerate mutase, transcript variant X2~ unknown_gene~ missing_functional_completeness |

Table S3. *Cont*.

| Gene Name  Used in this  Research | Gene ID in  Banana Genome  Hub | Gene  Location | Start | End | Gene Annotation |
| --- | --- | --- | --- | --- | --- |
| 2,3-bisphosphoglycerate-independent phosphoglycerate mutase | Ma09_p04830.1 | chr09 | 3108253 | 3109803 | Ma09_g04830~ 2,3-bisphosphoglycerate-independent phosphoglycerate mutase~ PGM1~ fragment |
| 2,3-bisphosphoglycerate-independent phosphoglycerate mutase | Ma09_p04840.1 | chr09 | 3111004 | 3113789 | Ma09_g04840~ 2,3-bisphosphoglycerate-independent phosphoglycerate mutase~ PGM1~ fragment |
| 2,3-bisphosphoglycerate-independent phosphoglycerate mutase | Ma10_p29170.1 | chr10 | 35830183 | 35835552 | Ma10_g29170~ 2,3-bisphosphoglycerate-independent phosphoglycerate mutase, transcript variant X2~ unknown_gene~ missing_functional_completeness |
| 2,3-bisphosphoglycerate-independent phosphoglycerate mutase | Ma10_p29170.2 | chr10 | 35830183 | 35835552 | Ma10_g29170~ 2,3-bisphosphoglycerate-independent phosphoglycerate mutase, transcript variant X2~ unknown_gene~ missing_functional_completeness |
| 2,3-bisphosphoglycerate-independent phosphoglycerate mutase | Ma10_p29170.3 | chr10 | 35830183 | 35835552 | Ma10_g29170~ 2,3-bisphosphoglycerate-independent phosphoglycerate mutase, transcript variant X2~ unknown_gene~ missing_functional_completeness |
| 6-phosphofructokinase | Ma01_p05870.1 | chr01 | 4152379 | 4 163421 | Ma01_g05870~ 6-phosphofructokinase 5, chloroplastic~ pfp~ complete |
| 6-phosphofructokinase | Ma01_p15040.1 | chr01 | 10923840 | 10930628 | Ma01_g15040~ 6-phosphofructokinase 5, chloroplastic~ pfp~ complete |
| 6-phosphofructokinase | Ma02_p18360.1 | chr02 | 24871930 | 24876386 | Ma02_g18360~ 6-phosphofructokinase 3~ pfp~ complete |
| 6-phosphofructokinase | Ma04_p01000.1 | chr04 | 885610 | 892035 | Ma04_g01000~ 6-phosphofructokinase 3~ pfp~ complete |
| 6-phosphofructokinase | Ma04_p08280.1 | chr04 | 5948415 | 5954645 | Ma04_g08280~ 6-phosphofructokinase 3~ pfp~ complete |
| 6-phosphofructokinase | Ma04_p15430.1 | chr04 | 11709406 | 11713186 | Ma04_g15430~ 6-phosphofructokinase 3~ pfp~ pseudogene |
| 6-phosphofructokinase | Ma04_p24700.1 | chr04 | 26656659 | 26662692 | Ma04_g24700~ 6-phosphofructokinase 3~ pfp~ complete |
| 6-phosphofructokinase | Ma05_p24880.1 | chr05 | 37017753 | 37020935 | Ma05_g24880~ 6-phosphofructokinase 2~ pfkA1~ complete |
| 6-phosphofructokinase | Ma07_p10560.1 | chr07 | 7890072 | 7894434 | Ma07_g10560~ 6-phosphofructokinase 3~ pfkA~ pseudogene |
| 6-phosphofructokinase | Ma07_p11340.1 | chr07 | 8423633 | 8430537 | Ma07_g11340~ 6-phosphofructokinase 5, chloroplastic~ pfkA2~ complete |
| 6-phosphofructokinase | Ma07_p13520.1 | chr07 | 10169690 | 10173615 | Ma07_g13520~ 6-phosphofructokinase 3~ pfp~ complete |
| 6-phosphofructokinase | Ma09_p18430.1 | chr09 | 17917457 | 17918920 | Ma09_g18430~ 6-phosphofructokinase 2~ pfp~ complete |
| alcohol dehydrogenase | Ma02_p09950.1 | chr02 | 19697137 | 19699288 | Ma02_g09950~ Alcohol dehydrogenase 1~ ADH2~ complete |
| alcohol dehydrogenase | Ma04_p06170.1 | chr04 | 4555258 | 4556681 | Ma04_g06170~ cinnamyl alcohol dehydrogenase 2-like~ unknown_gene~ missing_functional_completeness |
| alcohol dehydrogenase | Ma04_p12960.1 | chr04 | 9804459 | 9806842 | Ma04_g12960~ cinnamyl alcohol dehydrogenase 2-like, transcript variant X2~ unknown_gene~missing_functional_completeness |
| alcohol dehydrogenase | Ma04_p12960.2 | chr04 | 9804459 | 9807069 | Ma04_g12960~ cinnamyl alcohol dehydrogenase 2-like, transcript variant X2~ unknown_gene~ missing_functional_Completeness |
| alcohol dehydrogenase | Ma04_p20820.1 | chr04 | 23534434 | 23538284 | Ma04_g20820~ probable cinnamyl alcohol dehydrogenase 1~ unknown_gene~ missing_functional_completeness |
| alcohol dehydrogenase | Ma04_p25530.1 | chr04 | 27271288 | 27275698 | Ma04_g25530~ Alcohol dehydrogenase class-3~ FDH~ complete |
| alcohol dehydrogenase | Ma04_p31940.1 | chr04 | 32111836 | 32116353 | Ma04_g31940~ Alcohol dehydrogenase-like 6~ ADH3~ complete |
| alcohol dehydrogenase | Ma04_p33020.1 | chr04 | 32856683 | 32858231 | Ma04_g33020~ probable cinnamyl alcohol dehydrogenase 1~ unknown_gene~ missing_functional_completeness |
| alcohol dehydrogenase | Ma04_p36000.1 | chr04 | 34517921 | 34521770 | Ma04_g36000~ Alcohol dehydrogenase-like 3~ ADH2~ complete |
| alcohol dehydrogenase | Ma06_p19900.1 | chr06 | 13988688 | 13990608 | Ma06_g19900~ Alcohol dehydrogenase 3~ ADH~ complete |
| alcohol dehydrogenase | Ma07_p11500.1 | chr07 | 8539962 | 8543179 | Ma07_g11500~ Alcohol dehydrogenase-like 7~ ADH~ fragment |
| alcohol dehydrogenase | Ma07_p11510.1 | chr07 | 8546813 | 8550412 | Ma07_g11510~ Alcohol dehydrogenase-like 7~ Os02g0815500~ complete |

Table S3. *Cont*.

| Gene Name  Used in this  Research | Gene ID in  Banana Genome  Hub | Gene  Location | Start | End | Gene Annotation |
| --- | --- | --- | --- | --- | --- |
| alcohol dehydrogenase | Ma08_p29910.1 | chr08 | 41352852 | 41355359 | Ma08_g29910~ Alcohol dehydrogenase 1~ ADH~ complete |
| alcohol dehydrogenase | Ma09_p08110.1 | chr09 | 5334827 | 5337766 | Ma09_g08110~ Alcohol dehydrogenase 2~ ADH~ complete |
| alcohol dehydrogenase | Ma10_p17990.1 | chr10 | 29138978 | 29140670 | Ma10_g17990~ probable cinnamyl alcohol dehydrogenase 9~ unknown_gene~ missing_functional_completeness |
| alcohol dehydrogenase | Ma11_p07340.1 | chr11 | 5828866 | 5830843 | Ma11_g07340~ Alcohol dehydrogenase 1~ ADH2~ complete |
| aldolase | Ma01_p00110.1 | chr01 | 104577 | 105077 | Ma01_g00110~ putative 4-hydroxy-4-methyl-2-oxoglutarate aldolase 3, transcript variant X1~ unknown_gene~ missing_functional_completeness |
| aldolase | Ma01_p00110.2 | chr01 | 104577 | 105077 | Ma01_g00110~ putative 4-hydroxy-4-methyl-2-oxoglutarate aldolase 3, transcript variant X1~ unknown_gene~ missing_functional_completeness |
| aldolase | Ma01_p14880.1 | chr01 | 10840654 | 10842684 | Ma01_g14880~ phospho-2-dehydro-3-deoxyheptonate aldolase 2, chloroplastic-like~ unknown_gene~ missing_functional_completeness |
| aldolase | Ma02_p14050.1 | chr02 | 22205971 | 22209128 | Ma02_g14050~ probable low-specificity L-threonine aldolase 1~ unknown_gene~ missing_functional_completeness |
| aldolase | Ma03_p11730.1 | chr03 | 9104927 | 9107389 | Ma03_g11730~ fructose-bisphosphate aldolase, cytoplasmic isozyme 1-like~ unknown_gene~ missing_functional_completeness |
| aldolase | Ma03_p15050.1 | chr03 | 14821573 | 14822079 | Ma03_g15050~ putative 4-hydroxy-4-methyl-2-oxoglutarate aldolase 2~ unknown_gene~ missing_functional_completeness |
| aldolase | Ma04_p05300.1 | chr04 | 3961099 | 3961605 | Ma04_g05300~ putative 4-hydroxy-4-methyl-2-oxoglutarate aldolase 1, transcript variant X1~ unknown_gene~ missing_functional_completeness |
| aldolase | Ma04_p05300.2 | chr04 | 3961099 | 3961605 | Ma04_g05300~ putative 4-hydroxy-4-methyl-2-oxoglutarate aldolase 1, transcript variant X1~unknown_gene~ missing_functional_completeness |
| aldolase | Ma04_p11800.1 | chr04 | 8414330 | 8414836 | Ma04_g11800~ putative 4-hydroxy-4-methyl-2-oxoglutarate aldolase 2~ unknown_gene~ missing_functional_completeness |
| aldolase | Ma04_p35430.1 | chr04 | 34216197 | 34220389 | Ma04_g35430~ phospho-2-dehydro-3-deoxyheptonate aldolase 1, chloroplastic-like~ unknown_gene~ missing_functional_completeness |
| aldolase | Ma05_p19700.1 | chr05 | 28239181 | 28240038 | Ma05_g19700~ Phospho-2-dehydro-3-deoxyheptonate aldolase 1, chloroplastic~ DAHPS1~ fragment |
| aldolase | Ma05_p22300.1 | chr05 | 34032047 | 34033642 | Ma05_g22300~ fructose-bisphosphate aldolase 1, chloroplastic~ unknown_gene~ missing_functional_completeness |
| aldolase | Ma05_p27790.1 | chr05 | 38933448 | 38936469 | Ma05_g27790~ fructose-bisphosphate aldolase cytoplasmic isozyme-like~ unknown_gene~ missing_functional_completeness |
| aldolase | Ma06_p11050.1 | chr06 | 7762827 | 7766709 | Ma06_g11050~ fructose-bisphosphate aldolase 1,chloroplastic-like~ unknown_gene~missing_functional_completeness |
| aldolase | Ma06_p19280.1 | chr06 | 13242366 | 13244002 | Ma06_g19280~ fructose-bisphosphate aldolase, chloroplastic~ unknown_gene~ missing_functional_completeness |
| aldolase | Ma06_p21500.1 | chr06 | 15729162 | 15731466 | Ma06_g21500~ dihydroneopterin aldolase-like, transcript variant X2~ unknown_gene~ missing_functional_completeness |
| aldolase | Ma06_p21500.2 | chr06 | 15729162 | 15731466 | Ma06_g21500~ dihydroneopterin aldolase-like, transcript variant X2~ unknown_gene~missing_functional_completeness |

Table S3. *Cont*.

| Gene Name  Used in this  Research | Gene ID in  Banana Genome  Hub | Gene  Location | Start | End | Gene Annotation |
| --- | --- | --- | --- | --- | --- |
| aldolase | Ma07_p22590.2 | chr07 | 30469232 | 30473457 | Ma07_g22590~ [Fructose-bisphosphate aldolase]-lysine N-methyltransferase, chloroplastic-like,transcript variant X2~unknown_gene~missing_functional_completeness |
| aldolase | Ma07_p22620.1 | chr07 | 30495722 | 30500848 | Ma07_g22620~ [Fructose-bisphosphate aldolase]-lysine N-methyltransferase, chloroplastic-like~ unknown_gene~ missing_functional_completeness |
| aldolase | Ma08_p01140.1 | chr08 | 1061124 | 1063311 | Ma08_g01140~ fructose-bisphosphate aldolase cytoplasmic isozyme~ unknown_gene~ missing_functional_completeness |
| aldolase | Ma08_p07480.1 | chr08 | 5120544 | 5123633 | Ma08_g07480~ fructose-bisphosphate aldolase cytoplasmic isozyme-like, transcript variant X1~ unknown_gene~ missing_functional_completeness |
| aldolase | Ma08_p07480.2 | chr08 | 5120544 | 5123633 | Ma08_g07480~ fructose-bisphosphate aldolase cytoplasmic isozyme-like, transcript variant X1~ unknown_gene~ missing_functional_completeness |
| aldolase | Ma08_p08700.1 | chr08 | 6148508 | 6151704 | Ma08_g08700~ fructose-bisphosphate aldolase cytoplasmic isozyme-like~ unknown_gene~ missing_functional_completeness |
| aldolase | Ma08_p16120.1 | chr08 | 17083244 | 17085611 | Ma08_g16120~ fructose-bisphosphate aldolase, chloroplastic-like~ unknown_gene~missing_functional_completeness |
| aldolase | Ma08_p16810.1 | chr08 | 20778782 | 20781999 | Ma08_g16810~ fructose-bisphosphate aldolase 1, chloroplastic-like~ unknown_gene~ missing_functional_completeness |
| aldolase | Ma08_p29530.1 | chr08 | 41090337 | 41103884 | Ma08_g29530~ 2-dehydro-3-deoxyphosphooctonate aldolase~ unknown_gene~ missing_functional_completeness |
| aldolase | Ma09_p29310.1 | chr09 | 39788075 | 39790321 | Ma09_g29310~ dihydroneopterin aldolase-like~ unknown_gene~ missing_functional_completeness |
| aldolase | Ma09_p31410.1 | chr09 | 41149275 | 41151595 | Ma09_g31410~ dihydroneopterin aldolase-like, transcript variant X1~ unknown_gene~ missing_functional_completeness |
| aldolase | Ma09_p31410.2 | chr09 | 41149275 | 41169523 | Ma09_g31410~ dihydroneopterin aldolase-like, transcript variant X1~ unknown_gene~ missing_functional_completeness |
| aldolase | Ma09_p31420.1 | chr09 | 41155528 | 41157762 | Ma09_g31420~ dihydroneopterin aldolase-like~ unknown_gene~ missing_functional_completeness |
| aldolase | Ma10_p10680.1 | chr10 | 24499808 | 24500398 | Ma10_g10680~ fructose-bisphosphate aldolase, chloroplastic-like~ unknown_gene~ missing_functional_completeness |
| aldolase | Ma10_p24640.1 | chr10 | 33139892 | 33143015 | Ma10_g24640~ phospho-2-dehydro-3-deoxyheptonate aldolase 2, chloroplastic-like~  unknown_gene~ missing_functional_completeness |
| aldolase | Ma10_p30330.1 | chr10 | 36652311 | 36655242 | Ma10_g30330~ phospho-2-dehydro-3-deoxyheptonate aldolase 2, chloroplastic-like~  unknown_gene~ missing_functional_completeness |
| aldolase | Ma11_p03650.1 | chr11 | 2719211 | 2725378 | Ma11_g03650~ probable transaldolase~ unknown_gene~ missing_functional_completeness |
| aldolase | Ma11_p22910.1 | chr11 | 26483024 | 26495256 | Ma11_g22910~ [Fructose-bisphosphate aldolase]-lysine N-methyltransferase, chloroplastic, transcript variant X2~ unknown_gene~ missing_functional_completeness |
| aldolase | Ma11_p22910.2 | chr11 | 26483024 | 26495256 | Ma11_g22910~ [Fructose-bisphosphate aldolase]-lysine N-methyltransferase, chloroplastic,transcript variant X2~ unknown_gene~ missing_functional_completeness |
| aldolase | Ma11_p25030.1 | chr11 | 27796560 | 27796955 | Ma11_g25030~ Fructose-bisphosphate aldolase cytoplasmic isozyme~ FBA~ fragment |
| aldolase | Ma11_p25040.1 | chr11 | 27796964 | 27800885 | Ma11_g25040~ Fructose-bisphosphate aldolase cytoplasmic isozyme~ FBA~ fragment |

Table S3. *Cont*.

| Gene Name  Used in this  Research | Gene ID in  Banana Genome  Hub | Gene  Location | Start | End | Gene Annotation |
| --- | --- | --- | --- | --- | --- |
| aldose 1-epimerase | Ma05_p25750.1 | chr05 | 37592782 | 37595266 | Ma05_g25750~ aldose 1-epimerase-like~ unknown_gene~ missing_functional_completeness |
| aldose 1-epimerase | Ma06_p23880.1 | chr06 | 22056975 | 22059413 | Ma06_g23880~ aldose 1-epimerase-like~ unknown_gene~ missing_functional_completeness |
| aldose 1-epimerase | Ma08_p34760.1 | chr08 | 44752873 | 44755226 | Ma08_g34760~ aldose 1-epimerase-like~ unknown_gene~ missing_functional_completeness |
| aldose 1-epimerase | Ma09_p11280.1 | chr09 | 7625588 | 7628750 | Ma09_g11280~ aldose 1-epimerase-like~ unknown_gene~ missing_functional_completeness |
| dihydrolipoyl dehydrogenase | Ma01_p11770.1 | chr01 | 8534387 | 8546449 | Ma01_g11770~ dihydrolipoyl dehydrogenase 1, chloroplastic-like~ unknown_gene~ missing_functional_completeness |
| dihydrolipoyl dehydrogenase | Ma02_p03980.1 | chr02 | 15413307 | 15417984 | Ma02_g03980~ dihydrolipoyl dehydrogenase, mitochondrial-like~ unknown_gene~ missing_functional_completeness |
| dihydrolipoyl dehydrogenase | Ma03_p12420.1 | chr03 | 9579211 | 9592446 | Ma03_g12420~ dihydrolipoyl dehydrogenase 2, chloroplastic-like~ unknown_gene~ missing_functional_completeness |
| dihydrolipoyl dehydrogenase | Ma08_p22830.1 | chr08 | 36315704 | 36316032 | Ma08_g22830~ Putative Dihydrolipoyl dehydrogenase~ lpdA~ fragment |
| dihydrolipoyl dehydrogenase | Ma11_p12560.1 | chr11 | 16469206 | 16473715 | Ma11_g12560~ dihydrolipoyl dehydrogenase, mitochondrial-like~ unknown_gene~ missing_functional_completeness |
| dihydrolipoyllysine-residue acetyltransferase | Ma03_p07300.1 | chr03 | 5092566 | 5096206 | Ma03_g07300~ dihydrolipoyllysine-residue acetyltransferase component of pyruvate dehydrogenase complex, mitochondrial~ unknown_gene~missing_functional_completeness |
| dihydrolipoyllysine-residue acetyltransferase | Ma03_p18460.1 | chr03 | 24089928 | 24095495 | Ma03_g18460~ dihydrolipoyllysine-residue acetyltransferase component of pyruvate dehydrogenase complex, mitochondrial~ unknown_gene missing_functional_completeness |
| dihydrolipoyllysine-residue acetyltransferase | Ma08_p17990.1 | chr08 | 27759660 | 27793869 | Ma08_g17990~ dihydrolipoyllysine-residue acetyltransferase component 1 of pyruvate dehydrogenase complex, mitochondrial, transcript variant X1~ unknown_gene~ missing_functional_completeness |
| dihydrolipoyllysine-residue acetyltransferase | Ma08_p17990.2 | chr08 | 27759660 | 27796760 | Ma08_g17990~ dihydrolipoyllysine-residue acetyltransferase component 1 of pyruvate dehydrogenase complex, mitochondrial, transcript variant X1~ unknown_gene~ missing_functional_completeness |
| dihydrolipoyllysine-residue acetyltransferase | Ma08_p17990.3 | chr08 | 27759660 | 27796760 | Ma08_g17990~ dihydrolipoyllysine-residue acetyltransferase component 1 of pyruvate dehydrogenase complex, mitochondrial, transcript variant X1~ unknown_gene~ missing_functional_completeness |
| dihydrolipoyllysine-residue acetyltransferase | Ma09_p08190.1 | chr09 | 5393185 | 5396657 | Ma09_g08190~ dihydrolipoyllysine-residue acetyltransferase component of pyruvate dehydrogenase complex, mitochondrial-like~ unknown_gene~missing_functional_completeness |
| dihydrolipoyllysine-residue acetyltransferase | Ma10_p09080.1 | chr10 | 23277888 | 23294714 | Ma10_g09080~ dihydrolipoyllysine-residue acetyltransferase component 3 of pyruvate dehydrogenase omplex, mitochondrial-like~ unknown_gene~missing_functional_completeness |

Table S3. *Cont*.

| Gene Name  Used in this  Research | Gene ID in  Banana Genome  Hub | Gene  Location | Start | End | Gene Annotation |
| --- | --- | --- | --- | --- | --- |
| dihydrolipoyllysine-residue acetyltransferase | Ma08_p17990.2 | chr08 | 27759660 | 27796760 | Ma08_g17990~ dihydrolipoyllysine-residue acetyltransferase component 1 of pyruvate dehydrogenase omplex, mitochondrial, transcript variant X1~ unknown_genes |
| dihydrolipoyllysine-residue acetyltransferase | Ma03_p07300.1 | chr03 | 5092566 | 5096206 | Ma03_g07300~ dihydrolipoyllysine-residue acetyltransferase component of pyruvate dehydrogenase omplex, mitochondrial~ unknown_gene~missing_functional_completeness |
| dihydrolipoyllysine-residue acetyltransferase | Ma08_p17990.1 | chr08 | 27759660 | 27793869 | Ma08_g17990~ dihydrolipoyllysine-residue acetyltransferase component 1 of pyruvate dehydrogenase complex, mitochondrial, transcript variant X1~ unknown_gene~ missing_functional_completeness |
| dihydrolipoyllysine-residue acetyltransferase | Ma08_p17990.3 | chr08 | 27759660 | 27796760 | Ma08_g17990~ dihydrolipoyllysine-residue acetyltransferase component 1 of pyruvate dehydrogenase complex, mitochondrial, transcript variant X1~ unknown_gene~ missing_functional_completeness |
| dihydrolipoyllysine-residue acetyltransferase | Ma03_p18460.1 | chr03 | 24089928 | 24095495 | Ma03_g18460~ dihydrolipoyllysine-residue acetyltransferase component of pyruvate dehydrogenase complex, mitochondrial~ unknown_gene~ missing_functional_completeness |
| fructose-bisphosphate aldolase | Ma03_p11730.1 | chr03 | 9104927 | 9107389 | Ma03_g11730~ fructose-bisphosphate aldolase, cytoplasmic isozyme 1-like~ unknown_  gene~ missing_functional_completeness |
| fructose-bisphosphate aldolase | Ma05_p22300.1 | chr05 | 34032047 | 34033642 | Ma05_g22300~ fructose-bisphosphate aldolase 1, chloroplastic~ unknown_gene~ missing_functional_completeness |
| fructose-bisphosphate aldolase | Ma05_p27790.1 | chr05 | 38933448 | 38936469 | Ma05_g27790~ fructose-bisphosphate aldolase cytoplasmic isozyme-like~ unknown_gene~missing_functional_completeness |
| fructose-bisphosphate aldolase | Ma06_p11050.1 | chr06 | 7762827 | 7766709 | Ma06_g11050~ fructose-bisphosphate aldolase 1, chloroplastic-like~ unknown_gene~ missing_functional_completeness |
| fructose-bisphosphate aldolase | Ma06_p19280.1 | chr06 | 13242366 | 13244002 | Ma06_g19280~ fructose-bisphosphate aldolase, chloroplastic~ unknown_gene~ missing_functional_completeness |
| fructose-bisphosphate aldolase | Ma07_p22590.1 | chr07 | 30469232 | 30473457 | Ma07_g22590~ [Fructose-bisphosphate aldolase]-lysine N-methyltransferase,  chloroplastic-like, transcript variant X2~ unknown_gene~ missing_functional_completeness |
| fructose-bisphosphate aldolase | Ma07_p22590.2 | chr07 | 30469232 | 30473457 | Ma07_g22590~ [Fructose-bisphosphate aldolase]-lysine N-methyltransferase,  chloroplastic-like, transcript variant X2~ unknown_gene~ missing_functional_completeness |
| fructose-bisphosphate aldolase | Ma07_p22620.1 | chr07 | 30495722 | 30500848 | Ma07_g22620~ [Fructose-bisphosphate aldolase]-lysine N-methyltransferase,  chloroplastic-like~ unknown_gene~ missing_functional_completenes |
| fructose-bisphosphate aldolase | Ma08_p01140.1 | chr08 | 1061124 | 1063311 | Ma08_g01140~ fructose-bisphosphate aldolase cytoplasmic isozyme~ unknown_  gene~ missing_functional_completeness |
| fructose-bisphosphate aldolase | Ma08_p07480.1 | chr08 | 5120544 | 5123633 | Ma08_g07480~ fructose-bisphosphate aldolase cytoplasmic isozyme-like,  transcript variant X1~ unknown_gene~ missing_functional_completeness |
| fructose-bisphosphate aldolase | Ma08_p07480.2 | chr08 | 5120544 | 5123633 | Ma08_g07480~ fructose-bisphosphate aldolase cytoplasmic isozyme-like,  transcript variant X1~ unknown_gene~ missing_functional_completeness |

Table S3. *Cont*.

| Gene Name  Used in this  Research | Gene ID in  Banana Genome  Hub | Gene  Location | Start | End | Gene Annotation |
| --- | --- | --- | --- | --- | --- |
| fructose-bisphosphate aldolase | Ma08_p08700.1 | chr08 | 6148508 | 6151704 | Ma08_g08700~ fructose-bisphosphate aldolase cytoplasmic isozyme-like~ unknown_gene~ missing_functional_completeness |
| fructose-bisphosphate aldolase | Ma08_p16120.1 | chr08 | 17083244 | 17085611 | Ma08_g16120~ fructose-bisphosphate aldolase, chloroplastic-like~ unknown_gene~ missing_functional_completeness |
| fructose-bisphosphate aldolase | Ma08_p16810.1 | chr08 | 20778782 | 20781999 | Ma08_g16810~ fructose-bisphosphate aldolase 1, chloroplastic-like~ unknown_gene~missing_functional_completeness |
| fructose-bisphosphate aldolase | Ma10_p10680.1 | chr10 | 24499808 | 24500398 | Ma10_g10680~ fructose-bisphosphate aldolase, chloroplastic-like~ unknown_gene~missing_functional_completeness |
| fructose-bisphosphate aldolase | Ma11_p22910.1 | chr11 | 26483024 | 26495256 | Ma11_g22910~ [Fructose-bisphosphate aldolase]-lysine N-methyltransferase,  chloroplastic, transcript variant X2~ unknown_gene~ missing_functional_completeness |
| fructose-bisphosphate aldolase | Ma11_p22910.2 | chr11 | 26483024 | 26495256 | Ma11_g22910~ [Fructose-bisphosphate aldolase]-lysine N-methyltransferase,  chloroplastic, transcript variant X2~ unknown_gene~ missing_functional_completeness |
| fructose-bisphosphate aldolase | Ma11_p25030.1 | chr11 | 27796560 | 27796955 | Ma11_g25030~ Fructose-bisphosphate aldolase cytoplasmic isozyme~ FBA~ fragment |
| fructose-bisphosphate aldolase | Ma11_p25040.1 | chr11 | 27796964 | 27800885 | Ma11_g25040~ Fructose-bisphosphate aldolase cytoplasmic isozyme~ FBA~ fragment |
| glucose-6-phosphate isomerase | Ma07_p08070.1 | chr07 | 6016867 | 6025844 | Ma07_g08070~ glucose-6-phosphate isomerase, cytosolic, transcript variant X1~ unknown_gene~missing_F unctional_completeness |
| glucose-6-phosphate isomerase | Ma07_p08070.2 | chr07 | 6016867 | 6025844 | Ma07_g08070~ glucose-6-phosphate isomerase, cytosolic, transcript variant X1~ unknown_gene~missing_functional_completeness |
| glyceraldehyde-3-phosphate dehydrogenase | Ma01_p11940.1 | chr01 | 8654885 | 8655250 | Ma01_g11940~ glyceraldehyde-3-phosphate dehydrogenase, testis-specific~ unknown_gene~ missing_functional_completeness |
| glyceraldehyde-3-phosphate dehydrogenase | Ma02_p04500.1 | chr02 | 15907679 | 15908105 | Ma02_g04500~ Glyceraldehyde-3-phosphate dehydrogenase~ gapdh~ fragment |
| glyceraldehyde-3-phosphate dehydrogenase | Ma02_p04510.1 | chr02 | 15908123 | 15908890 | Ma02_g04510~ Glyceraldehyde-3-phosphate dehydrogenase, cytosolic~ GAPC~ fragment |
| glyceraldehyde-3-phosphate dehydrogenase | Ma05_p00210.1 | chr05 | 153041 | 158138 | Ma05_g00210~ glyceraldehyde-3-phosphate dehydrogenase GAPCP2, chloroplastic-like~ unknown_gene~ missing_functional_completeness |
| glyceraldehyde-3-phosphate dehydrogenase | Ma05_p17770.1 | chr05 | 21614196 | 21615064 | Ma05_g17770~ glyceraldehyde-3-phosphate dehydrogenase, putative, expressed~ GAPC1~fragment |
| glyceraldehyde-3-phosphate dehydrogenase | Ma05_p27700.1 | chr05 | 38887130 | 38891852 | Ma05_g27700~ glyceraldehyde-3-phosphate dehydrogenase 2, cytosolic~ unknown_gene~ missing_functional_completeness |
| glyceraldehyde-3-phosphate dehydrogenase | Ma06_p01470.1 | chr06 | 1190774 | 1194336 | Ma06_g01470~ glyceraldehyde-3-phosphate dehydrogenase 2, cytosolic-like~ unknown_gene~ missing_functional_completeness |
| glyceraldehyde-3-phosphate dehydrogenase | Ma06_p03680.1 | chr06 | 2676010 | 2681182 | Ma06_g03680~ NADP-dependent glyceraldehyde-3-phosphate dehydrogenase~ unknown_gene~ missing_functional_completeness |
| glyceraldehyde-3-phosphate dehydrogenase | Ma06_p04220.1 | chr06 | 3061274 | 3061714 | Ma06_g04220~ glyceraldehyde-3-phosphate dehydrogenase, testis-specific-like~ unknown_gene~ missing_functional_completeness |
| glyceraldehyde-3-phosphate dehydrogenase | Ma06_p17760.1 | chr06 | 12055149 | 12057250 | Ma06_g17760~ glyceraldehyde-3-phosphate dehydrogenase A, chloroplastic-like~ unknown_gene~missing_functional_completeness |

Table S3. *Cont*.

| Gene Name  Used in this  Research | Gene ID in  Banana Genome  Hub | Gene  Location | Start | End | Gene Annotation |
| --- | --- | --- | --- | --- | --- |
| glyceraldehyde-3-phosphate dehydrogenase | Ma07_p03670.1 | chr07 | 2807539 | 2814308 | Ma07_g03670~ glyceraldehyde-3-phosphate dehydrogenase GAPCP2, chloroplastic-like~ unknown_gene~missing_functional_completeness |
| glyceraldehyde-3-phosphate dehydrogenase | Ma07_p20820.1 | chr07 | 28829713 | 28832911 | Ma07_g20820~ NADP-dependent glyceraldehyde-3-phosphate dehydrogenase-like~ unknown_gene~missing_functional_completeness |
| glyceraldehyde-3-phosphate dehydrogenase | Ma08_p33830.1 | chr08 | 44031420 | 44037840 | Ma08_g33830~ glyceraldehyde-3-phosphate dehydrogenase GAPCP1, chloroplastic-like~ unknown_gene~missing_functional_completeness |
| glyceraldehyde-3-phosphate dehydrogenase | Ma09_p02110.1 | chr09 | 1536946 | 1540648 | Ma09_g02110~ glyceraldehyde-3-phosphate dehydrogenase 2, cytosolic-like~ unknown_gene~missing_functional_completeness |
| glyceraldehyde-3-phosphate dehydrogenase | Ma10_p12550.1 | chr10 | 25599210 | 25602699 | Ma10_g12550~ glyceraldehyde-3-phosphate dehydrogenase A, chloroplastic~ unknown_gene~missing_functional_completeness |
| glyceraldehyde-3-phosphate dehydrogenase | Ma11_p01390.1 | chr11 | 977738 | 981496 | Ma11_g01390~ glyceraldehyde-3-phosphate dehydrogenase B, chloroplastic-like~ unknown_gene~missing_functional_completeness |
| glyceraldehyde-3-phosphate dehydrogenase | Ma11_p08300.1 | chr11 | 6609958 | 6613558 | Ma11_g08300~ glyceraldehyde-3-phosphate dehydrogenase 2, cytosolic-like~ unknown_gene~missing_functional_completeness |
| glyceraldehyde-3-phosphate dehydrogenase | Ma11_p17540.1 | chr11 | 22844667 | 22848537 | Ma11_g17540~ glyceraldehyde-3-phosphate dehydrogenase 2, cytosolic-like~ unknown_gene~ missing_functional_completeness |
| glyceraldehyde-3-phosphate dehydrogenase | Ma11_p20650.1 | chr11 | 25051336 | 25053582 | Ma11_g20650~ glyceraldehyde-3-phosphate dehydrogenase B, chloroplastic, transcript variant X2~unknown_gene~ missing_functional_completeness |
| glyceraldehyde-3-phosphate dehydrogenase | Ma11_p20650.2 | chr11 | 25051336 | 25053582 | Ma11_g20650~ glyceraldehyde-3-phosphate dehydrogenase B, chloroplastic, transcript variant X2~ unknown_gene~ missing_functional_completeness |
| hexokinase | Ma01_p05010.1 | chr01 | 3499938 | 3509769 | Ma01_g05010~ hexokinase-3-like~ unknown_gene~ missing_functional_completeness |
| hexokinase | Ma03_p16610.1 | chr03 | 19175906 | 19181474 | Ma03_g16610~ hexokinase-2-like~ unknown_gene~ missing_functional_completeness |
| hexokinase | Ma03_p32880.1 | chr03 | 34485478 | 34490214 | Ma03_g32880~ hexokinase-2-like~ unknown_gene~ missing_functional_completeness |
| hexokinase | Ma06_p07130.1 | chr06 | 5092760 | 5095193 | Ma06_g07130~ Hexokinase-2~ HXK1~ missing_completeness |
| hexokinase | Ma06_p28110.1 | chr06 | 29880977 | 29888258 | Ma06_g28110~ hexokinase-2-like~ unknown_gene~ missing_functional_completeness |
| hexokinase | Ma08_p09550.1 | chr08 | 6912537 | 6917118 | Ma08_g09550~ Hexokinase-3~ HXK3~ complete |
| hexokinase | Ma08_p09560.1 | chr08 | 6923759 | 6924034 | Ma08_g09560~ Hexokinase-1~ HXK1~ fragment |
| hexokinase | Ma08_p28220.1 | chr08 | 40234415 | 40240028 | Ma08_g28220~ hexokinase-2-like~ unknown_gene~ missing_functional_completeness |
| hexokinase | Ma08_p29950.1 | chr08 | 41374068 | 41381741 | Ma08_g29950~ Hexokinase-3~ HXK1~ missing_completeness |
| hexokinase | Ma09_p05860.1 | chr09 | 3787033 | 3789015 | Ma09_g05860~ Putative Hexokinase-2~ HXK2~ fragment |
| hexokinase | Ma11_p05730.1 | chr11 | 4414787 | 4423529 | Ma11_g05730~ hexokinase-3-like, transcript variant X2~ unknown_gene~ missing_functional_completeness |
| hexokinase | Ma11_p05730.2 | chr11 | 4414787 | 4423529 | Ma11_g05730~ hexokinase-3-like, transcript variant X2~ unknown_gene~ missing_functional_completeness |
| hexokinase | Ma11_p05730.3 | chr11 | 4414787 | 4423529 | Ma11_g05730~ hexokinase-3-like, transcript variant X2~ unknown_gene~ missing_functional_completeness |
| hexokinase | Ma11_p05730.4 | chr11 | 4414787 | 4423529 | Ma11_g05730~ hexokinase-3-like, transcript variant X2~ unknown_gene~ missing_functional_completeness |

Table S3. *Cont*.

| Gene Name  Used in this  Research | Gene ID in  Banana Genome  Hub | Gene  Location | Start | End | Gene Annotation |
| --- | --- | --- | --- | --- | --- |
| Phosphoenolpyruvate carboxykinase | Ma04_p27480.1 | chr04 | 28655157 | 28662444 | Ma04_g27480~ phosphoenolpyruvate carboxykinase [ATP]-like~ unknown_gene~ missing_functional_completeness |
| phosphoenolpyruvate carboxykinase | Ma04_p28650.1 | chr04 | 29668523 | 29671758 | Ma04_g28650~ phosphoenolpyruvate carboxykinase [ATP]-like~ unknown_gene~ missing_functional_completeness |
| phosphoenolpyruvate carboxykinase | Ma08_p14790.1 | chr08 | 14730212 | 14733553 | Ma08_g14790~ phosphoenolpyruvate carboxykinase [ATP]-like, transcript variant X2~ unknown_gene~ missing_functional_completeness |
| phosphoenolpyruvate carboxykinase | Ma08_p14790.2 | chr08 | 14730212 | 14734274 | Ma08_g14790~ phosphoenolpyruvate carboxykinase [ATP]-like, transcript variant X2~ unknown_gene~missing_functional_completeness |
| phosphoglucomutase | Ma02_p08630.1 | chr02 | 18912151 | 18920306 | Ma02_g08630~ phosphoglucomutase, cytoplasmic 2-like~ unknown_gene~ missing_functional_completeness |
| phosphoglucomutase | Ma08_p15070.1 | chr08 | 15015087 | 15022457 | Ma08_g15070~ phosphoglucomutase, chloroplastic~ unknown_gene~ missing_functional_completeness |
| phosphoglucomutase | Ma08_p31340.1 | chr08 | 42285583 | 42294687 | Ma08_g31340~ phosphoglucomutase, cytoplasmic 2~ unknown_gene~ missing_functional_completeness |
| pyruvate decarboxylase | Ma01_p22480.1 | chr01 | 25104475 | 25106718 | Ma01_g22480~ Pyruvate decarboxylase isozyme 1~ PDC3~ complete |
| pyruvate decarboxylase | Ma03_p09880.1 | chr03 | 7352328 | 7354702 | Ma03_g09880~ Pyruvate decarboxylase isozyme 2~ PDC3~ complete |
| pyruvate decarboxylase | Ma04_p16540.1 | chr04 | 15944615 | 15948829 | Ma04_g16540~ Pyruvate decarboxylase isozyme 1~ PDC3~ complete |
| pyruvate decarboxylase | Ma05_p07150.1 | chr05 | 5209629 | 5211926 | Ma05_g07150~ Pyruvate decarboxylase isozyme 2~ PDC3~ complete |
| pyruvate decarboxylase | Ma05_p30490.1 | chr05 | 40880480 | 40882769 | Ma05_g30490~ Pyruvate decarboxylase isozyme 2~ PDC3~ complete |
| pyruvate decarboxylase | Ma07_p25850.1 | chr07 | 32821239 | 32825545 | Ma07_g25850~ Putative Pyruvate decarboxylase isozyme 1~ PDC3~ pseudogene |
| pyruvate decarboxylase | Ma11_p23090.1 | chr11 | 26585664 | 26587913 | Ma11_g23090~ Pyruvate decarboxylase isozyme 2~ unknown_gene~ complete |
| pyruvate dehydrogenase E1 component | Ma01_p11600.1 | chr01 | 8385865 | 8391696 | Ma01_g11600~ pyruvate dehydrogenase E1 component subunit alpha-1, mitochondrial-like~ unknown_gene~ missing_functional_completeness |
| pyruvate dehydrogenase E1 component | Ma02_p14810.1 | chr02 | 22746876 | 22747064 | Ma02_g14810~ Pyruvate dehydrogenase E1 component subunit beta~ pdhB~ fragment |
| pyruvate dehydrogenase E1 component | Ma04_p26730.1 | chr04 | 28099280 | 28099432 | Ma04_g26730~ Pyruvate dehydrogenase E1 component subunit alpha-1, mitochondrial~ At1g59900~ fragment |
| pyruvate dehydrogenase E1 component | Ma04_p26740.1 | chr04 | 28099493 | 28102926 | Ma04_g26740~ Pyruvate dehydrogenase E1 component subunit alpha-2, mitochondrial~ IAR4~ fragment |
| pyruvate dehydrogenase E1 component | Ma04_p26750.1 | chr04 | 28103066 | 28103621 | Ma04_g26750~ Pyruvate dehydrogenase E1 component subunit alpha-1, mitochondrial~ At1g59900~ fragment |
| pyruvate dehydrogenase E1 component | Ma04_p38050.1 | chr04 | 35740777 | 35742096 | Ma04_g38050~ Pyruvate dehydrogenase E1 component subunit beta~ pdhB~ complete |
| pyruvate dehydrogenase E1 component | Ma05_p02480.1 | chr05 | 1594148 | 1597888 | Ma05_g02480~ pyruvate dehydrogenase E1 component subunit beta~ unknown_gene~ missing_functional_completeness |
| pyruvate dehydrogenase E1 component | Ma05_p21130.1 | chr05 | 32832959 | 32835903 | Ma05_g21130~ pyruvate dehydrogenase E1 component subunit alpha-3, chloroplastic-like~ unknown_gene~missing_functional_completeness |
| pyruvate dehydrogenase E1 component | Ma05_p27260.1 | chr05 | 38628305 | 38634533 | Ma05_g27260~ pyruvate dehydrogenase E1 component subunit beta-1, mitochondrial~ unknown_gene~ missing_functional_completeness |

Table S3. *Cont*.

| Gene Name  Used in this  Research | Gene ID in  Banana Genome  Hub | Gene  Location | Start | End | Gene Annotation |
| --- | --- | --- | --- | --- | --- |
| pyruvate dehydrogenase E1 component | Ma07_p05600.1 | chr07 | 4053515 | 4058677 | Ma07_g05600~ pyruvate dehydrogenase E1 component subunit alpha-1, mitochondrial-like~ unknown_gene~ missing_functional_completeness |
| pyruvate dehydrogenase E1 component | Ma08_p08880.1 | chr08 | 6313328 | 6316668 | Ma08_g08880~ Pyruvate dehydrogenase E1 component subunit beta, mitochondrial~ PDH2~ fragment |
| pyruvate dehydrogenase E1 component | Ma10_p29610.1 | chr10 | 36141963 | 36150350 | Ma10_g29610~ pyruvate dehydrogenase E1 component subunit alpha-1, mitochondrial-like~ unknown_gene~ missing_functional_completeness |
| pyruvate dehydrogenase E1 component | Ma11_p20960.1 | chr11 | 25296643 | 25299288 | Ma11_g20960~ pyruvate dehydrogenase E1 component subunit alpha-3, chloroplastic-like~ unknown_gene~ missing_functional_completeness |
| pyruvate kinase | Ma01_p17610.1 | chr01 | 12895513 | 12897766 | Ma01_g17610~ Pyruvate kinase~ PKM2~ complete |
| pyruvate kinase | Ma02_p05810.1 | chr02 | 16999492 | 17002657 | Ma02_g05810~ Pyruvate kinase, cytosolic isozyme~ pkiA~ complete |
| pyruvate kinase | Ma02_p19270.1 | chr02 | 25492410 | 25500153 | Ma02_g19270~ Pyruvate kinase isozyme A, chloroplastic~ pyk~ complete |
| pyruvate kinase | Ma02_p22100.1 | chr02 | 27533876 | 27542886 | Ma02_g22100~ Putative Pyruvate kinase, cytosolic isozyme~ pkiA~ complete |
| pyruvate kinase | Ma03_p28290.1 | chr03 | 31411983 | 31416934 | Ma03_g28290~ Pyruvate kinase, cytosolic isozyme~ PKM2~ complete |
| pyruvate kinase | Ma03_p33150.1 | chr03 | 34616911 | 34621334 | Ma03_g33150~ Putative Pyruvate kinase isozyme G, chloroplastic~ pyk~ remnant |
| pyruvate kinase | Ma04_p27720.1 | chr04 | 28890595 | 28898105 | Ma04_g27720~ Pyruvate kinase isozyme A, chloroplastic~ pyk1~ complete |
| pyruvate kinase | Ma04_p28340.1 | chr04 | 29401125 | 29408200 | Ma04_g28340~ Pyruvate kinase isozyme G, chloroplastic~ pyk~ complete |
| pyruvate kinase | Ma04_p35050.1 | chr04 | 34001188 | 34005980 | Ma04_g35050~ Pyruvate kinase isozyme A, chloroplastic~ pyk~ complete |
| pyruvate kinase | Ma04_p35390.1 | chr04 | 34191276 | 34197170 | Ma04_g35390~ Pyruvate kinase isozyme G, chloroplastic~ pyk~ complete |
| pyruvate kinase | Ma05_p20410.1 | chr05 | 32100667 | 32101999 | Ma05_g20410~ Pyruvate kinase isozyme G, chloroplastic~ pkiA~ remnant |
| pyruvate kinase | Ma05_p20420.1 | chr05 | 32102214 | 32102986 | Ma05_g20420~ Pyruvate kinase isozyme G, chloroplastic~ pykF~ remnant |
| pyruvate kinase | Ma05_p20430.1 | chr05 | 32105103 | 32105378 | Ma05_g20430~ Pyruvate kinase~ pyk~ remnant |
| pyruvate kinase | Ma05_p30050.1 | chr05 | 40583885 | 40586993 | Ma05_g30050~ Pyruvate kinase, cytosolic isozyme~ PKM2~ complete |
| pyruvate kinase | Ma06_p14950.1 | chr06 | 10182281 | 10190254 | Ma06_g14950~ Putative Pyruvate kinase, cytosolic isozyme~ pkiA~ complete |
| pyruvate kinase | Ma06_p17840.1 | chr06 | 12118394 | 12119005 | Ma06_g17840~ Pyruvate kinase, cytosolic isozyme~ Pkm2~ remnant |
| pyruvate kinase | Ma06_p38800.1 | chr06 | 37438357 | 37438964 | Ma06_g38800~ Pyruvate kinase, cytosolic isozyme~ pyk~ remnant |
| pyruvate kinase | Ma07_p17230.1 | chr07 | 16667646 | 16710150 | Ma07_g17230~ Pyruvate kinase isozyme A, chloroplastic~ pykF~ pseudogene |
| pyruvate kinase | Ma07_p23210.1 | chr07 | 30943911 | 30945416 | Ma07_g23210~ Pyruvate kinase, cytosolic isozyme~ pkiA~ complete |
| pyruvate kinase | Ma07_p26120.1 | chr07 | 33046978 | 33053835 | Ma07_g26120~ Pyruvate kinase isozyme G, chloroplastic~ pyk~ complete |
| pyruvate kinase | Ma08_p13380.1 | chr08 | 10554685 | 10559541 | Ma08_g13380~ Pyruvate kinase, cytosolic isozyme~ PKM2~ complete |
| pyruvate kinase | Ma08_p15530.1 | chr08 | 15729139 | 15735100 | Ma08_g15530~ Pyruvate kinase isozyme G, chloroplastic~ pyk~ complete |
| pyruvate kinase | Ma09_p08070.1 | chr09 | 5303388 | 5315598 | Ma09_g08070~ probable pyruvate kinase, cytosolic isozyme~ unknown_gene~ missing_functional_completeness |
| pyruvate kinase | Ma09_p24220.1 | chr09 | 35902688 | 35906649 | Ma09_g24220~ Pyruvate kinase, cytosolic isozyme~ PKM2~ complete |
| pyruvate kinase | Ma10_p04950.1 | chr10 | 15589645 | 15592987 | Ma10_g04950~ Pyruvate kinase, cytosolic isozyme~ PKM2~ complete |
| pyruvate kinase | Ma10_p15480.1 | chr10 | 27553717 | 27562525 | Ma10_g15480~ Putative Pyruvate kinase, cytosolic isozyme~ pkiA~ complete |
| pyruvate kinase | Ma11_p09970.1 | chr11 | 9163687 | 9171501 | Ma11_g09970~ Putative Pyruvate kinase, cytosolic isozyme~ pkiA~ complete |
