# Supplementary material for: Expression Fluctuations of Genes Involved in Carbohydrate Metabolism Affected by Alterations of Ethylene Biosynthesis Associated with Ripening in Banana Fruit
Source: Plants (Basel). 2020 Aug 30;9(9):1120. doi: 10.3390/plants9091120 (PMC7570234; doi:10.3390/plants9091120)
Supplement: Supplementary file 1 [file plants-09-01120-s001.zip › Table_S4.docx]

Table S4. The fragments per kilobase of transcript per million mapped reads (FPKM) values of selected relevant genes in starch and sucrose metabolism which were applied in this study.

| Gene Name Used in This Research | Contig ID | FPKM_WT | | FPKM_As1 | FPKM_As2 |
| --- | --- | --- | --- | --- | --- |
| 4-alpha-glucanotransferase | c49299_g1 | | 175.67 | 35.91 | 30.58 |
| 4-alpha-glucanotransferase | c50425_g1 | | 172.97 | 113.93 | 44.62 |
| alpha,alpha-trehalose-phosphate synthase | c52098_g1 | | 9.54 | 7.49 | 10.6 |
| alpha,alpha-trehalose-phosphate synthase | c53331_g1 | | 6.63 | 7.16 | 10.58 |
| alpha,alpha-trehalose-phosphate synthase | c46632_g1 | | 8.8 | 13.2 | 10.18 |
| alpha,alpha-trehalose-phosphate synthase | c49199_g1 | | 13.77 | 30.59 | 9.1 |
| alpha,alpha-trehalose-phosphate synthase | c52208_g2 | | 24.14 | 4.19 | 8.47 |
| alpha,alpha-trehalose-phosphate synthase | c54598_g2 | | 50.81 | 15.31 | 6.62 |
| alpha,alpha-trehalose-phosphate synthase | c52098_g3 | | 10.74 | 10.02 | 4.5 |
| alpha,alpha-trehalose-phosphate synthase | c52098_g4 | | 14.12 | 2.97 | 3.74 |
| alpha,alpha-trehalose-phosphate synthase | c51447_g1 | | 22.31 | 12.17 | 3.71 |
| alpha,alpha-trehalose-phosphate synthase | c47169_g1 | | 0.8 | 8.36 | 3.07 |
| alpha,alpha-trehalose-phosphate synthase | c47682_g1 | | 1.24 | 6.36 | 3.05 |
| alpha,alpha-trehalose-phosphate synthase | c54598_g1 | | 10.39 | 1.65 | 1.77 |
| alpha,alpha-trehalose-phosphate synthase | c52297_g1 | | 26.23 | 2.75 | 1.29 |
| alpha,alpha-trehalose-phosphate synthase | c32908_g1 | | 1.68 | 1.03 | 0.99 |
| alpha,alpha-trehalose-phosphate synthase | c57236_g1 | | 5.69 | 1.89 | 0.73 |
| alpha,alpha-trehalose-phosphate synthase | c31768_g1 | | 5.41 | 1.2 | 0.5 |
| alpha,alpha-trehalose-phosphate synthase | c52208_g1 | | 13.6 | 5.23 | 0.06 |
| alpha,alpha-trehalose-phosphate synthase | c5968_g1 | | 17.65 | 0.06 | 0.06 |
| alpha-amylase | c52310_g1 | | 249.35 | 1516.61 | 1400.98 |
| alpha-amylase | c54349_g3 | | 126.61 | 28.45 | 75.36 |
| alpha-amylase | c54349_g1 | | 99.56 | 21.13 | 47.64 |
| alpha-amylase | c45374_g1 | | 77.21 | 53.75 | 69.11 |
| alpha-amylase | c51480_g5 | | 3.43 | 2.95 | 2.86 |
| alpha-amylase | c86312_g1 | | 1.58 | 5.63 | 1.68 |
| alpha-amylase | c51780_g1 | | 0.7 | 2.57 | 0.41 |
| alpha-glucosidase | c54466_g1 | | 23.52 | 42.95 | 21.26 |
| alpha-glucosidase | c46404_g2 | | 18 | 1.23 | 0.52 |
| alpha-glucosidase | c54677_g1 | | 191.97 | 463.42 | 186.65 |
| alpha-glucosidase | c46404_g3 | | 30.43 | 3.29 | 1.9 |
| alpha-glucosidase | c52318_g2 | | 17.7 | 20.14 | 16.89 |
| alpha-glucosidase | c46404_g1 | | 9.95 | 1.59 | 1 |
| beta-amylase | c76567_g1 | | 799.73 | 4271.54 | 2395.13 |
| beta-amylase | c51322_g1 | | 61.91 | 220.76 | 267.68 |
| beta-amylase | c21249_g1 | | 4.23 | 23.62 | 63.66 |
| beta-amylase | c67265_g1 | | 3.37 | 20.24 | 32.08 |
| beta-amylase | c52556_g1 | | 36.49 | 18.5 | 24.1 |
| beta-amylase | c52780_g1 | | 5.76 | 22.68 | 23.51 |
| beta-amylase | c43403_g2 | | 37.7 | 0.72 | 13.42 |

**Table S4.** *Cont*.

| Gene Name Used in This Research | Contig ID | FPKM_WT | FPKM_As1 | FPKM_As2 |
| --- | --- | --- | --- | --- |
| beta-amylase | c52556_g2 | 11.91 | 6.3 | 7.54 |
| beta-amylase | c52492_g1 | 5.69 | 7.19 | 5.46 |
| beta-amylase | c52492_g2 | 7.2 | 7.17 | 5.1 |
| beta-amylase | c43403_g1 | 28.67 | 1.18 | 4.53 |
| beta-amylase | c25433_g1 | 3.04 | 14.47 | 3.25 |
| beta-amylase | c63781_g1 | 0.54 | 0.06 | 0.62 |
| beta-amylase | c6894_g1 | 5.2 | 0.06 | 0.06 |
| beta-amylase | c63373_g1 | 4.03 | 0.06 | 0.06 |
| beta-fructofuranosidase | c42253_g1 | 107.49 | 694.84 | 1743.23 |
| beta-fructofuranosidase | c52225_g1 | 14.61 | 132.01 | 82.78 |
| beta-fructofuranosidase | c15726_g2 | 2.06 | 0.06 | 2.16 |
| beta-fructofuranosidase | c57762_g1 | 1.77 | 0.06 | 1.96 |
| beta-fructofuranosidase | c69201_g1 | 5.96 | 0.06 | 1.62 |
| beta-fructofuranosidase | c35477_g1 | 3.28 | 2.66 | 1.24 |
| beta-fructofuranosidase | c11214_g1 | 8.22 | 0.06 | 1.13 |
| beta-fructofuranosidase | c62927_g1 | 3.6 | 0.06 | 0.81 |
| beta-fructofuranosidase | c24253_g1 | 2.17 | 0.06 | 0.51 |
| beta-fructofuranosidase | c21718_g1 | 2.82 | 0.44 | 0.06 |
| beta-fructofuranosidase | c31650_g1 | 6.29 | 7.34 | 0.06 |
| beta-fructofuranosidase | c15726_g1 | 3.27 | 0.73 | 0.06 |
| beta-fructofuranosidase | c86512_g1 | 0.93 | 0.06 | 0.06 |
| beta-glucosidase | c50675_g1 | 1070.45 | 2599.75 | 2782.79 |
| beta-glucosidase | c52267_g1 | 757.52 | 210.37 | 573.91 |
| beta-glucosidase | c46503_g1 | 100.93 | 14.13 | 66.12 |
| beta-glucosidase | c52110_g2 | 75.12 | 77.52 | 54.21 |
| beta-glucosidase | c48655_g1 | 70.83 | 35.26 | 48.74 |
| beta-glucosidase | c46395_g1 | 44.08 | 5.99 | 5.58 |
| beta-glucosidase | c48941_g2 | 42.18 | 74.39 | 44.59 |
| beta-glucosidase | c35688_g1 | 37.48 | 3.83 | 15.49 |
| beta-glucosidase | c48972_g1 | 36.07 | 0.48 | 1.06 |
| beta-glucosidase | c41359_g1 | 33.09 | 9.85 | 5.75 |
| beta-glucosidase | c39241_g1 | 23.61 | 71.39 | 177.44 |
| beta-glucosidase | c49720_g1 | 22.8 | 30 | 29.67 |
| beta-glucosidase | c51217_g1 | 20.73 | 0.57 | 0.37 |
| beta-glucosidase | c46886_g1 | 20.16 | 0.34 | 0.8 |
| beta-glucosidase | c45791_g1 | 20.13 | 32.03 | 49.93 |
| beta-glucosidase | c50070_g1 | 16.29 | 1.29 | 1.2 |
| beta-glucosidase | c50658_g1 | 15.94 | 1.16 | 0.32 |
| beta-glucosidase | c27085_g1 | 15.35 | 6.94 | 1.06 |
| beta-glucosidase | c51221_g1 | 15.25 | 9.03 | 9.96 |
| beta-glucosidase | c43689_g1 | 13.02 | 2.48 | 9.71 |
| beta-glucosidase | c38517_g1 | 12.51 | 0.06 | 1.28 |

**Table S4**. *Cont*.

| Gene Name Used in This Research | Contig ID | FPKM_WT | FPKM_As1 | FPKM_As2 |
| --- | --- | --- | --- | --- |
| beta-glucosidase | c53232_g1 | 12.5 | 3.11 | 9.16 |
| beta-glucosidase | c35185_g3 | 10.84 | 10.28 | 0.41 |
| beta-glucosidase | c71626_g1 | 10.44 | 0.06 | 0.06 |
| beta-glucosidase | c49823_g1 | 10.09 | 2.94 | 1.09 |
| beta-glucosidase | c47731_g1 | 9.74 | 1.05 | 1.42 |
| beta-glucosidase | c29581_g1 | 9.7 | 0.06 | 1.25 |
| beta-glucosidase | c83061_g1 | 9.7 | 0.06 | 0.06 |
| beta-glucosidase | c24485_g1 | 8.49 | 0.06 | 0.06 |
| beta-glucosidase | c49666_g2 | 8.22 | 8.46 | 0.93 |
| beta-glucosidase | c49666_g1 | 8.09 | 11.08 | 1.49 |
| beta-glucosidase | c46559_g1 | 8.01 | 1.3 | 2.52 |
| beta-glucosidase | c47731_g2 | 7.81 | 1.88 | 1.71 |
| beta-glucosidase | c54487_g2 | 7.78 | 1.73 | 1.68 |
| beta-glucosidase | c49404_g1 | 7.55 | 3 | 3.86 |
| beta-glucosidase | c29855_g1 | 7.21 | 10.47 | 5.19 |
| beta-glucosidase | c50064_g1 | 7.14 | 0.48 | 3.73 |
| beta-glucosidase | c50648_g1 | 6.97 | 1.13 | 1.13 |
| beta-glucosidase | c51221_g2 | 6.63 | 3.8 | 6.6 |
| beta-glucosidase | c70603_g1 | 6.12 | 0.06 | 0.06 |
| beta-glucosidase | c17825_g1 | 5.93 | 0.06 | 0.49 |
| beta-glucosidase | c37056_g1 | 5.45 | 3.25 | 0.2 |
| beta-glucosidase | c38766_g1 | 5.41 | 8.6 | 1.94 |
| beta-glucosidase | c835_g1 | 5.2 | 0.06 | 0.06 |
| beta-glucosidase | c53681_g1 | 4.85 | 3.16 | 4.48 |
| beta-glucosidase | c43545_g1 | 4.49 | 3.24 | 3.03 |
| beta-glucosidase | c47516_g1 | 4.21 | 28.43 | 19.39 |
| beta-glucosidase | c30040_g1 | 4.04 | 0.96 | 0.31 |
| beta-glucosidase | c24485_g2 | 4.03 | 0.7 | 0.06 |
| beta-glucosidase | c42653_g1 | 4.02 | 1.08 | 0.42 |
| beta-glucosidase | c56848_g1 | 3.86 | 0.06 | 0.06 |
| beta-glucosidase | c50070_g1 | 16.29 | 1.29 | 1.2 |
| beta-glucosidase | c33712_g1 | 3.6 | 0.06 | 0.85 |
| beta-glucosidase | c23259_g1 | 3.53 | 0.06 | 0.06 |
| beta-glucosidase | c43919_g2 | 3.43 | 7.95 | 3.27 |
| beta-glucosidase | c27988_g1 | 2.82 | 1.1 | 2.28 |
| beta-glucosidase | c59807_g1 | 2.82 | 0.06 | 0.06 |
| beta-glucosidase | c38983_g1 | 2.79 | 0.27 | 0.31 |
| beta-glucosidase | c37905_g1 | 2.76 | 0.06 | 0.52 |
| beta-glucosidase | c43919_g1 | 2.74 | 5.98 | 1.68 |
| beta-glucosidase | c38983_g2 | 2.68 | 3.05 | 0.22 |
| beta-glucosidase | c28672_g1 | 2.49 | 0.16 | 0.31 |
| beta-glucosidase | c34935_g3 | 2.47 | 3.41 | 0.72 |

**Table S4**. *Cont*.

| Gene Name Used in This Research | Contig ID | FPKM_WT | FPKM_As1 | FPKM_As2 |
| --- | --- | --- | --- | --- |
| beta-glucosidase | c14709_g1 | 2.39 | 0.17 | 0.06 |
| beta-glucosidase | c85893_g1 | 2.2 | 2.6 | 0.06 |
| beta-glucosidase | c2153_g1 | 2.04 | 0.06 | 0.06 |
| beta-glucosidase | c25880_g1 | 1.99 | 2.26 | 0.06 |
| beta-glucosidase | c28287_g1 | 1.81 | 0.06 | 0.06 |
| beta-glucosidase | c84623_g1 | 1.71 | 0.69 | 1.29 |
| beta-glucosidase | c47677_g1 | 1.65 | 4.93 | 0.89 |
| beta-glucosidase | c1020_g1 | 1.63 | 0.5 | 0.48 |
| beta-glucosidase | c63680_g1 | 1.57 | 0.06 | 0.06 |
| beta-glucosidase | c65911_g1 | 1.57 | 0.06 | 0.06 |
| beta-glucosidase | c21962_g1 | 1.56 | 0.9 | 0.99 |
| beta-glucosidase | c15134_g1 | 1.49 | 0.52 | 0.25 |
| beta-glucosidase | c43461_g1 | 1.36 | 0.66 | 4.23 |
| beta-glucosidase | c26075_g1 | 1.26 | 0.06 | 0.66 |
| beta-glucosidase | c26075_g2 | 1.25 | 0.06 | 1.46 |
| beta-glucosidase | c57951_g1 | 1.04 | 1.27 | 0.06 |
| beta-glucosidase | c47677_g2 | 1.01 | 8.28 | 0.51 |
| beta-glucosidase | c72123_g1 | 0.99 | 3.05 | 0.29 |
| beta-glucosidase | c1440_g1 | 0.93 | 1.14 | 0.54 |
| beta-glucosidase | c3880_g1 | 0.86 | 1.02 | 0.33 |
| beta-glucosidase | c82383_g1 | 0.68 | 2.23 | 0.06 |
| beta-glucosidase | c28287_g2 | 0.46 | 1.14 | 0.37 |
| beta-glucosidase | c23436_g1 | 0.42 | 1.3 | 1.25 |
| beta-glucosidase | c67299_g1 | 0.41 | 0.5 | 2.35 |
| beta-glucosidase | c82026_g1 | 0.34 | 1.03 | 0.6 |
| beta-glucosidase | c34935_g2 | 0.22 | 1.08 | 0.06 |
| beta-glucosidase | c35566_g1 | 0.16 | 2.38 | 2.86 |
| beta-glucosidase | c84146_g1 | 0.06 | 2.73 | 5.04 |
| beta-glucosidase | c85237_g1 | 0.06 | 0.06 | 4.67 |
| beta-glucosidase | c78028_g1 | 0.06 | 0.06 | 4.48 |
| beta-glucosidase | c6221_g1 | 0.06 | 0.06 | 3.05 |
| beta-glucosidase | c57737_g1 | 0.06 | 4.97 | 2.98 |
| beta-glucosidase | c64494_g1 | 0.06 | 1.95 | 1.74 |
| beta-glucosidase | c71695_g1 | 0.06 | 3.82 | 1.71 |
| beta-glucosidase | c8882_g1 | 0.06 | 0.12 | 1.44 |
| beta-glucosidase | c66783_g1 | 0.06 | 1.58 | 1.42 |
| beta-glucosidase | c27799_g1 | 0.06 | 1.9 | 1.38 |
| beta-glucosidase | c23354_g1 | 0.06 | 0.97 | 1.38 |
| beta-glucosidase | c83163_g1 | 0.06 | 4.57 | 0.06 |
| beta-glucosidase | c34935_g1 | 0.06 | 3.85 | 0.06 |
| beta-glucosidase | c60837_g1 | 0.06 | 3.57 | 0.06 |
| beta-glucosidase | c531_g1 | 0.06 | 3.37 | 0.06 |

**Table S4**. *Cont*.

| Gene Name Used in This Research | Contig ID | FPKM_WT | FPKM_As1 | FPKM_As2 |
| --- | --- | --- | --- | --- |
| fructokinase | c45538_g1 | 96.4 | 110.92 | 155.56 |
| fructokinase | c49411_g1 | 44.83 | 58.37 | 96.71 |
| fructokinase | c40989_g1 | 15.29 | 52.15 | 70.83 |
| fructokinase | c51816_g1 | 32.23 | 67.37 | 66.38 |
| fructokinase | c4232_g1 | 26.7 | 45.1 | 36.86 |
| fructokinase | c51816_g2 | 3.35 | 21.04 | 35.64 |
| fructokinase | c39992_g1 | 13.11 | 21.85 | 29.46 |
| fructokinase | c53934_g1 | 41.84 | 32.68 | 22.09 |
| fructokinase | c32478_g1 | 15.7 | 27.74 | 21.47 |
| fructokinase | c47498_g1 | 38.39 | 9.19 | 18.29 |
| fructokinase | c61301_g1 | 3.71 | 16.6 | 17.71 |
| fructokinase | c15285_g1 | 7.22 | 15.48 | 14.26 |
| fructokinase | c49411_g2 | 2.39 | 8.92 | 12.55 |
| fructokinase | c29325_g1 | 9.93 | 16.33 | 12.27 |
| fructokinase | c34761_g1 | 6.51 | 10.44 | 10.88 |
| fructokinase | c25665_g1 | 7.07 | 1.56 | 5.07 |
| fructokinase | c48361_g1 | 6.78 | 3.66 | 4.5 |
| fructokinase | c39398_g1 | 1.93 | 3.63 | 3.86 |
| fructokinase | c35775_g1 | 3.62 | 3.51 | 3.15 |
| fructokinase | c37516_g1 | 0.21 | 3.11 | 2.53 |
| fructokinase | c72129_g1 | 1.2 | 0.06 | 1.41 |
| fructokinase | c57367_g1 | 0.55 | 0.06 | 1.24 |
| fructokinase | c17015_g1 | 0.06 | 0.72 | 1.22 |
| fructokinase | c33114_g1 | 2.3 | 1.21 | 0.38 |
| fructokinase | c32192_g1 | 3.09 | 0.45 | 0.35 |
| fructokinase | c1107_g1 | 4.96 | 0.61 | 0.06 |
| fructokinase | c10531_g1 | 3.2 | 0.44 | 0.06 |
| fructokinase | c14651_g1 | 8.36 | 0.06 | 0.06 |
| fructokinase | c80482_g1 | 5 | 0.06 | 0.06 |
| fructokinase | c86779_g1 | 1.34 | 0.06 | 0.06 |
| glucan phosphorylase | c48718_g1 | 568.54 | 103.47 | 158.35 |
| glucan phosphorylase | c51772_g1 | 2080.07 | 220.51 | 319.86 |
| glucose-1-phosphate adenylyltransferase | c51337_g1 | 450.29 | 1.24 | 8.47 |
| glucose-1-phosphate adenylyltransferase | c52175_g1 | 215.67 | 407.18 | 243.41 |
| glucose-1-phosphate adenylyltransferase | c51337_g2 | 159.76 | 2.6 | 11.21 |
| glucose-1-phosphate adenylyltransferase | c32971_g1 | 10.64 | 6.08 | 2.91 |
| glucose-1-phosphate adenylyltransferase | c31020_g1 | 8.24 | 5.63 | 0.91 |
| glucose-1-phosphate adenylyltransferase | c15709_g1 | 6.7 | 4.81 | 1.44 |
| glucose-1-phosphate adenylyltransferase | c8155_g1 | 4.49 | 2.29 | 0.94 |
| glucose-1-phosphate adenylyltransferase | c32971_g2 | 2.26 | 2.66 | 0.06 |
| glucose-1-phosphate adenylyltransferase | c42075_g1 | 1.25 | 8.84 | 1.27 |
| glucose-1-phosphate adenylyltransferase | c33461_g1 | 0.74 | 0.65 | 1.47 |

**Table S4**. *Cont*.

| Gene Name Used in This Research | Contig ID | FPKM_WT | FPKM_As1 | FPKM_As2 |
| --- | --- | --- | --- | --- |
| glucose-6-phosphate isomerase | c50453_g1 | 157.19 | 200.55 | 178.24 |
| glucose-6-phosphate isomerase | c51760_g1 | 67.95 | 127.39 | 134.97 |
| glucose-6-phosphate isomerase | c28438_g1 | 16.45 | 33.29 | 45.39 |
| glucose-6-phosphate isomerase | c24892_g1 | 13.77 | 8.02 | 14.44 |
| glucose-6-phosphate isomerase | c34689_g1 | 8.57 | 19.51 | 15.55 |
| glucose-6-phosphate isomerase | c28438_g2 | 6.93 | 54.39 | 31.25 |
| glucose-6-phosphate isomerase | c57520_g1 | 1.18 | 0.06 | 0.06 |
| pectinesterase | c38762_g1 | 190.08 | 1368.82 | 2034.12 |
| pectinesterase | c48232_g1 | 22.43 | 794.12 | 459.42 |
| pectinesterase | c23921_g1 | 0.06 | 55.22 | 54.13 |
| pectinesterase | c50418_g1 | 14.53 | 83.37 | 43.71 |
| pectinesterase | c29374_g1 | 17.48 | 45.36 | 29.54 |
| pectinesterase | c33986_g1 | 0.09 | 44.58 | 27.92 |
| pectinesterase | c44913_g2 | 13.74 | 28.3 | 25.09 |
| pectinesterase | c24921_g1 | 0.06 | 24.75 | 20.08 |
| pectinesterase | c38563_g1 | 6.63 | 12.12 | 13.05 |
| pectinesterase | c52386_g1 | 79.87 | 14.32 | 11.34 |
| pectinesterase | c45067_g1 | 1.69 | 19.23 | 9.34 |
| pectinesterase | c52618_g1 | 54.96 | 2.61 | 9.09 |
| pectinesterase | c44913_g1 | 4.85 | 0.06 | 5.03 |
| pectinesterase | c55413_g1 | 0.06 | 0.06 | 4.97 |
| pectinesterase | c25772_g1 | 0.06 | 1.91 | 4.87 |
| pectinesterase | c33012_g1 | 11.23 | 5.18 | 3.72 |
| pectinesterase | c53102_g1 | 7.04 | 16.4 | 3.58 |
| pectinesterase | c6179_g1 | 0.06 | 0.06 | 2.32 |
| pectinesterase | c66891_g1 | 0.06 | 0.06 | 2.17 |
| pectinesterase | c24583_g1 | 2.03 | 2.5 | 1.8 |
| pectinesterase | c24033_g1 | 0.09 | 2.2 | 1.61 |
| pectinesterase | c80953_g1 | 0.56 | 0.68 | 1.26 |
| pectinesterase | c71826_g1 | 0.9 | 1.08 | 0.99 |
| pectinesterase | c68393_g1 | 0.06 | 0.97 | 0.9 |
| pectinesterase | c54882_g2 | 11.44 | 2.26 | 0.84 |
| pectinesterase | c5930_g1 | 0.06 | 0.68 | 0.63 |
| pectinesterase | c35700_g1 | 6.25 | 0.21 | 0.61 |
| pectinesterase | c5930_g2 | 0.49 | 0.9 | 0.57 |
| pectinesterase | c31355_g1 | 10.42 | 1.01 | 0.49 |
| pectinesterase | c70511_g1 | 5.64 | 0.06 | 0.46 |
| pectinesterase | c4671_g1 | 3.45 | 0.57 | 0.14 |
| pectinesterase | c75071_g1 | 0.06 | 4.61 | 0.06 |
| pectinesterase | c16353_g1 | 0.5 | 4.3 | 0.06 |
| pectinesterase | c73243_g1 | 0.06 | 3.61 | 0.06 |
| pectinesterase | c68263_g1 | 0.06 | 3.57 | 0.06 |

**Table S4**. *Cont*.

| Gene Name Used in This Research | Contig ID | FPKM_WT | FPKM_As1 | FPKM_As2 |
| --- | --- | --- | --- | --- |
| pectinesterase | c16353_g2 | 0.06 | 1.05 | 0.06 |
| pectinesterase | c73526_g1 | 1.25 | 1.02 | 0.06 |
| pectinesterase | c57595_g1 | 0.06 | 0.96 | 0.06 |
| pectinesterase | c12418_g1 | 5.36 | 0.06 | 0.06 |
| pectinesterase | c68453_g1 | 4.35 | 0.06 | 0.06 |
| pectinesterase | c78794_g1 | 0.06 | 0.06 | 0.06 |
| phosphoglucomutase | c53381_g1 | 158.83 | 28.34 | 37.93 |
| phosphoglucomutase | c52097_g1 | 106.56 | 164.44 | 141.24 |
| phosphoglucomutase | c7447_g1 | 19 | 85 | 44.34 |
| polygalacturonase | c54028_g1 | 127.04 | 110.2 | 99.05 |
| polygalacturonase | c51994_g1 | 23.37 | 26.04 | 22.26 |
| polygalacturonase | c54619_g1 | 21.26 | 39.49 | 35.17 |
| polygalacturonase | c52074_g2 | 20.28 | 29.23 | 18.73 |
| polygalacturonase | c51231_g1 | 18 | 22.74 | 24.26 |
| polygalacturonase | c34926_g1 | 13.77 | 9.03 | 50.17 |
| polygalacturonase | c51231_g2 | 13.72 | 23.31 | 27.07 |
| polygalacturonase | c44422_g1 | 13.55 | 86.18 | 10.78 |
| polygalacturonase | c48022_g1 | 10.42 | 23.58 | 30.04 |
| polygalacturonase | c37220_g1 | 10.31 | 6.83 | 8.41 |
| polygalacturonase | c47894_g1 | 10.19 | 4.37 | 2.34 |
| polygalacturonase | c41193_g1 | 8.7 | 2.51 | 2.27 |
| polygalacturonase | c39780_g1 | 7.49 | 1.28 | 1.24 |
| polygalacturonase | c51231_g3 | 7.36 | 6.12 | 10.63 |
| polygalacturonase | c47209_g1 | 7.25 | 0.18 | 0.99 |
| polygalacturonase | c57619_g1 | 7.11 | 0.06 | 0.06 |
| polygalacturonase | c37277_g1 | 6.86 | 55.18 | 3.07 |
| polygalacturonase | c73627_g1 | 6.12 | 0.06 | 0.06 |
| polygalacturonase | c51994_g3 | 5.99 | 4.55 | 4.34 |
| polygalacturonase | c51231_g4 | 5.89 | 15.83 | 11.11 |
| polygalacturonase | c54539_g1 | 5.31 | 1.49 | 2.66 |
| polygalacturonase | c65750_g1 | 4.51 | 0.06 | 0.06 |
| polygalacturonase | c37220_g2 | 3.15 | 1.88 | 3.36 |
| polygalacturonase | c36983_g2 | 2.94 | 14.08 | 6.89 |
| polygalacturonase | c41887_g1 | 2.91 | 20.16 | 29.98 |
| polygalacturonase | c46789_g2 | 2.74 | 10.74 | 1.94 |
| polygalacturonase | c40025_g1 | 2.69 | 0.82 | 1.77 |
| polygalacturonase | c46789_g3 | 2.67 | 1.08 | 2.06 |
| polygalacturonase | c9248_g1 | 2.55 | 0.06 | 0.06 |
| polygalacturonase | c24780_g1 | 2.29 | 0.56 | 1.05 |
| polygalacturonase | c73004_g1 | 2.2 | 0.06 | 0.06 |
| polygalacturonase | c45615_g1 | 1.91 | 19.57 | 0.99 |
| polygalacturonase | c77870_g1 | 1.56 | 0.38 | 0.06 |

**Table S4**. *Cont*.

| Gene Name Used in This Research | Contig ID | FPKM_WT | FPKM_As1 | FPKM_As2 |
| --- | --- | --- | --- | --- |
| polygalacturonase | c36983_g1 | 1.52 | 11.85 | 1.67 |
| polygalacturonase | c28034_g1 | 1.4 | 7.4 | 0.6 |
| polygalacturonase | c27931_g1 | 1.35 | 3.73 | 0.39 |
| polygalacturonase | c59554_g1 | 1.3 | 0.06 | 0.06 |
| polygalacturonase | c31263_g1 | 1.25 | 0.06 | 0.06 |
| polygalacturonase | c31740_g1 | 0.71 | 7.29 | 0.61 |
| polygalacturonase | c46789_g1 | 0.67 | 2.09 | 1.67 |
| polygalacturonase | c52074_g1 | 0.65 | 1.03 | 3.61 |
| polygalacturonase | c8162_g1 | 0.61 | 1.87 | 6.4 |
| polygalacturonase | c86898_g1 | 0.56 | 1.37 | 0.64 |
| polygalacturonase | c16754_g1 | 0.52 | 5.11 | 0.6 |
| polygalacturonase | c27472_g1 | 0.34 | 1.25 | 3.56 |
| polygalacturonase | c79297_g1 | 0.25 | 1.84 | 0.29 |
| polygalacturonase | c83390_g1 | 0.24 | 2.33 | 1.67 |
| polygalacturonase | c72978_g1 | 0.22 | 2.13 | 0.06 |
| polygalacturonase | c37944_g1 | 0.16 | 7.79 | 0.95 |
| polygalacturonase | c40696_g1 | 0.06 | 20.84 | 113.9 |
| polygalacturonase | c38853_g1 | 0.06 | 2.2 | 23.4 |
| polygalacturonase | c82463_g1 | 0.06 | 0.06 | 3.48 |
| polygalacturonase | c38853_g2 | 0.06 | 8.98 | 2 |
| polygalacturonase | c2233_g1 | 0.06 | 0.5 | 1.88 |
| polygalacturonase | c71739_g1 | 0.06 | 0.06 | 1.51 |
| polygalacturonase | c34506_g1 | 0.06 | 3.99 | 0.91 |
| polygalacturonase | c19915_g1 | 0.06 | 4.22 | 0.06 |
| polygalacturonase | c54404_g7 | 0.06 | 3.51 | 0.06 |
| polygalacturonase | c6680_g1 | 0.06 | 3.39 | 0.06 |
| polygalacturonase | c64499_g1 | 0.06 | 1.95 | 0.06 |
| polygalacturonase | c54539_g2 | 0.06 | 1.91 | 0.06 |
| polygalacturonate 4-alpha-galacturonosyltransferase | c52171_g1 | 57.8 | 58.49 | 32.06 |
| polygalacturonate 4-alpha-galacturonosyltransferase | c12420_g1 | 16.64 | 27.27 | 17.79 |
| polygalacturonate 4-alpha-galacturonosyltransferase | c45130_g2 | 7.95 | 14.25 | 6.06 |
| polygalacturonate 4-alpha-galacturonosyltransferase | c45130_g1 | 6.71 | 8.59 | 6.82 |
| polygalacturonate 4-alpha-galacturonosyltransferase | c17520_g1 | 5.62 | 13.22 | 6.47 |
| starch synthase | c45910_g1 | 4213.88 | 186.12 | 272.05 |
| starch synthase | c49089_g1 | 6.33 | 115.42 | 73.25 |
| starch synthase | c52693_g1 | 30.99 | 46.33 | 32.63 |
| starch synthase | c46989_g1 | 2.05 | 6.09 | 10 |

**Table S4**. *Cont*.

| Gene Name Used in This Research | Contig ID | FPKM_WT | FPKM_As1 | FPKM_As2 |
| --- | --- | --- | --- | --- |
| starch synthase | c54174_g1 | 13.32 | 8.6 | 8.85 |
| starch synthase | c43237_g1 | 10.38 | 8.02 | 8.79 |
| starch synthase | c44565_g2 | 102.96 | 11.62 | 8.22 |
| starch synthase | c52189_g1 | 3.5 | 6.01 | 6.8 |
| starch synthase | c44565_g1 | 130.22 | 14.24 | 6.65 |
| starch synthase | c16672_g1 | 12.6 | 3.07 | 3.45 |
| starch synthase | c65361_g1 | 6.47 | 0.06 | 3.3 |
| starch synthase | c54640_g1 | 35.37 | 6.3 | 2.77 |
| starch synthase | c73146_g1 | 2.42 | 0.06 | 2.51 |
| starch synthase | c12989_g1 | 8.11 | 0.06 | 2.07 |
| starch synthase | c26376_g2 | 2.08 | 6.34 | 1.19 |
| starch synthase | c35316_g1 | 5.6 | 0.81 | 1.15 |
| starch synthase | c73348_g1 | 3.38 | 0.19 | 0.55 |
| starch synthase | c35035_g1 | 3.76 | 1.34 | 0.47 |
| starch synthase | c8284_g1 | 5.1 | 0.06 | 0.45 |
| starch synthase | c26376_g1 | 1.81 | 4.13 | 0.3 |
| starch synthase | c27730_g1 | 0.56 | 3.68 | 0.22 |
| starch synthase | c81187_g1 | 0.06 | 5.15 | 0.06 |
| starch synthase | c537_g1 | 0.06 | 4.73 | 0.06 |
| starch synthase | c24820_g1 | 0.72 | 0.87 | 0.06 |
| starch synthase | c4352_g1 | 4.51 | 0.06 | 0.06 |
| starch synthase | c66436_g1 | 1.2 | 0.06 | 0.06 |
| starch synthase | c84203_g1 | 0.6 | 0.06 | 0.06 |
| starch synthase | c14230_g1 | 0.06 | 0.06 | 0.06 |
| sucrose synthase | c53867_g1 | 578.33 | 374.06 | 109.5 |
| sucrose synthase | c904_g1 | 234.4 | 193.02 | 148.44 |
| sucrose synthase | c21522_g1 | 175.55 | 111.71 | 64.04 |
| sucrose synthase | c41458_g1 | 96.77 | 90.22 | 69.13 |
| sucrose synthase | c52822_g1 | 47.9 | 4.72 | 11.07 |
| sucrose synthase | c25936_g1 | 9.06 | 11.96 | 4.58 |
| sucrose synthase | c46712_g2 | 8.64 | 12.71 | 3.62 |
| sucrose synthase | c46712_g3 | 8.35 | 11.7 | 2.33 |
| sucrose synthase | c46712_g4 | 6.67 | 8.15 | 5.62 |
| sucrose synthase | c46712_g1 | 5.25 | 10.31 | 2.3 |
| sucrose synthase | c26965_g1 | 0.06 | 5.58 | 4.82 |
| sucrose-phosphate synthase | c48469_g1 | 1674.54 | 430.06 | 778.8 |
| sucrose-phosphate synthase | c51306_g1 | 34.77 | 36.76 | 22.22 |
| sucrose-phosphate synthase | c52943_g1 | 17.89 | 1.81 | 1.63 |
| sucrose-phosphate synthase | c20599_g1 | 6.09 | 3.26 | 4.41 |
| sucrose-phosphate synthase | c36898_g1 | 5.93 | 2.01 | 4.35 |
| sucrose-phosphate synthase | c20684_g1 | 3.46 | 3.39 | 0.8 |
| sucrose-phosphate synthase | c78118_g1 | 1.91 | 0.67 | 1.6 |

**Table S4**. *Cont*.

| Gene Name Used in This Research | Contig ID | FPKM_WT | FPKM_As1 | FPKM_As2 |
| --- | --- | --- | --- | --- |
| UDP-glucose 6-dehydrogenase | c53042_g1 | 90.73 | 381.36 | 219.18 |
| UDP-glucose 6-dehydrogenase | c50623_g2 | 51.62 | 218.91 | 108.76 |
| UDP-glucose 6-dehydrogenase | c14596_g1 | 26.94 | 97.11 | 26.93 |
| UDP-glucose 6-dehydrogenase | c34840_g2 | 24.4 | 76.75 | 63.91 |
| UDP-glucose 6-dehydrogenase | c28757_g2 | 16.99 | 9.81 | 8.5 |
| UDP-glucose 6-dehydrogenase | c34840_g1 | 15.08 | 60.99 | 29.27 |
| UDP-glucose 6-dehydrogenase | c28952_g1 | 10.32 | 78.62 | 20.68 |
| UDP-glucose 6-dehydrogenase | c38099_g2 | 7.11 | 64.33 | 50.47 |
| UDP-glucose 6-dehydrogenase | c45970_g1 | 6.95 | 0.51 | 0.26 |
| UDP-glucose 6-dehydrogenase | c24946_g1 | 5.63 | 45.95 | 13.84 |
| UDP-glucose 6-dehydrogenase | c36563_g1 | 5.32 | 52.12 | 10.3 |
| UDP-glucose 6-dehydrogenase | c38099_g1 | 2.82 | 53.52 | 41 |
| UDP-glucose 6-dehydrogenase | c53042_g2 | 1.55 | 41.48 | 27.83 |
| UDP-glucose 6-dehydrogenase | c12455_g1 | 1.41 | 0.06 | 0.06 |
