# Supplementary material for: Expression Fluctuations of Genes Involved in Carbohydrate Metabolism Affected by Alterations of Ethylene Biosynthesis Associated with Ripening in Banana Fruit
Source: Plants (Basel). 2020 Aug 30;9(9):1120. doi: 10.3390/plants9091120 (PMC7570234; doi:10.3390/plants9091120)
Supplement: Supplementary file 1 [file plants-09-01120-s001.zip › Table_S5.docx]

Table S5. The fragments per kilobase of transcript per million mapped reads (FPKM) values of selected relevant genes in glycolysis and gluconeogenesis which were applied in this study.

| Gene Name Used in This Research | Contig ID | FPKM_WT | FPKM_As1 | FPKM_As2 |
| --- | --- | --- | --- | --- |
| 2,3-bisphosphoglycerate-independent phosphoglycerate mutase | c50953_g1 | 115.19 | 309.65 | 202.96 |
| 2,3-bisphosphoglycerate-independent phosphoglycerate mutase | c72101_g1 | 29.27 | 33.59 | 47.85 |
| 2,3-bisphosphoglycerate-independent phosphoglycerate mutase | c26131_g2 | 27.42 | 31.89 | 34.78 |
| 2,3-bisphosphoglycerate-independent phosphoglycerate mutase | c26131_g1 | 18.5 | 24.89 | 24.39 |
| 2,3-bisphosphoglycerate-independent phosphoglycerate mutase | c35212_g1 | 2.37 | 5.57 | 9.83 |
| 2,3-bisphosphoglycerate-independent phosphoglycerate mutase | c28332_g1 | 0.06 | 3.69 | 5.06 |
| 6-phosphofructokinase | c45538_g1 | 96.4 | 110.92 | 155.56 |
| 6-phosphofructokinase | c49411_g1 | 44.83 | 58.37 | 96.71 |
| 6-phosphofructokinase | c40989_g1 | 15.29 | 52.15 | 70.83 |
| 6-phosphofructokinase | c4232_g1 | 26.7 | 45.1 | 36.86 |
| 6-phosphofructokinase | c39992_g1 | 13.11 | 21.85 | 29.46 |
| 6-phosphofructokinase | c53934_g1 | 41.84 | 32.68 | 22.09 |
| 6-phosphofructokinase | c32478_g1 | 15.7 | 27.74 | 21.47 |
| 6-phosphofructokinase | c49411_g2 | 2.39 | 8.92 | 12.55 |
| 6-phosphofructokinase | c29325_g1 | 9.93 | 16.33 | 12.27 |
| 6-phosphofructokinase | c34761_g1 | 6.51 | 10.44 | 10.88 |
| 6-phosphofructokinase | c39398_g1 | 1.93 | 3.63 | 3.86 |
| 6-phosphofructokinase | c37516_g1 | 0.21 | 3.11 | 2.53 |
| 6-phosphofructokinase | c72129_g1 | 1.2 | 0.06 | 1.41 |
| 6-phosphofructokinase | c17015_g1 | 0.06 | 0.72 | 1.22 |
| 6-phosphofructokinase | c33114_g1 | 2.3 | 1.21 | 0.38 |
| 6-phosphofructokinase | c1107_g1 | 4.96 | 0.61 | 0.06 |
| 6-phosphofructokinase | c10531_g1 | 3.2 | 0.44 | 0.06 |
| 6-phosphofructokinase | c14651_g1 | 8.36 | 0.06 | 0.06 |
| 6-phosphofructokinase | c80482_g1 | 5 | 0.06 | 0.06 |
| 6-phosphofructokinase | c86779_g1 | 1.34 | 0.06 | 0.06 |
| alcohol dehydrogenase | c43785_g1 | 947.24 | 697.19 | 3283.99 |
| alcohol dehydrogenase | c33600_g1 | 2.38 | 96.74 | 286.36 |
| alcohol dehydrogenase | c8771_g1 | 508.08 | 75.29 | 67.59 |
| alcohol dehydrogenase | c45546_g1 | 366.01 | 82.92 | 58.49 |
| alcohol dehydrogenase | c48466_g1 | 121.2 | 28.66 | 44.79 |
| alcohol dehydrogenase | c48697_g1 | 37.33 | 60.87 | 33.36 |
| alcohol dehydrogenase | c40494_g1 | 223.21 | 38.13 | 23.94 |
| alcohol dehydrogenase | c14965_g1 | 108.02 | 17.3 | 19.69 |

**Table S5**. *Cont*.

| Gene Name Used in This Research | Contig ID | FPKM_WT | FPKM_As1 | FPKM_As2 | |
| --- | --- | --- | --- | --- | --- |
| alcohol dehydrogenase | c47659_g1 | 5.88 | 7.98 | 18.46 | |
| alcohol dehydrogenase | c47965_g1 | 21.58 | 8.13 | 14.15 | |
| alcohol dehydrogenase | c7799_g1 | 78.3 | 17.82 | 10.42 | |
| alcohol dehydrogenase | c28195_g1 | 4.15 | 21.57 | | 8.77 |
| alcohol dehydrogenase | c53375_g1 | 22.12 | 2.72 | | 2.92 |
| alcohol dehydrogenase | c47930_g1 | 12.38 | 1.26 | | 1.16 |
| alcohol dehydrogenase | c64038_g1 | 1.53 | 0.38 | | 0.36 |
| alcohol dehydrogenase | c15995_g1 | 3.54 | 0.06 | | 0.06 |
| alcohol dehydrogenase | c85102_g1 | 2.61 | 0.06 | | 0.06 |
| aldehyde dehydrogenase | c28491_g1 | 63.68 | 458.6 | | 441.24 |
| aldehyde dehydrogenase | c44335_g1 | 364.79 | 209.95 | | 295.48 |
| aldehyde dehydrogenase | c28709_g4 | 65.35 | 88.99 | | 269.72 |
| aldehyde dehydrogenase | c40029_g1 | 65.78 | 227.33 | | 257.8 |
| aldehyde dehydrogenase | c28709_g1 | 40.52 | 69.48 | | 180.12 |
| aldehyde dehydrogenase | c51088_g1 | 401.27 | 209.85 | | 150.32 |
| aldehyde dehydrogenase | c28709_g2 | 30.52 | 29.83 | | 111.91 |
| aldehyde dehydrogenase | c48272_g1 | 102.75 | 101.31 | | 105.87 |
| aldehyde dehydrogenase | c41550_g1 | 171.7 | 78.44 | | 80.17 |
| aldehyde dehydrogenase | c49921_g1 | 348.43 | 215.94 | | 61.5 |
| aldehyde dehydrogenase | c40835_g1 | 31.92 | 59.93 | | 55 |
| aldehyde dehydrogenase | c45411_g1 | 9.5 | 10.44 | | 23.52 |
| aldehyde dehydrogenase | c48290_g1 | 28.07 | 11.52 | | 22.89 |
| aldehyde dehydrogenase | c48290_g2 | 30.31 | 9.68 | | 16.6 |
| aldehyde dehydrogenase | c53891_g1 | 9.19 | 3.83 | | 10.89 |
| aldehyde dehydrogenase | c53255_g1 | 25.03 | 12.08 | | 8.38 |
| aldehyde dehydrogenase | c47187_g1 | 6.66 | 8.89 | | 6.39 |
| aldehyde dehydrogenase | c49586_g1 | 13.56 | 8.5 | | 5.58 |
| aldehyde dehydrogenase | c56872_g1 | 0.06 | 0.06 | | 5.03 |
| aldehyde dehydrogenase | c34990_g1 | 2.57 | 8.59 | | 3.62 |
| aldehyde dehydrogenase | c45550_g1 | 1.71 | 3.63 | | 1.57 |
| aldehyde dehydrogenase | c11894_g1 | 5.95 | 4.24 | | 1.13 |
| aldehyde dehydrogenase | c46076_g1 | 3.18 | 1.1 | | 1.03 |
| aldehyde dehydrogenase | c13415_g1 | 1.52 | 0.92 | | 0.85 |
| aldehyde dehydrogenase | c47187_g2 | 0.78 | 0.77 | | 0.56 |
| aldehyde dehydrogenase | c83213_g1 | 1.07 | 5.16 | | 0.06 |
| aldehyde dehydrogenase | c20404_g1 | 2.63 | 3.84 | | 0.06 |
| aldehyde dehydrogenase | c86273_g1 | 0.06 | 2.49 | | 0.06 |
| aldehyde dehydrogenase | c12959_g1 | 1.77 | 0.06 | | 0.06 |
| Aldolase | c51172_g1 | 298.03 | 869.52 | | 643.06 |
| Aldolase | c51754_g1 | 343.52 | 507.6 | | 595.63 |
| Aldolase | c46107_g1 | 56.03 | 83.51 | | 182.25 |
| Aldolase | c31039_g1 | 439.05 | 33.46 | | 143.37 |

**Table S5**. *Cont*.

| Gene Name Used in This Research | | Contig ID | | FPKM_WT | | FPKM_As1 | FPKM_As2 |
| --- | --- | --- | --- | --- | --- | --- | --- |
| Aldolase | c40759_g1 | | 78.43 | | 52.36 | | 81.03 |
| Aldolase | c52127_g2 | | 216.05 | | 44.05 | | 77.87 |
| Aldolase | c52127_g1 | | 142.18 | | 57.26 | | 69.24 |
| Aldolase | c35687_g1 | | 47.31 | | 46.17 | | 40.91 |
| Aldolase | c36318_g1 | | 67.82 | | 24.72 | | 29.38 |
| Aldolase | c44217_g2 | | 17.1 | | 25.94 | | 21.6 |
| Aldolase | c45508_g1 | | 17.32 | | 9.79 | | 18.17 |
| Aldolase | c41405_g1 | | 23.45 | | 53.19 | | 17.62 |
| Aldolase | c50975_g1 | | 26.92 | | 17.35 | | 15.9 |
| Aldolase | c47255_g1 | | 8.21 | | 13.6 | | 11.3 |
| Aldolase | c44217_g1 | | 17.3 | | 6.46 | | 9.52 |
| Aldolase | c36912_g1 | | 3.97 | | 6.65 | | 6.6 |
| Aldolase | c70709_g1 | | 0.06 | | 0.06 | | 4.38 |
| Aldolase | c54429_g1 | | 10.24 | | 5.46 | | 4.3 |
| Aldolase | c51966_g1 | | 7.59 | | 6.26 | | 3.73 |
| Aldolase | c4114_g1 | | 4.39 | | 1.75 | | 3.14 |
| Aldolase | c42327_g1 | | 66.35 | | 0.06 | | 2.45 |
| Aldolase | c37535_g1 | | 1.23 | | 3.81 | | 1.81 |
| Aldolase | c37881_g1 | | 2.76 | | 0.5 | | 1.65 |
| Aldolase | c46107_g2 | | 13.64 | | 1.87 | | 1.49 |
| Aldolase | c42327_g2 | | 39.8 | | 0.06 | | 1.41 |
| Aldolase | c15693_g1 | | 11.38 | | 0.06 | | 0.8 |
| Aldolase | c4240_g1 | | 6.61 | | 2.6 | | 0.06 |
| aldose 1-epimerase | c49073_g1 | | 11.02 | | 21 | | 2.95 |
| aldose 1-epimerase | c45999_g1 | | 5.64 | | 7.02 | | 5.45 |
| aldose 1-epimerase | c82023_g1 | | 1.3 | | 0.06 | | 0.17 |
| dihydrolipoyl dehydrogenase | c50921_g1 | | 84.24 | | 217.05 | | 176.45 |
| dihydrolipoyl dehydrogenase | c16645_g1 | | 63.58 | | 98.96 | | 57.28 |
| dihydrolipoyl dehydrogenase | c49572_g1 | | 20.15 | | 25.27 | | 13.5 |
| dihydrolipoyl dehydrogenase | c23491_g1 | | 8.67 | | 12.73 | | 7.08 |
| dihydrolipoyl dehydrogenase | c30692_g1 | | 8.54 | | 0.06 | | 1.04 |
| dihydrolipoyl dehydrogenase | c64325_g1 | | 6.66 | | 0.06 | | 0.06 |
| dihydrolipoyllysine-residue acetyltransferase | c46877_g1 | | 52.14 | | 102.38 | | 63.33 |
| dihydrolipoyllysine-residue acetyltransferase | c54084_g1 | | 10.66 | | 26.57 | | 18.34 |
| dihydrolipoyllysine-residue acetyltransferase | c52991_g1 | | 14.54 | | 25.78 | | 15.16 |
| dihydrolipoyllysine-residue acetyltransferase | c40534_g1 | | 31.12 | | 22.34 | | 13.72 |
| dihydrolipoyllysine-residue acetyltransferase | c46160_g1 | | 32.75 | | 16.13 | | 9.59 |
| dihydrolipoyllysine-residue acetyltransferase | c46160_g2 | | 19.13 | | 8.2 | | 7.54 |
| dihydrolipoyllysine-residue acetyltransferase | c78401_g1 | | 2.35 | | 0.06 | | 0.06 |
| Enolase | c47891_g1 | | 548.04 | | 725.13 | | 765.47 |
| Enolase | c52109_g1 | | 141.96 | | 69.07 | | 74.88 |
| Enolase | c28767_g1 | | 106.83 | | 235.68 | | 395.28 |

**Table S5**. *Cont*.

| Gene Name Used in This Research | | Contig ID | | FPKM_WT | | FPKM_As1 | FPKM_As2 |
| --- | --- | --- | --- | --- | --- | --- | --- |
| Enolase | c38364_g1 | | 59.77 | | 23.32 | | 21.22 |
| Enolase | c9092_g1 | | 46.38 | | 6.78 | | 8.92 |
| Enolase | c43880_g1 | | 46.15 | | 83.6 | | 191.89 |
| Enolase | c38255_g1 | | 25.34 | | 30.07 | | 42.61 |
| Enolase | c27391_g1 | | 10.93 | | 17.97 | | 14.98 |
| Enolase | c57618_g1 | | 7.07 | | 3.15 | | 1.12 |
| Enolase | c38255_g2 | | 5.99 | | 2.9 | | 3.6 |
| Enolase | c37731_g1 | | 2.88 | | 1.01 | | 0.66 |
| Enolase | c82231_g1 | | 2.64 | | 1.62 | | 0.62 |
| Enolase | c34851_g1 | | 0.06 | | 0.06 | | 8.23 |
| fructose-bisphosphate aldolase | c51172_g1 | | 298.03 | | 869.52 | | 643.06 |
| fructose-bisphosphate aldolase | c51754_g1 | | 343.52 | | 507.6 | | 595.63 |
| fructose-bisphosphate aldolase | c52127_g1 | | 142.18 | | 57.26 | | 69.24 |
| fructose-bisphosphate aldolase | c52127_g2 | | 216.05 | | 44.05 | | 77.87 |
| fructose-bisphosphate aldolase | c31039_g1 | | 439.05 | | 33.46 | | 143.37 |
| fructose-bisphosphate aldolase | c51966_g1 | | 7.59 | | 6.26 | | 3.73 |
| fructose-bisphosphate aldolase | c54429_g1 | | 10.24 | | 5.46 | | 4.3 |
| fructose-bisphosphate aldolase | c42327_g1 | | 66.35 | | 0.06 | | 2.45 |
| fructose-bisphosphate aldolase | c42327_g2 | | 39.8 | | 0.06 | | 1.41 |
| fructose-bisphosphate aldolase | c15693_g1 | | 11.38 | | 0.06 | | 0.8 |
| fructose-bisphosphate aldolase | c70709_g1 | | 0.06 | | 0.06 | | 4.38 |
| glucose-6-phosphate isomerase | c50453_g1 | | 157.19 | | 200.55 | | 178.24 |
| glucose-6-phosphate isomerase | c51760_g1 | | 67.95 | | 127.39 | | 134.97 |
| glucose-6-phosphate isomerase | c28438_g1 | | 16.45 | | 33.29 | | 45.39 |
| glucose-6-phosphate isomerase | c28438_g2 | | 6.93 | | 54.39 | | 31.25 |
| glucose-6-phosphate isomerase | c34689_g1 | | 8.57 | | 19.51 | | 15.55 |
| glucose-6-phosphate isomerase | c24892_g1 | | 13.77 | | 8.02 | | 14.44 |
| glucose-6-phosphate isomerase | c22150_g1 | | 8.47 | | 0.06 | | 2.9 |
| glucose-6-phosphate isomerase | c57520_g1 | | 1.18 | | 0.06 | | 0.06 |
| glyceraldehyde-3-phosphate dehydrogenase | c48350_g1 | | 865.68 | | 1324.83 | | 2141.85 |
| glyceraldehyde-3-phosphate dehydrogenase | c52826_g1 | | 27.17 | | 64.99 | | 72 |
| glyceraldehyde-3-phosphate dehydrogenase | c33474_g1 | | 29.93 | | 16.16 | | 29.28 |
| glyceraldehyde-3-phosphate dehydrogenase | c50351_g1 | | 54.48 | | 25.05 | | 27.18 |
| glyceraldehyde-3-phosphate dehydrogenase | c45542_g3 | | 57.46 | | 7.45 | | 21.11 |
| glyceraldehyde-3-phosphate dehydrogenase | c46068_g2 | | 11.9 | | 9.03 | | 18.76 |
| glyceraldehyde-3-phosphate dehydrogenase | c23835_g1 | | 13.68 | | 9.96 | | 9.52 |
| glyceraldehyde-3-phosphate dehydrogenase | c72867_g1 | | 11.71 | | 1.75 | | 9.41 |
| glyceraldehyde-3-phosphate dehydrogenase | c38401_g1 | | 30.24 | | 8.45 | | 8.3 |
| glyceraldehyde-3-phosphate dehydrogenase | c21615_g1 | | 2.55 | | 0.06 | | 5.27 |
| glyceraldehyde-3-phosphate dehydrogenase | c8660_g1 | | 2.21 | | 1.33 | | 3.62 |
| glyceraldehyde-3-phosphate dehydrogenase | c5283_g1 | | 5.92 | | 3.74 | | 3.6 |
| glyceraldehyde-3-phosphate dehydrogenase | c25512_g1 | | 4.24 | | 3.37 | | 3.03 |

**Table S5**. *Cont*.

| Gene Name Used in This Research | | Contig ID | | FPKM_WT | | FPKM_As1 | FPKM_As2 |
| --- | --- | --- | --- | --- | --- | --- | --- |
| glyceraldehyde-3-phosphate dehydrogenase | c24362_g1 | | 1.38 | | 1.26 | | 2.8 |
| glyceraldehyde-3-phosphate dehydrogenase | c52826_g2 | | 37.76 | | 5.9 | | 2.06 |
| glyceraldehyde-3-phosphate dehydrogenase | c44939_g1 | | 7.09 | | 1.01 | | 0.7 |
| glyceraldehyde-3-phosphate dehydrogenase | c45181_g1 | | 12.25 | | 0.36 | | 0.12 |
| glyceraldehyde-3-phosphate dehydrogenase | c71706_g1 | | 2.39 | | 0.42 | | 0.06 |
| Hexokinase | c53053_g1 | | 570.88 | | 405.41 | | 249.97 |
| Hexokinase | c53074_g1 | | 45.64 | | 80.03 | | 66.02 |
| Hexokinase | c51565_g2 | | 14.27 | | 6.75 | | 5.45 |
| Hexokinase | c13631_g1 | | 7.71 | | 6.85 | | 16.24 |
| Hexokinase | c51565_g3 | | 7.64 | | 3.82 | | 3.66 |
| Hexokinase | c33639_g2 | | 5.78 | | 6.61 | | 2.68 |
| Hexokinase | c37441_g1 | | 4.8 | | 5.1 | | 4.84 |
| Hexokinase | c41304_g1 | | 3.2 | | 8.08 | | 10.52 |
| Hexokinase | c33639_g1 | | 2.78 | | 3.4 | | 1.06 |
| Hexokinase | c36_g1 | | 1.22 | | 0.53 | | 1.48 |
| Hexokinase | c17952_g1 | | 1.04 | | 2.54 | | 0.59 |
| Hexokinase | c51565_g4 | | 0.87 | | 0.7 | | 0.65 |
| Hexokinase | c55847_g1 | | 0.06 | | 0.06 | | 0.06 |
| phosphoenolpyruvate carboxykinase | c44397_g1 | | 25.9 | | 2.48 | | 14.6 |
| phosphoenolpyruvate carboxykinase | c45559_g2 | | 14.43 | | 4.26 | | 18.52 |
| phosphoenolpyruvate carboxykinase | c45559_g1 | | 12.02 | | 6.64 | | 10.53 |
| phosphoglucomutase | c53381_g1 | | 158.83 | | 28.34 | | 37.93 |
| phosphoglucomutase | c52097_g1 | | 106.56 | | 164.44 | | 141.24 |
| phosphoglucomutase | c7447_g1 | | 19 | | 85 | | 44.34 |
| pyruvate decarboxylase | c51779_g3 | | 8.77 | | 316.5 | | 1322.58 |
| pyruvate decarboxylase | c51779_g1 | | 3.81 | | 212.12 | | 1207.98 |
| pyruvate decarboxylase | c83130_g1 | | 37.14 | | 256.98 | | 568.57 |
| pyruvate decarboxylase | c32049_g1 | | 20.97 | | 173.09 | | 491.86 |
| pyruvate decarboxylase | c37393_g1 | | 5.56 | | 58.72 | | 106.65 |
| pyruvate decarboxylase | c21554_g1 | | 0.06 | | 0.06 | | 73.14 |
| pyruvate decarboxylase | c18202_g2 | | 11.43 | | 4.66 | | 59.95 |
| pyruvate decarboxylase | c18202_g1 | | 2.31 | | 2.72 | | 48.08 |
| pyruvate decarboxylase | c44265_g1 | | 7.07 | | 2.4 | | 42.24 |
| pyruvate decarboxylase | c51779_g2 | | 0.06 | | 38.45 | | 37.97 |
| pyruvate decarboxylase | c42062_g1 | | 7.29 | | 2.03 | | 22.92 |
| pyruvate decarboxylase | c57466_g1 | | 0.06 | | 0.06 | | 14.15 |
| pyruvate decarboxylase | c15104_g1 | | 0.43 | | 4.76 | | 2.99 |
| pyruvate decarboxylase | c36993_g1 | | 3.96 | | 0.06 | | 0.06 |
| pyruvate decarboxylase | c85050_g1 | | 1.94 | | 0.06 | | 0.06 |
| pyruvate dehydrogenase E1 component | c42176_g1 | | 127.4 | | 242.01 | | 221.9 |
| pyruvate dehydrogenase E1 component | c42427_g1 | | 25.07 | | 139.52 | | 115.18 |
| pyruvate dehydrogenase E1 component | c47311_g1 | | 137.22 | | 50.91 | | 63.29 |

**Table S5**. *Cont*.

| Gene Name Used in This Research | | Contig ID | | FPKM_WT | | FPKM_As1 | FPKM_As2 |
| --- | --- | --- | --- | --- | --- | --- | --- |
| pyruvate dehydrogenase E1 component | c39393_g1 | | 19.37 | | 33.31 | | 26.04 |
| pyruvate dehydrogenase E1 component | c39393_g3 | | 20.53 | | 27.96 | | 20.3 |
| pyruvate dehydrogenase E1 component | c49778_g1 | | 20.67 | | 23.51 | | 16.15 |
| pyruvate dehydrogenase E1 component | c39393_g2 | | 18.65 | | 20.08 | | 12.29 |
| pyruvate dehydrogenase E1 component | c19055_g1 | | 4.14 | | 4.78 | | 10.72 |
| pyruvate dehydrogenase E1 component | c25298_g1 | | 3.72 | | 2.49 | | 6.3 |
| pyruvate dehydrogenase E1 component | c20468_g2 | | 24.83 | | 7 | | 6.02 |
| pyruvate dehydrogenase E1 component | c78173_g1 | | 15.97 | | 3.7 | | 5.26 |
| pyruvate dehydrogenase E1 component | c49778_g2 | | 11.13 | | 1.98 | | 3.73 |
| pyruvate dehydrogenase E1 component | c25298_g2 | | 1.11 | | 0.06 | | 2.37 |
| pyruvate dehydrogenase E1 component | c20468_g1 | | 4.12 | | 1.99 | | 1.83 |
| pyruvate dehydrogenase E1 component | c4536_g1 | | 1.14 | | 0.44 | | 1.2 |
| pyruvate dehydrogenase E1 component | c8036_g1 | | 0.73 | | 0.06 | | 0.82 |
| pyruvate dehydrogenase E1 component | c6393_g1 | | 2.51 | | 0.06 | | 0.06 |
| pyruvate dehydrogenase E1 component | c6393_g2 | | 1.12 | | 0.06 | | 0.06 |
| pyruvate kinase | c54264_g1 | | 263.3 | | 386.98 | | 318.59 |
| pyruvate kinase | c44686_g1 | | 292.52 | | 155.13 | | 116.96 |
| pyruvate kinase | c18336_g1 | | 9.07 | | 15.68 | | 81.39 |
| pyruvate kinase | c53603_g1 | | 44.2 | | 63.71 | | 54.72 |
| pyruvate kinase | c50745_g2 | | 16.57 | | 29.97 | | 37.39 |
| pyruvate kinase | c50745_g1 | | 1.69 | | 34.63 | | 30.8 |
| pyruvate kinase | c24556_g2 | | 27.91 | | 53.83 | | 30.28 |
| pyruvate kinase | c44669_g1 | | 1.42 | | 3 | | 29.27 |
| pyruvate kinase | c32705_g1 | | 0.06 | | 1.69 | | 27.38 |
| pyruvate kinase | c53603_g3 | | 25.8 | | 27.83 | | 25.38 |
| pyruvate kinase | c24556_g1 | | 32.34 | | 89.74 | | 24.04 |
| pyruvate kinase | c44508_g1 | | 25.62 | | 33.37 | | 23.17 |
| pyruvate kinase | c19670_g1 | | 176.8 | | 224.72 | | 22.61 |
| pyruvate kinase | c53603_g2 | | 43.08 | | 38.9 | | 22.58 |
| pyruvate kinase | c10581_g1 | | 2.51 | | 1.5 | | 21.71 |
| pyruvate kinase | c44924_g2 | | 0.06 | | 0.2 | | 20.67 |
| pyruvate kinase | c22231_g1 | | 0.06 | | 0.06 | | 20.56 |
| pyruvate kinase | c35789_g1 | | 5.71 | | 4.27 | | 20.35 |
| pyruvate kinase | c48578_g1 | | 9.15 | | 13.29 | | 20.24 |
| pyruvate kinase | c62388_g1 | | 78.59 | | 56.16 | | 20.15 |
| pyruvate kinase | c24375_g1 | | 43.02 | | 38.28 | | 19.81 |
| pyruvate kinase | c32319_g1 | | 24.64 | | 21.36 | | 18.95 |
| pyruvate kinase | c44924_g1 | | 0.06 | | 1.36 | | 15.43 |
| pyruvate kinase | c18545_g1 | | 13.57 | | 31.04 | | 12.03 |
| pyruvate kinase | c22777_g2 | | 8.41 | | 5.64 | | 7.21 |
| pyruvate kinase | c47938_g2 | | 61.49 | | 9.01 | | 5.93 |
| pyruvate kinase | c46486_g1 | | 9.12 | | 4.76 | | 5.76 |

**Table S5**. *Cont*.

| Gene Name Used in This Research | Contig ID | FPKM_WT | FPKM_As1 | FPKM_As2 |
| --- | --- | --- | --- | --- |
| pyruvate kinase | c35852_g1 | 3.5 | 2.62 | 3.52 |
| pyruvate kinase | c46486_g2 | 8.62 | 4.64 | 3.08 |
| pyruvate kinase | c77144_g1 | 1.27 | 2.47 | 2.18 |
| pyruvate kinase | c35572_g1 | 0.72 | 0.89 | 1.53 |
| pyruvate kinase | c31561_g2 | 10.03 | 6.2 | 1.4 |
| pyruvate kinase | c23376_g1 | 0.59 | 1.44 | 0.67 |
| pyruvate kinase | c80802_g1 | 1.16 | 0.47 | 0.45 |
| pyruvate kinase | c61761_g1 | 1.7 | 2.3 | 0.4 |
| pyruvate kinase | c33367_g1 | 79.99 | 8.44 | 0.06 |
| pyruvate kinase | c4284_g1 | 0.06 | 2.92 | 0.06 |
| pyruvate kinase | c10165_g1 | 1.21 | 0.99 | 0.06 |
| pyruvate kinase | c22777_g1 | 7.49 | 0.06 | 0.06 |
| invertase | c70839_g1 | 1.06 | 2.55 | 0.000001 |
| invertase | c71850_g1 | 0.000001 | 3.31 | 0.000001 |
| invertase | c28604_g1 | 0.54 | 2.75 | 0.53 |
| invertase | c28269_g2 | 0.000001 | 1.29 | 0.61 |
| invertase | c22356_g1 | 0.62 | 3.41 | 0.73 |
| invertase | c55383_g1 | 0.66 | 0.27 | 0.79 |
| invertase | c79871_g1 | 0.000001 | 0.000001 | 1.31 |
| invertase | c28269_g1 | 0.000001 | 12.23 | 2.4 |
| invertase | c46063_g1 | 0.52 | 2.19 | 2.5 |
| invertase | c38670_g2 | 4.63 | 8.34 | 2.52 |
| invertase | c53621_g1 | 10.24 | 10.06 | 4.95 |
| invertase | c39820_g1 | 0.000001 | 7.39 | 5.44 |
| invertase | c33403_g1 | 18.24 | 6.06 | 6.37 |
| invertase | c53621_g2 | 3.04 | 14.83 | 6.63 |
| invertase | c36736_g1 | 3 | 6.82 | 9.36 |
| invertase | c49970_g2 | 17.56 | 14.96 | 10.26 |
| invertase | c16051_g1 | 5.29 | 25.46 | 11.58 |
| invertase | c65845_g1 | 8.26 | 41.88 | 20.99 |
| invertase | c34503_g1 | 6.56 | 21.45 | 25.36 |
| invertase | c41929_g1 | 4.57 | 10.88 | 29.15 |
| invertase | c40034_g1 | 97.89 | 71.65 | 39.83 |
| invertase | c51506_g1 | 22.11 | 43.47 | 43.17 |
| invertase | c48929_g1 | 7.76 | 130.87 | 171.55 |
| invertase | c35606_g1 | 243.06 | 3830.11 | 7726.81 |
